# Design, Synthesis, and Biological Evaluation of Some Novel Pyrrolizine Derivatives as COX Inhibitors with Anti-Inflammatory/Analgesic Activities and Low Ulcerogenic Liability

**DOI:** 10.3390/molecules21020201

**Published:** 2016-02-08

**Authors:** Ahmed M. Gouda, Hamed I. Ali, Waleed H. Almalki, Mohamed A. Azim, Mohammed A. S. Abourehab, Ahmed H. Abdelazeem

**Affiliations:** 1 Department of Pharmaceutical Chemistry, Faculty of Pharmacy, Umm Al-Qura University, Makkah 21955, Saudi Arabia; hamed_ali37@yahoo.com (H.I.A.); mohammadazim97@yahoo.com (M.A.A.); 2 Department of Medicinal Chemistry, Faculty of Pharmacy, Beni-Suef University, Beni-Suef 62514, Egypt; ahmed.abdelazeem@pharm.bsu.edu.eg; 3 Rangel College of Pharmacy, Health Science Center, Texas A & M University, Kingsville, TX 78363, USA; 4 Department of Pharmacology, Faculty of Pharmacy, Umm Al-Qura University, Makkah 21955, Saudi Arabia; whmalki@uqu.edu.sa; 5 Department of Pharmaceutical Chemistry, Faculty of Pharmacy, Cairo University, Cairo 11562, Egypt; 6 Department of Pharmaceutics, Faculty of Pharmacy, Umm Al-Qura University, Makkah 21955, Saudi Arabia; maabourehab@uqu.edu.sa

**Keywords:** pyrrolizine, anti-inflammatory, analgesic, COX, 5-LOX, ulcerogenicity

## Abstract

Non-steroidal anti-inflammatory drugs (NSAIDs) are the most commonly prescribed anti-inflammatory and pain relief medications. However, their use is associated with many drawbacks, including mainly serious gastric and renal complications. In an attempt to circumvent these risks, a set of *N*-(4-bromophenyl)-7-cyano-6-substituted-*H*-pyrrolizine-5-carboxamide derivatives were designed, synthesized and evaluated as dual COX/5-LOX inhibitors. The structural elucidation, *in vivo* anti-inflammatory and analgesic activities using a carrageenan-induced rat paw edema model and hot plate assay, were performed, respectively. From the results obtained, it was found that the newly synthesized pyrrolizines exhibited IC_50_ values in the range of 2.45–5.69 µM and 0.85–3.44 µM for COX-1 and COX-2, respectively. Interestingly, compounds **12**, **13**, **16** and **17** showed higher anti-inflammatory and analgesic activities compared to ibuprofen. Among these derivatives, compounds **16** and **19** displayed better safety profile than ibuprofen in acute ulcerogenicity and histopathological studies. Furthermore, the docking studies revealed that compound **17** fits nicely into COX-1 and COX-2 binding sites with the highest binding affinity, while compound **16** exerted the highest binding affinity for 5-LOX. In light of these findings, these novel pyrrolizine-5-carboxamide derivatives represent a promising scaffold for further development into potential dual COX/5-LOX inhibitors with safer gastric profile.

## 1. Introduction

NSAIDs are some of the most frequently used medications in the world [1]. Their widespread use is usually associated with several serious side effects and complications [2,3,4]. Erosion and bleeding of the gastric mucosa are the most prevalent risks. Many of these complications can be life-threatening and several mortalities have been reported [4,5]. The gastrointestinal toxicity of NSAIDs is mediated either by the inhibition of the cytoprotective PGs biosynthesis [6] or by the induction of GIT necrotic effects [7]. These necrotic effects were detected for selective and nonselective COX inhibitors [8], even at low prophylactic doses [9].

**Figure 1:**
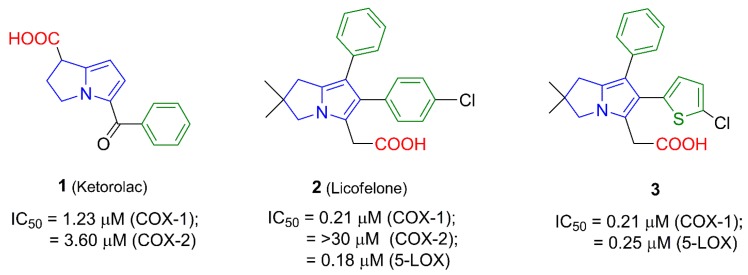
The IC_50_ values of pyrrolizine-based anti-inflammatory agents **1**–**3** linked with a carboxylic acid moiety.

To date, there is no safe nonsteroidal anti-inflammatory agent that could be used in patients with peptic ulcers, renal failure or asthma. However, on reviewing the literature, several strategies were found to afford GIT safer anti-inflammatory agents. Of these strategies, conversion of the acidic COX inhibitors into non-acidic prodrugs [10], improving selectivity for the COX-2 enzyme [11] and development of nitric oxide releasing NSAIDs [12] were the most commonly utilized. Recently, introducing dual COX/5-LOX inhibitors were the focus of researchers in order to develop safer anti-inflammatory agents [13]. Several pyrrolizine derivatives (Figure 1) were identified to have anti-inflammatory, analgesic, and antipyretic activities. Of these derivatives, ketorolac (**1**) is a nonselective COX-1/2 inhibitor with a selectivity ratio of 2.9 for COX-1 [14]. Owing to its potent analgesic activity, ketorolac is often used in the treatment of severe neuropathic pain [15]. Moreover, licofelone (**2**) acts as a dual COX/5-LOX inhibitor with IC_50_ values in the submicromolar range [16]. Interestingly, licofelone revealed anti-inflammatory and analgesic efficiency in asthmatic patients. Compared to other COX/5-LOX inhibitors which showed also good GIT tolerance, licofelone is the only COX/5-LOX inhibitor that showed an acceptable safety level in clinical use [17]. The replacement of the 4-chlorophenyl group at C5 in licofelone with a 5-chlorothiophen-2-yl moiety in compound **3** resulted in almost the same COX-1/5-LOX inhibitory activity [18].

**Figure 2:**
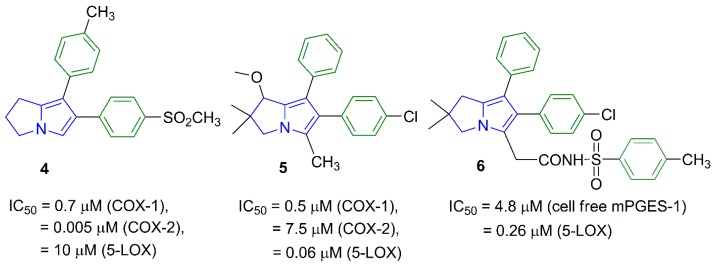
The IC_50_ values of pyrrolizine-based anti-inflammatory agents **4**–**6** lacking the free carboxylic acid group.

In order to avoid the gastric side effects, several reported pyrrolizines with nonselective COX inhibitory activity lacking the distinctive free carboxylic group were designed [19,20,21]. For instance, the diphenylpyrrolizine **4** [19] and the 1-methoxypyrrolizine **5** [20] showed anti-inflammatory activity mediated by COX/5-LOX inhibition. Additionally, masking the carboxylic group in licofelone (**2**) using a tolylsulfonimide moiety as in compound **6** retained the 5-LOX inhibition (IC_50_ = 0.26 µM) and increased the mPGES-1 inhibitory activity [21], as shown in Figure 2.

**Figure 3:**
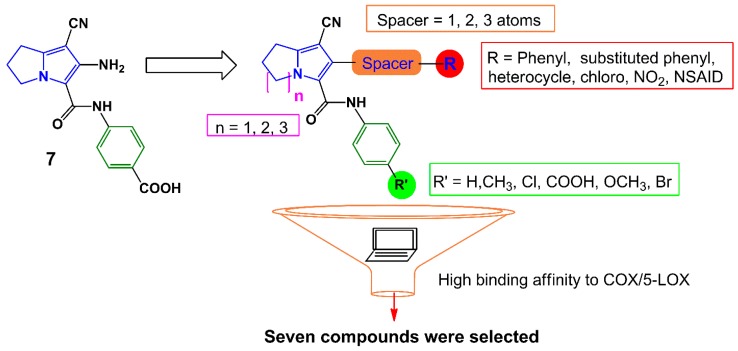
Design strategies for compounds **12**–**18**.

Earlier, we have reported several pyrrolizine derivatives with moderate to high anti-inflammatory activities [22]. In this report, compound **7** exhibited approximately 50% reduction in rat paw edema in comparison with ketorolac (Figure 3). Prompted by the aforementioned findings, we aimed in this work to develop novel potent anti-inflammatory and analgesic agents with low GIT side effects. We have designed several different analogs based on our previously reported anti-inflammatory agent **7** using a lead optimization approach in drug design. In doing so, the free carboxylic group in compound **7** was replaced with other non-acidic fragments (R′) to avoid the direct gastric insult complications of the carboxylic group.

**Scheme 1:**
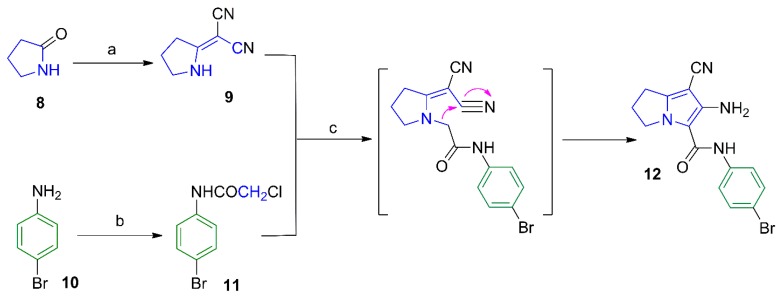
Synthesis of compound **12**. *Reagents and* *reaction conditions*: (**a**) (CH_3_)_2_SO_4_, benzene, CH_2_(CN)_2_; (**b**) ClCH_2_COCl, AcOH, CH_3_COONa; (**c**) acetone, K_2_CO_3_, reflux, 24 h.

**Scheme 2:**
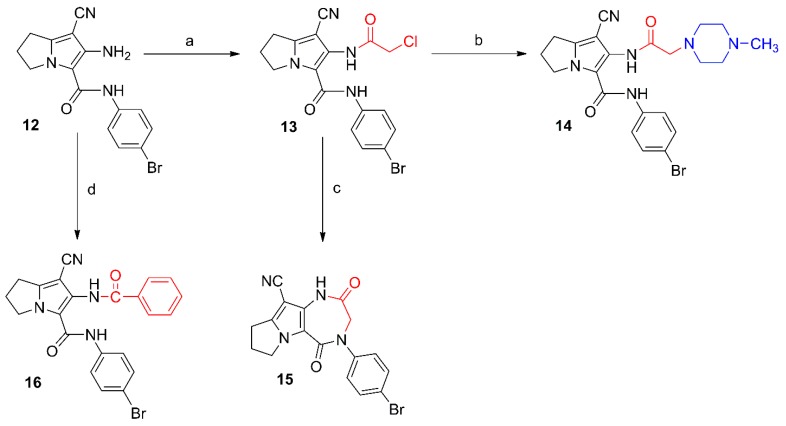
Synthesis of compounds **13**–**16**. *Reagents and* *reaction conditions*: (**a**) ClCH_2_COCl, benzene, 48 h; (**b**) 1-methylpiperazine, NaHCO_3_, absolute ethanol, reflux, 6 h; (**c**) KHCO_3_, DMF, rt, 48 h; (**d**) benzoyl chloride, benzene, rt, 48 h.

**Scheme 3:**
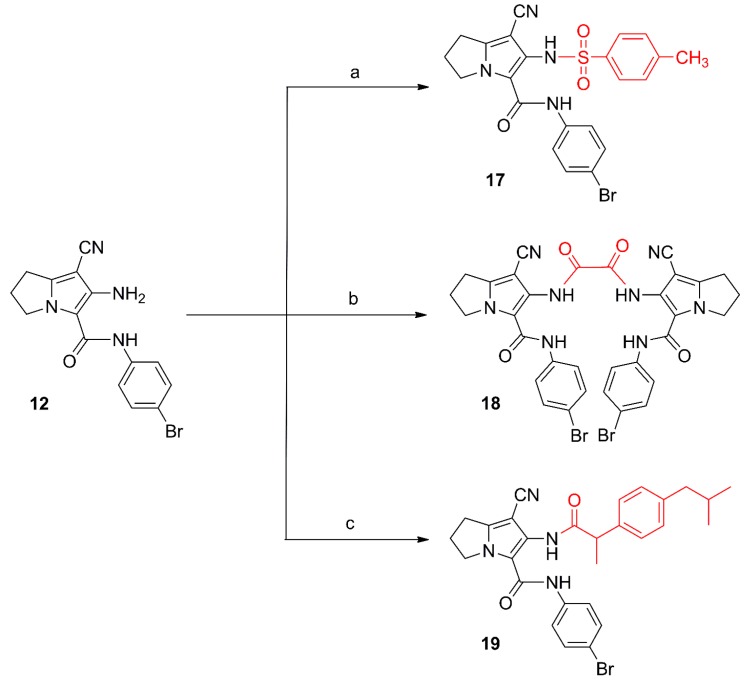
Synthesis of compounds **17**–**19**. *Reagents and reaction conditions*: (**a**) *p*-toluenesulfonyl chloride, acetone, K_2_CO_3_; rt, 12 h; (**b**) oxalyl chloride, dry acetone, rt, 24 h; (**c**) 1. ibuprofen, SOCl_2_; 2. compound **12**, dry benzene, rt, 24 h.

The impact of the electronic effect of these substituents (R′) on COX-1/-2 binding patterns was evaluated by a molecular docking study. Moreover, the variation in the length of the spacer between the pyrrolizine ring and the terminal (R) moiety was conducted. Seven compounds **12**–**18** from the virtual designed library were selected for synthesis (Scheme 1, Scheme 2 and Scheme 3) and biological evaluation based on an initial filtration process using the docking tool against COX enzymes (Figure 3). Finally, a hybrid compound **19** composed of the pyrrolizine precursor **12** and ibuprofen was designed to evaluate the possible synergism resulted from the combination of these two compounds into one scaffold.

## 2. Results and Discussion

### 2.1. Chemistry

The synthesis of compounds **9** [23] and **11** [24] described in Scheme 1 involved previously reported procedures. The pyrrolizine **12** was obtained from the reaction of *N*-(4-bromophenyl)-2-chloroacetamide (**11**) with 2-(pyrrolidin-2-ylidene)malononitrile (**9**) as reported before [25]. The IR spectrum of compound **12** revealed a stretching band at 2218 cm^−1^ for the cyano group, in addition to an absorption band at 1652 cm^−1^ assigned for the carbonyl group (Figure S1). The ^1^H-NMR spectrum of compound **12** revealed three signals at δ 2.55, 3.00 and 4.41 ppm indicating the three CH_2_ groups of the pyrrolizine nucleus in addition to two singlet signals at δ 3.57 and 9.64 ppm attributed to the NH_2_ and NH protons, respectively (Figure S17a–c). The ^13^C-NMR spectrum revealed 13 signals and DEPT-135 spectrum was used to differentiate between primary, secondary and tertiary carbon atoms (Figures S18a–c and S19). The mass spectrum of compound **12** revealed the expected molecular ion peak at *m*/*z* 344 (Figure S9).

Compounds **13** and **16** were prepared by acylation of the amino group at C6 in the parent compound **12** using chloroacetyl chloride and benzoyl chloride, respectively (Scheme 2). The chloro group in compound **13** was subjected to a nucleophilic substitution with *N*-methylpiperazine to afford compounds **14**. The structure of compound **13** was confirmed by its ^1^H-NMR spectrum where a singlet signal at δ 4.33 ppm was assigned for the CH_2_-Cl protons and two other singlets at δ 9.59 and 10.28 ppm were assigned for the two amidic protons (Figure S20a–c), whereas the ^1^H-NMR spectrum of compound **14** showed an additional singlet at δ 2.34 for its N-CH_3_, and two multiplets at δ 2.57 and 2.75 ppm attributed to the eight piperazine protons (Figure S22a–c). The ^13^C-NMR of compound **14** revealed the aliphatic carbons of the pyrrolizine and piperazine rings in the δ 25.02–61.16 ppm range (Figure S23a–c).

Moreover, stirring compound **13** in DMF furnished the diazepine derivative **15** via an intramolecular cyclization reaction. The structure of compound **15** was elucidated using different analytical methods. The ^1^H-NMR spectrum of compound **15** revealed only one singlet at δ 10.37 ppm corresponding to the protons of the CONH function (Figure S24a–c), while the ^13^C-NMR spectrum revealed two signals at δ 164.86 and 172.64 ppm assigned to the two carbonyl groups (Figure S25a–c). On the other hand, compound **16** was obtained by acylation of the parent pyrrolizine **12** with benzoyl chloride. The ^1^H-NMR spectrum of compound **16** revealed the two amide protons as singlets at δ 8.04 and 9.98 ppm (Figure S26a–c), while the ^13^C-NMR spectrum revealed the two carbonyl carbons at δ 157.54 and 169.18 ppm (Figure S27a–c). The mass spectra of compounds **13**, **15**, and **16** showed the corresponding molecular ion peaks at 420, 384 and 448, respectively (Figures S10, S12 and S15).

6-(4-Methylphenylsulfonamido)-2,3-dihydro-1*H*-pyrrolizine (**17**), the dimer **18** and the hybrid **19** were obtained from the reaction of compound **12** with 4-tolylsulfonyl chloride, oxalyl chloride and the acid chloride of ibuprofen, respectively (Scheme 3). The structural elucidation of compounds **17**–**19** was done using spectral and elemental analysis. The IR spectra of compounds **17**–**19** revealed stretching bands at the range of 2222–2231 cm^−1^ indicating a cyano group, and other absorption bands at the 1660–1746 cm^−1^ range indicating the carbonyl groups (Figures S6–S8). The ^1^H-NMR spectra of compounds **17**–**19** displayed two signals in each compound in the δ 6.57–10.72 ppm range assigned to the protons in the NH groups, in addition to a singlet at δ 2.49 ppm attributed to the methyl protons in compound **17** (Figures S28, S30 and S33). Two signals at the range of δ 157.47–177.14 ppm in the ^13^C-NMR spectrum of compound **19** were due to the two carbonyl carbons (Figure S34a–c). The mass spectra of compounds **17**, **18** and **19** all showed the molecular ion peaks at 498, 742, and 532, respectively (Figures S14–S16).

### 2.2. Biological Evaluation

#### 2.2.1. *In Vitro* COX Inhibitory Assay

COX inhibition is the main mechanism of action of NSAIDs so far. The ability of the new compounds **12**–**18** to inhibit COX enzymes was determined in an attempt to investigate their mechanism of action. Both COX-1 and COX-2 inhibitory activities were evaluated using a COX colorimetric inhibitor screening assay kit (Catalog No. 701050, Cayman Chemical Inc., Ann Arbor, MI, USA) according to the previous reports [26,27,28]. The pyrrolizines **12**–**18** were tested against indomethacin (as a nonselective COX inhibitor) and celecoxib (as a selective COX-2 inhibitor). The results were expressed in terms of IC_50_ values and COX-1/COX-2 selectivity index (SI) was calculated, (Table 1). The results demonstrated that the newly synthesized compounds **12**–**18** have IC_50_ values in the range of 2.45–5.69 µM for COX-1 and 0.85–3.44 µM for COX-2. It was noteworthy that compounds **12**–**18** showed good COX-2 selectivity over COX-2, with selectivity indexes in the 2.89–6.03 range.

**Table 1:** *In vitro* COX-1/2 enzymes inhibition results of compounds **12**–**18**.

Compd. No.	COX-1	COX-2	*SI* ^b^
	(IC_50_ µM) ^a^	(IC_50_ µM) ^a^	
**12**	4.64	1.27	3.64
**13**	5.69	1.64	3.48
**14**	3.50	1.09	3.21
**15**	3.37	1.06	3.17
**16**	2.45	0.85	2.89
**17**	5.01	1.72	2.91
**18**	5.10	0.85	6.03
**Indomethacin**	0.73	32.6	0.02
**Celecoxib**	15.6	0.32	48.75

**^a^** IC_50_ was calculated using three determinations for COX-1 (ovine) and COX-2 (human recombinant) screening assay kit (Cat. No 701050, Cayman Chemical Inc.); **^b^** *in vitro* COX-2 selectivity index (*SI*) = IC_50_ of COX-1/IC_50_ of COX-2.

#### 2.2.2. *In Vivo* Biological Evaluation

##### Anti-Inflammatory Activity

**Figure 4:**
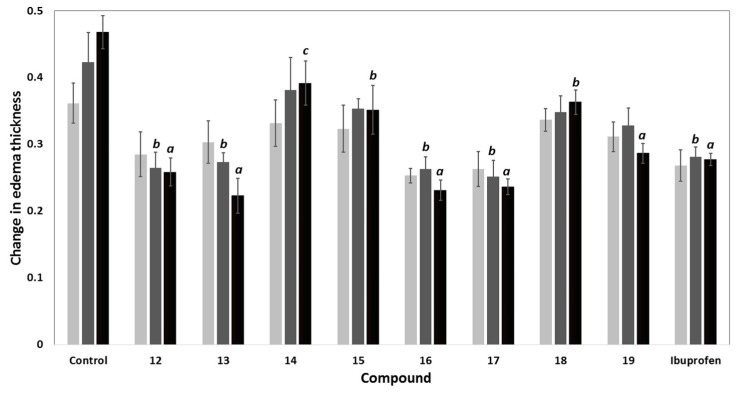
Change in edema thickness using carrageenan-induced rat paw edema mode of compounds **12**–**19** at 1 h (${}^{▄}$), 2 h (${}^{▄}$) and 3 h (${}^{▄}$) after induction of inflammation; data expressed as mean ± SEM, (*n* = 6); data were analyzed by One way ANOVA followed by student-Newman-Keuls multiple comparison test; ***^a^*** statistically significant from control (*p* < 0.001); ***^b^*** statistically significant from control (*p* < 0.01); ***^c^*** statistically significant from control (*p* < 0.05).

The carrageenan-induced rat paw edema model was utilized to investigate the anti-inflammatory activity of compounds **12**–**19** relative to ibuprofen as a reference drug [29]. The mean changes in edema thickness and anti-inflammatory activities of compounds **12**–**19** are presented in Figure 4 and Table 2. The parent compound **12** displayed higher anti-inflammatory activity than that of ibuprofen. The additional 2-chloroacetyl, benzoyl and 4-tolylsulfonyl moieties in compounds **13**, **16**, and **17**, respectively, resulted in a remarkable increase in the anti-inflammatory activities compared to compound **12**. The replacement of the chloro atom in compound **13** by a 4-methylpiperazin-1-yl in compound **14** resulted in a sharp decrease in the anti-inflammatory activity. Moreover, the dimer **18** showed lower activity than both ibuprofen and the parent compound **12**, while the hybrid **19** showed weak activity after the first hour. However, an increase in the activity was observed after three hours to be comparable to that of ibuprofen. Among all the newly synthesized compounds, 6-(2-chloroacetamido)-pyrrolizine-5-carboxamide (**13**) showed the highest anti-inflammatory activity, while compound **14** was the least active one.

**Table 2:** The anti-inflammatory activity (% inhibition of edema thickness) and ulcer indices of compounds **12**–**19**.

Compd.	% Inhibition in Edema Thickness			Potency ^a^	Ulcerogenicity	
	1 h	2 h	3 h		UI ^b^	% Protection ^c^
Control	-	-	-	0	0	100
**12**	21.20	37.40	44.79	1.10	4.66	66.38
**13**	16.13	35.43	52.31	1.28	4.41	68.18
**14**	8.30	9.84	16.37	0.40	4.66	66.38
**15**	10.60	16.54	24.91	0.61	2.68	80.66
**16**	29.95	37.80	50.58	1.24	2.26	83.69
**17**	27.19	40.55	49.47	1.21	2.26	83.69
**18**	6.91	17.72	22.42	0.55	4.41	68.18
**19**	13.82	22.44	38.79	0.95	0	100
**Ibuprofen**	25.81	33.46	40.82	1.00	13.86	0

Anti-inflammatory activity (% inhibition of edema thickness) = (1 − L_t_/L_c_) × 100; L_t_ is the mean increase in paw thickness in rats treated with the tested compounds; L_c_ is the mean increase in paw thickness in control group; ^a^ Potency = anti-inflammatory activity of tested compound/anti-inflammatory activity of ibuprofen after 3 h of induction of inflammation; ^b^ Ulcer index (UI) = sum of (% incidence/10), average number of ulcers and average severity; *n* = 6; **^c^** percentage protection of ulcer (% inhibition of ulcer relative to ibuprofen) = (UI_St_ – UI_Test_/UI_St_) × 100.

**Figure 5:**
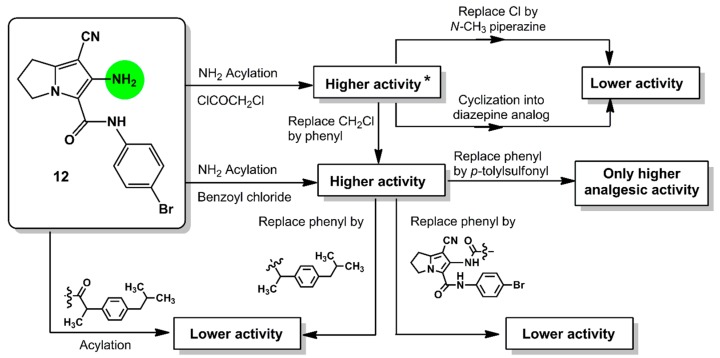
Relationship between the *in vivo* anti-inflammatory (at 3 h) and analgesic (at 2 h) activities of the new compounds **12**–**19** at a dose of 0.48 mmol/kg with the modifications in the chemical structure; * activity means both anti-inflammatory and analgesic activities.

Comparing the activity of compounds **13**–**18** with that of the parent compound **12**, it was conceptualized that the acylation of the amino group at C6 with aromatic/electron withdrawing groups such as 2-chloroacetyl (**13**), benzoyl (**16**), 4-tolylsulfonyl (**17**), resulted in an observed improvement of the anti-inflammatory activity. On the other hand, the replacement of the chloro atom in compound **13** with a 4-methylpiperazinyl moiety or the cyclization of the side chain as in diazepine derivative **15** decreases the inflammatory activity (Figure 5).

##### Analgesic Activity

**Figure 6:**
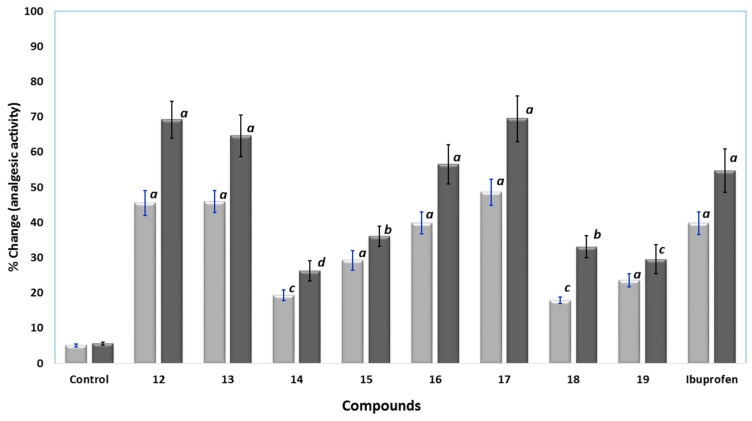
The analgesic activity results using hot-plate test for control, compound **12**–**19**, and ibuprofen at 0.24 (${}^{▄}$) and 0.48 mmol/kg (${}^{▄}$); data were represented as means ± SEM, *n* = 6; data were analyzed by One way ANOVA followed by student-Newman-Keuls multiple comparison test; % change = 100 × (T_1_ − T_0_)/T_0_; ***^a^*** statistically significant from control (*p* < 0.001); ***^b^*** statistically significant from control (*p* < 0.01); ***^c^*** statistically significant from control (*p* < 0.05); ***^d^*** statistically not significant from control.

The analgesic activities of the novel compounds **12**–**19** were determined in rats using the well-known hot plate method. Ibuprofen and the tested compounds **12**–**19** were injected intraperitoneally at a dose of at 0.24 mmol/kg and 0.48 mmol/kg. A hot-plate was used to induce thermal pain and the cutoff time was fixed to 15 s [30]. The analgesic responses were calculated as percent changes and results were presented in Figure 6. Compounds **12**, **13**, **16** and **17** displayed higher analgesic activity than ibuprofen, with analgesic potencies in the range of 1.03 to 1.27 times higher than ibuprofen at a dose of 0.48 mmol/kg. Compound **17** was the most active, at a dose of 0.48 mmol/kg. Moreover, compound **16** showed good activity comparable to that of ibuprofen. On the other hand, the analgesic activities of compounds **14**, **15**, **18** and **19** were lower than that of ibuprofen, where compound **14** was the least active, at 0.48 mmol/kg dose.

##### Acute Ulcerogenicity Studies

GIT toxicity is the main serious side effect of the NSAIDs. In this work, the ulcerogenicity of the novel compounds **12**–**19** was evaluated according to the previous reported methodology [31,32]. The tested compounds **12**–**19** and ibuprofen were given orally in a dose of 0.48 mmol/kg. The results revealed that all the new compounds were safer than ibuprofen with protection percentages in the range of 66.38%–100%. The hybrid **19** showed zero ulcer index. The data including ulcer index and protection percentages were calculated and the results were presented in Table 2.

##### Histopathological Studies

**Figure 7:**
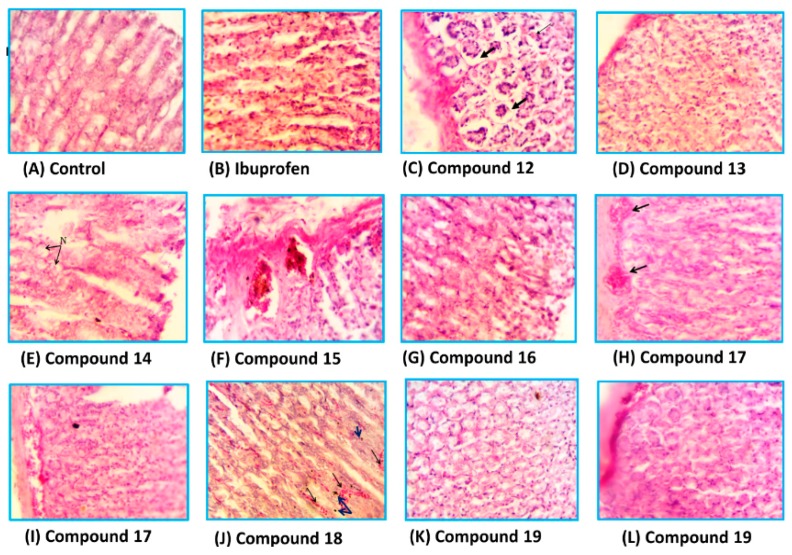
The histological TSs (**C**–**L**) in the stomach of rat treated with compounds **12**–**19**, respectively in comparison to control (**A**) and ibuprofen (**B**), using haematoxylin and eosin stain, 400×.

The stomachs of the rats used in ulcerogenicity test were subjected to a histopathological study to visualize the deep effects of ibuprofen and tested compounds on the stomach mucosa, submucosa and mucosal glands. The specimens were stained with haematoxylin and eosin stain [33]. Representative transverse sections (TSs) of the rat stomach treated with the new compounds are presented in Figure 7. It was found that the TSs in the stomach wall of the rats of the control group showed no histopathological effect and normal mucosal glands, while the TSs in the stomach of the rats treated with ibuprofen showed severe damage represented by leukocytes infiltration and necrosis in the mucosa layer with hypertrophy in mucosal glands. The TSs in stomachs treated with compound **12**, **13** and **14** showed variable degrees of necrosis with some leukocytes infiltration, while those treated with compounds **15**, **17** and **18** exhibited little histopathological effects such as congested blood vessels and variable degrees of leukocytes infiltration. Higher safety profiles were observed with compounds **16** and **19**, where the TSs showed normal mucosal glands with no histopathological effects.

### 2.3. Molecular Docking Study

#### 2.3.1. Docking Study into COX-1 Enzyme

**Figure 8:**
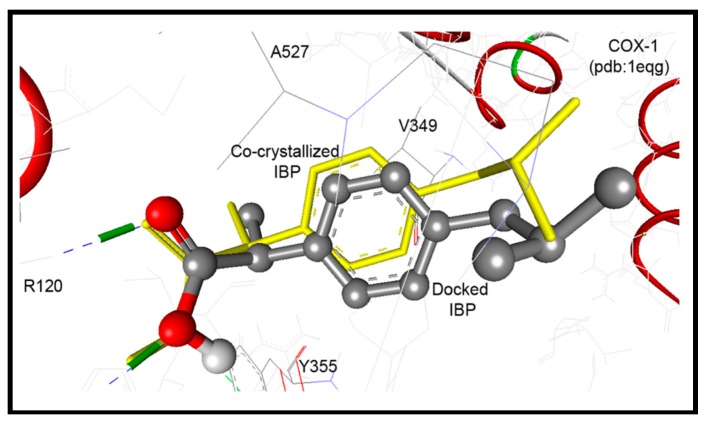
Ibuprofen docked superimposed onto the position of co-crystalized ligand within RMSD of 0.62 Å, and exhibited two hydrogen bonds between its COOH group and NH_2_ of Arg120.

The co-crystallized ibuprofen was used as a reference drug for the docking study of the newly synthesized pyrrolizines into the ovine COX-1 (pdb code: 1EQG) [34]. The binding site of COX-1 composed of the following amino acids: His90, Arg120, Val349, Leu352, Ser353, Tyr355, Arg513, Ala516, Phe518, Gly526, and Ala527. The bound ibuprofen was docked into COX-1 to validate the performance of AutoDock program in comparison to the biological experiment. It was docked superimposed onto the position of co-crystalized ligand within root mean square deviation (RMSD) of 0.62 Å, and it exhibited two hydrogen bonds between its COOH group and NH_2_ of Arg120 (Figure 8).

In accordance to this successful performance of the AutoDock program, docking of compounds **12**–**18** were done into COX-1 enzymes. They were docked within RMSD of 2.27 to 4.47 Å from the co-crystalized ibuprofen, and up to three hydrogen bonds were detected with the key amino acids Arg120 and Tyr355. Compounds **17** and **19** exhibited the highest binding affinities into COX-1 enzyme with binding free energies of −10.31 and −9.71 Kcal/mol, respectively (Table 3). Both of them exhibited one hydrogen bond between their pyrrolizin-2-sulfonamide and pyrrolizin-2-carboxamide and phenolic OH of Tyr355 and NH_2_ of Arg120, respectively. On the other hand, the cyclized diazepine **15** exhibited less binding affinity at −6.65 Kcal/mol (Table 3).

**Table 3:** Results of the flexible docking of compounds **12**–**19** into ovine COX-1 (pdb: 1eqg) [34] in comparison to the native co-crystallized ibuprofen.

Compd.	Δ*G*_b_^a^ (kcal/mol)	*K*_i_^b^	Hydrogen Bonds between Atoms of Compounds and Amino Acids of COX-1		RMSD ^c^ (Å)
			Atom of Compd.	Amino Acid	
**12**	−9.00	250.88 nM	5-Ph-NH	OH of Tyr355	2.92
			5-Ph-NHC=O	HN of Arg120	
**13**	−9.34	142.76 nM	7-CN	HN of Arg120	3.41
**14**	−6.41	20.18 µM	6-NH	OH of Tyr355	1.99
**15**	−6.65	13.26 µM	10-CN	HO of Tyr385	0.83
			2-C=O	HO of Ser530	
			2-C=O	HN of Leu531	
**16**	−8.14	1.08 µM	5-Ph-CONH	OH of Tyr355	2.27
**17**	−10.31	27.77 nM	6-NHS=O	HO of Tyr355	2.29
**18**	−0.68	316.74 mM	5-Ph-NHC=O	HN of Arg120	2.62
**19**	−9.71	76.20 nM	6-NHC=O	HN of Arg120	3.49
**Ibuprofen**	−9.40	128.86 nM	COO	H^1^N of Arg120	0.62
			COOH	H^2^N of Arg120	

**^a^** Binding free energy; **^b^** Inhibition constant; **^c^** Root mean square deviation.

#### 2.3.2. Docking Study into COX-2 Enzyme

**Figure 9:**
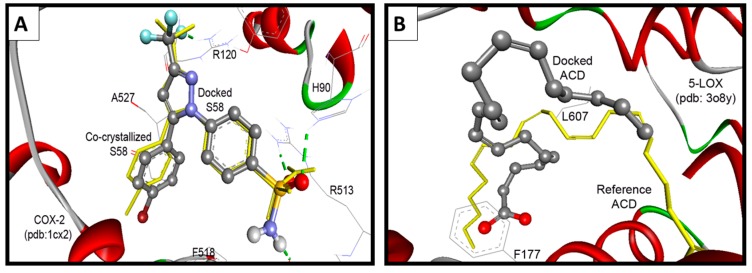
(**A**) Validation of the performance of AutoDock program by docking of the native co-crystallized ligands S58 into COX-2; (**B**) Validation of the performance of AutoDock program by docking of arachidonic acid into 5-LOX.

The native co-crystallized 1-phenylsulfonamido-3-trifluoromethyl-5-(*p*-bromophenyl)pyrazole ligand (S58) was used to parameterize molecular docking study against COX-2 (pdb code: 1cx2) [35]. The key amino acids of the binding site include: His90, Arg120, Val349, Leu352, Ser353, Tyr355, Arg513, Ala516, Phe518, Gly526, and Ala527. The docking performance was initially evaluated by docking of the native S58 ligand, where it revealed RMSD of 0.38 Å and formed four hydrogen bonds with Hist90, Arg120, Arg513, and Phe518. Furthermore, S58 exhibited excellent binding affinity (Δ*G*_b_: −11.49 Kcal/mol) (Figure 9A).

**Figure 10:**
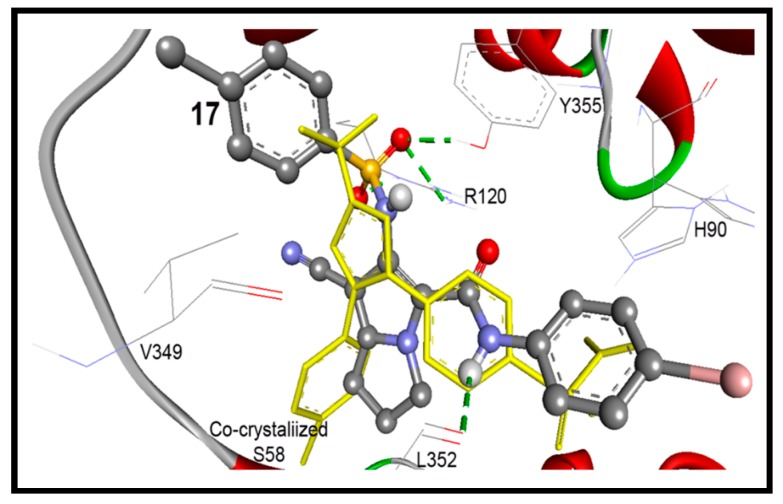
Docking mode of compound **17** (ball and stick) into COX-2 (pdb code: 1cx2). It revealed four hydrogen bonds (green dotted lines) with Arg120, Leu352, and Tyr355, within RMSD of 1.45 Å from the co-crystallized S58 ligand (yellow sticks).

These results revealed an extremely acceptable performance of the AutoDock program, which enables us to go further for investigation of the docking mode and the binding affinity of the newly synthesized compounds. Our compounds were docked within RMSD of 0.49 to 5.79 Å from the native co-crystalized ligand and revealed up to five hydrogen bonds. Compounds **17** and **18** exhibited the highest binding affinities into COX-2 enzyme of binding free energies of −11.63 and −10.93 Kcal/mol, respectively, Table 4. Compound **17** conserved four hydrogen bonds between its sulfonamide and aniline NH moieties and NH_2_ of Arg120, phenolic OH of Tyr355, and carbonyl group of Leu352, Figure 10.

On the other hand compound **18** showed two hydrogen bonds between its pyrrolizine-6-amide group and phenolic OH of Tyr355, and carbonyl group of Glu524. Whereas, the cyclized diazepine form of compound **15** {4-(4-bromophenyl)-2,5-dioxo-1,2,3,4,5,7,8,9-octahydro-[1,4]diazepino[5,6-*b*]pyrrolizine-10-carbonitrile} revealed poor binding affinity of −8.73 Kcal/mol (Table 4).

**Table 4:** Results of the flexible docking of compounds **12**–**19** into COX-2 (pdb: 1cx2) [35] in comparison to the native co-crystallized S58 ligand.

Compd.	Δ*G*_b_^a^ (kcal/mol)	*K*_i_^b^	Hydrogen Bonds between Atoms of Compounds and Amino Acids of COX-2		RMSD ^c^ (Å)
			Atom of Compd.	Amino Acid	
**12**	−8.79	362.12 nM	5-Ph-NH	O=C of Glu524	3.39
			5-Ph-NHC=O	H^1^N of Arg120	
			5-Ph-NHC=O	H^2^N of Arg120	
			5-NH	OH of Tyr355	
			7-CN	HN of His90	
**13**	−10.01	46.35 nM	5-NHC=O	H^1^N of Arg120	0.49
				H^2^N of Arg120	
**14**	−9.82	63.40 nM	-- ^d^		1.13
**15**	−8.73	400.2 nM	10-CN	HO of Ser530	1.78
**16**	−8.81	347.43 nM	6-Ph-C=O	HN of Arg120	1.13
**17**	−11.63	3.00 nM	5-Ph-NH	O=C of Leu352	1.45
			6-NHS=O^1^	H^1^N of Arg120	
			6-NHS=O^1^	H^2^N of Arg120	
			6-NHS=O^2^	HO of Tyr355	
**18**	−10.93	9.80 nM	6-NH	O=C of Glu524	5.79
			6-NHC=O	HO of Tyr355	
**19**	−10.27	29.51 nM	7-CN	HO of Ser530	1.59
**S58** **Ligand ^e^**	−11.49	3.81 nM	3-CF	HN of Arg120	0.38
			*p*-Ph-S=O^1^	HN of His90	
			*p*-Ph-S=O^2^	HN of Arg513	
			*p*-Ph-SONH	O=C of Phe518	

**^a^** Binding free energy; **^b^** Inhibition constant; **^c^** Root mean square deviation; **^d^** No hydrogen bond detected; **^e^** 1-Phenylsulfonamido-3-trifluoromethyl-5-(*p*-bromophenyl)pyrazole.

#### 2.3.3. Docking Study into 5-LOX Enzyme

Because there is no reference co-crystalized ligand for the 5-LOX (pdb code: 3O8Y) [36], molecular overlay superimposition was applied for human 5-LOX (pdb code: 3O8Y) and the mutated 15-lipoxygenase (pdb code: 3V99) [37]. The new center of the co-crystalized arachidonic acid (ACD) was utilized as a reference for docking of our compounds into 3O8Y, Figure 9B. Additionally, the two active site of 5-LOX (pdb code: 3V99) around the catalyst non-hem iron atom were used as the binding site. The first active site (AC1) has a three conserved histidines (His367, His372, and His550), as well as Asn554. The second active site (AC2) is composed of three key amino acids Phe177, Gln363, and Leu607. Compounds **16** and **17** revealed the highest binding affinities, means the lowest binding free energies of −11.06 and −10.84 kcal/mol, respectively. They formed (1-2) hydrogen bonds mainly with OH of Tyr181, and NH and CO Gln363 (Table 5).

**Figure 11:**
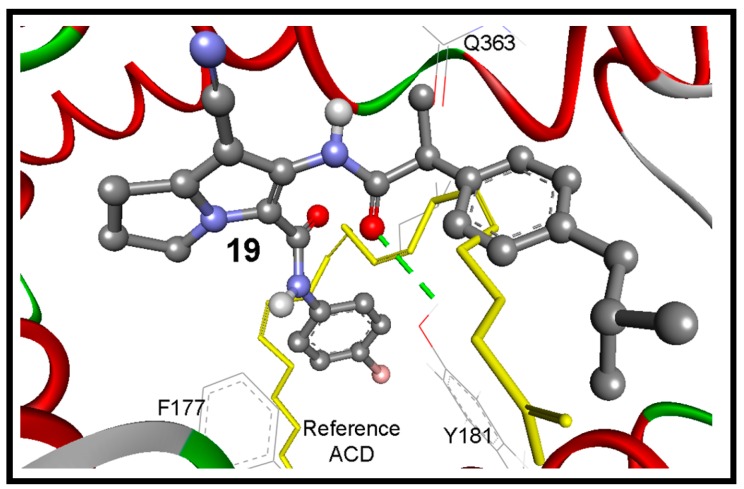
The binding mode of the interaction of compound **19** (ball and stick) into the target enzyme (5-LOX; pdb code: 3o8y). It bounds with Tyr181 by one hydrogen bond (green dotted lines) within RMSD of 3.24 Å from the reference ACD (yellow sticks).

Surprisingly the reference arachidonic acid (ACD) revealed a poorer binding affinity (−4.50 kcal/mol) than our compounds, where it interacts hydrophobically without any detected hydrogen bonds (Figure 11).

**Table 5:** Results of the flexible docking of compounds **12**–**19** into 5-LOX (pdb: 3o8y) [36] in comparison to the reference arachidonic acid (ACD).

Compd.	Δ*G*_b_^a^ (kcal/mol)	*K*_i_^b^	Hydrogen Bonds between Atoms of Compounds and Amino Acids of 5-LOX		RMSD ^c^ (Å)
			Atom of Compd.	Amino Acid	
**12**	−8.78	365.81 nM	5-Ph-NH	O=C of Gln363	5.80
			5-Ph-NHC=O	HO of Tyr181	
			7-CN	HN of Ala424	
**13**	−9.22	174.81 nM	5-Ph-NH	O=C of Gln363	5.42
			5-Ph-NHC=O	HO of Tyr181	
			7-CN	HN of Asn425	
**14**	−7.48	3.31 µM	6-NH	OH of Tyr181	2.73
**15**	−7.89	1.66 µM	2-C=O	HO of Thr364	5.86
			5-C=O	HN of His367	
**16**	−11.06	7.78 nM	5-Ph-NHC=O	HN of Gln363	2.68
			6-NH	OH of Tyr181	
**17**	−10.84	11.26 nM	6-NHS=O^1^	HN of Gln363	3.55
			6--NHS=O^2^	HN of His367	
**18**	+6.69	-- ^d^	5-Ph-NH	O=C of Ile673	4.25
			7-CN	HN of Gln363	
**19**	−12.16	1.23 nM	6-NHC=O	HO of Tyr181	3.24
**ACD Ligand ^e^**	−4.50	502.87 µM	-- ^d^		2.27

**^a^** Binding free energy; **^b^** Inhibition constant; **^c^** Root mean square deviation; **^d^** No hydrogen bond detected; ^e^ ACD—Arachidonic acid.

**Figure 12:**
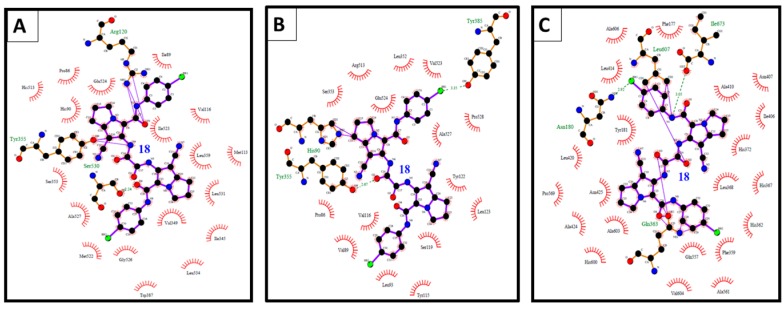
(**A**) The binding interactions of compound **18** into COX-1; (**B**) binding interactions of compound **18** into COX-2; and (**C**) binding interactions of compound **18** into 5-LOX illustrated by Ligplot.

Compound **18** revealed high binding energy (*i.e.*, low affinity) into COX-1 (ΔGb: −0.68 kcal/mol) and 5-LOX (ΔGb: +6.69 kcal/mol) (Figure 12A,C). On the other hand, it revealed a remarkable selective binding affinity into COX-2 better than its affinity into COX-1 and 5-LOX as clearly noticed in Table 3, Table 4 and Table 5. Therefore, it hold the lowest binding free energy into COX-2 (ΔGb: −10.93 kcal/mol) and two hydrogen bonds with Tyr355 and Glu525 (Figure 12B). The selective affinity of compound **18** into COX-2 over COX-1 and 5-LOX, may be attributed to its inferior stability into the latter targets by the external bonds (shown in purple lines) with the surrounding amino acids, namely: Arg120 and Tyr355 of COX-1 (Figure 12A), and Gln363, Leu607, and Ile673 of 5-LOX (Figure 12C). This steric hindrance was averted in the binding mode of compound **18** into COX-2 (Figure 12B).

## 3. Experimental Section

### 3.1. General Information

Chemical reagents and solvents were obtained from Sigma-Aldrich (Bayouni Company, Jeddah, Saudi Arabia). Solvents are dried by standard methods when necessary. Melting points (m.p.) were uncorrected and were carried out by open capillary tube method using an IA 9100MK-Digital Melting Point Apparatus. Microanalyses were carried out at the Microanalytical Center, Faculty of Science, Cairo University. Infrared spectra (IR) were recorded using a TENSOR 37 spectrophotometer (Bruker, Billerica, MA, USA, ATR accessory) and absorption were expressed in wave number (cm^−1^) using KBr disc. The proton magnetic resonance ^1^H-NMR spectra were recorded in the specified solvent at 500 MHz on a Bruker AVANCE II spectrometer, chemical shifts were reported on the δ scale and were related to that of the solvent and *J* values are given in Hz. ^13^C-NMR and DEPT135 spectra were obtained at 125 MHz on the same spectrometer. Mass spectra were recorded on a GCMS QP5050A spectrometer (Shimadzu, Europe) at 70 eV (EI). Thin layer chromatography, was done using Alugram Sil G/UV254 silica gel plates (Macherey-Nagel, Duren, Germany) and benzene-ethanol (9.5:0.5) as the eluting system. Compounds **9** [23], **11** [24] and **12** [25] were prepared according to the previously reported procedures.

### 3.2. Chemistry

*6-Amino-N-(4-bromophenyl)-7-cyano-2,3-dihydro-1H-pyrrolizine-5-carboxamide* (**12**): A mixture of *N*-(4-bromophenyl)-2-chloroacetamide (**11**, 1.86 g, 7.5 mmol), 2-(pyrrolidin-2-ylidine)malononitrile (**9**, 1 g, 7.5 mmol), and anhydrous potassium carbonate (1.04 g, 7.5 mmol) in dry acetone (50 mL) was stirred for 24 h under reflux. The reaction mixture was filtered while hot, concentrated and left aside. The crystals that separated were collected, dried and recrystallized from an ethanol-acetone mixture to give pale yellow crystals of the title compound, m.p. 228–230 °C, yield 68%, IR υ_max_/cm^−1^ 3343, 3275 (NHs), 3068 (C-H aromatic), 2970 (C-H aliphatic), 2218 (CN), 1703, 1652 (C=O). ^1^H-NMR (CDCl_3_, 500 MHz, δ ppm): 2.55 (m, 2H, CH_2_-2), 3.00 (t, 2H, *J* = 7.5 Hz, CH_2_-1), 3.57 (s, 2H, NH_2_), 4.41 (t, 2H, *J* = 7.5 Hz, CH_2_-3), 7.46 (d, 2H, *J* = 10 Hz, aromatic CH-3′, CH-5′), 7.55 (d, 2H, *J* = 8.7 Hz, aromatic CH-2′, CH-6′), 9.64 (s, H, NH). ^13^C-NMR (CDCl_3_, 125 MHz, δ ppm): 24.80, 25.44, 49.71, 83.48, 114.13, 114.66, 116.37, 121.24, 132.00, 137.24, 137.46, 145.24, 158.43. MS (EI): *m*/*z* (%) 346 (M^+^ + 2, 1), 344 (M^+^, 1); 298 (85), 262 (3), 235 (46), 219 (100), 207 (13), 191 (32), 174 (27), 165 (25), 146 (7), 109 (11), 95 (15), 77 (35). Anal. Calcd. for C_15_H_13_BrN_4_O (345.19): C, 52.19; H, 3.80; N, 16.23. Found: C, 51.81; H, 4.23; N, 16.35.

*N-(4-Bromophenyl)-6-(2-chloroacetamido)-7-cyano-2,3-dihydro-1H-pyrrolizine-5-carboxamide* (**13**): A mixture of 6-amino-*N*-(4-bromophenyl)-7-cycno-2,3-dihydro-1*H*-pyrrolizine-5-carboxamide (**12**, 1 g, 2.9 mmol) and chloroacetyl chloride (5.8 mmol) in dry benzene (20 mL) was stirred for 2 h and left aside to stand for 48 h at room temperature. The formed precipitate was filtered, washed with water and hot ethanol–acetone mixture. Compound **13** was obtained as white crystals, m.p. 300–302 °C, yield 71%, IR υ_max_/cm^−1^ 3269, 3236 (NHs), 3060 (C-H aromatic), 2996 (C-H aliphatic), 2226 (CN), 1674, 1651 (COs). ^1^H-NMR (DMSO-*d*_6_, 500 MHz, δ ppm): 2.45 (m, 2H, CH_2_-2), 3.00 (t, 2H, *J* = 7.5 Hz, CH_2_-1), 4.27 (t, 2H, *J* = 7.5 Hz, CH_2_-3), 4.33 (s, 2H, CH_2_-Cl), 7.52 (d, 2H, *J* = 10 Hz, aromatic CH-3′, CH-5′), 7.63 (d, 2H, *J* = 8.7 Hz, aromatic CH-2′, CH-6′), 9.59 (s, H, NH), 10.28 (s, H, NH). ^13^C-NMR (DMSO-*d*_6_, 125 MHz, δ ppm): 23.75, 24.59, 42.15, 48.72, 83.50, 113.77, 114.80, 116.79, 121.32, 125.57, 130.89, 137.27, 145.62, 156.81, 165.48. MS (EI): *m*/*z* (%) 420 (M^+^, 1), 387 (4), 372 (4), 351 (6), 322 (8), 294 (5), 267 (16), 252 (31), 236 (16), 228 (25), 220 (32), 195 (100), 186 (55), 174 (42), 168 (35), 155 (42), 146 (20), 134 (93), 126 (26), 111 (34), 94 (18), 77 (9). Anal. Calcd. for C_17_H_14_BrClN_4_O_2_ (421.68): C, 48.42; H, 3.35; N, 13.29. Found: C, 47.96; H, 3.70; N, 13.48.

*N-(4-Bromophenyl)-7-cyano-6-(2-(4-methylpiperazin-1-yl)acetamido)-2,3-dihydro-1H-pyrrolizine-5-carbox-amide* (**14**): A mixture of *N*-(4-bromophenyl)-6-(2-chloroacetamido)-7-cycno-2,3-dihydro-1*H*-pyrrolizine-5-carboxamide (**13**, 3.8 mmol), *N*-methylpiperazine (0.76 g, 7.6 mmol) and anhydrous potassium carbonate (1.04 g, 7.5 mmol) in absolute ethanol (20 mL) was refluxed for 6 h. The separated product was filtered, washed with water and recrystallized from ethanol–acetone mixture to give white crystals, m.p. 220–223 °C, yield 65%, IR υ_max_/cm^−1^ 3215 (NHs), 3057 (C-H aromatic), 2940, 2823 (C-H aliphatic), 2225 (CN), 1671, 1651 (COs). ^1^H-NMR (CDCl_3_, 500 MHz, δ ppm): 2.34 (s, 3H, CH_3_), 2.57 (m, 6H, CH_2_-2, CH_2_-3″, CH_2_-5″), 2.75 (m, 4H, CH_2_-2″, CH_2_-6″), 3.05 (t, 2H, *J* = 7.5 Hz, CH_2_-1), 3.26 (s, 2H, *J* = 7.5, COCH_2_), 4.40 (t, 2H, *J* = 7.5 Hz, CH_2_-3), 7.45 (d, 2H, *J* = 10 Hz, aromatic CH-3′, CH-5′), 7.51 (d, 2H, *J* = 10 Hz, aromatic CH-2′, CH-6′), 9.42 (s, H, NH), 10.04 (s, H, NH). ^13^C-NMR (CDCl_3_, 125 MHz, δ ppm): 25.02, 25.62, 45.96, 49.33, 53.73, 55.20, 61.16, 83.89, 113.90, 116.71, 120.20, 121.09, 124.31, 131.98, 137.41, 145.66, 157.48, 172.67. MS (EI): *m*/*z* (%) 469 (M^+^ − 15, 1), 421 (4), 401 (2), 376 (9), 362 (18), 308 (21), 286 (5), 262 (10), 242 (5), 222 (8), 199 (13), 174 (14), 156 (65), 140 (21), 112 (35), 97 (32), 82 (19), 78 (5), 40 (100). Anal. Calcd. for C_22_H_25_BrN_6_O_2_ (485.38): C, 54.44; H, 5.19; N, 17.31. Found: C, 54.67; H, 4.85; N, 17.24.

*4-(4-Bromophenyl)-2,5-dioxo-1,2,3,4,5,7,8,9-octahydro-[1,4]diazepino[5,6-b]pyrrolizine-10-carbonitrile* (**15**): A mixture of **13** (0.76 g, 1.8 mmol) and anhydrous potassium carbonate (0.25 g, 1.8 mmol) in dry DMF (20 mL) was stirred at room temperature for 24 h. The reaction mixture was poured onto ice-cooled water. The formed precipitate was filtered off, washed with water, dried and crystallized from ethanol–acetone mixture to give compound **15b** as white crystals, m.p. 256–258 °C, yield 61%, IR υ_max_/cm^−1^ 3314, 3238 (NHs), 3068 (C-H aromatic), 2995 (C-H aliphatic), 2216 (CN), 1704, (C=O), 1588, 1545, 1487, 1312 (C-N, N-H, C-O). ^1^H-NMR (DMSO-*d*_6_, 500 MHz, δ ppm): 2.01 (m, 2H, CH_2_-2), 3.00 (t, 2H, *J* = 7.8 Hz, CH_2_-1), 3.76 (t, 2H, *J* = 7.2 Hz, CH_2_-3), 4.57 (s, 2H, COCH_2_), 7.50–7.54 (m, 4H, aromatic protons), 10.37 (s, H, NH). ^13^C-NMR (DMSO-*d*_6_, 125 MHz, δ ppm): 19.72, 36.40, 43.57, 50.67, 59.59, 115.73, 116.30, 118.04, 121.74, 132.16, 134.01, 138.43, 164.86, 172.64. MS (EI): *m*/*z* (%) 384 (M^+^, 2), 357 (100), 328 (2), 276 (6), 198 (3), 171 (4), 152 (34), 138 (3), 124 (6), 104 (4), 92 (4), 77 (5). Anal. Calcd. for C_17_H_13_BrN_4_O_2_ (385.21): C, 53.00; H, 3.40; N, 14.54. Found: C, 52.71; H, 3.42; N, 14.24.

*6-Benzamido-N-(4-bromophenyl)-7- cyano-2,3-dihydro-1H-pyrrolizine-5-carboxamide* (**16**): A mixture of **12** (1 g, 2.9 mmol) and benzoyl chloride (5.8 mmol) in dry benzene (30 mL) was stirred for 2 h and left to stand overnight at room temperature. The formed precipitate was filtered, washed with water and recrystallized from an ethanol–acetone mixture. Compound **16** was obtained as a white solid, m.p. 285–288 °C, yield 66%, IR υ_max_/cm^−1^ 3210 (NHs), 3041 (C-H aromatic), 2982 (C-H aliphatic), 2223 (CN), 1664, 1642 (COs). ^1^H-NMR (CDCl_3_, 500 MHz, δ ppm): 2.60 (m, 2H, CH_2_-2), 3.08 (t, 2H, *J* = 7.5 Hz, CH_2_-1), 4.44 (t, 2H, *J* = 7.5 Hz, CH_2_-3), 7.42–7.68 (m, 9H, aromatic protons), 8.04 (s, H, NH), 9.98 (s, H, NH). ^13^C-NMR (CDCl_3_, 125 MHz, δ ppm): 25.05, 25.67, 49.63, 84.56, 114.09, 116.81, 120.67, 121.10, 124.59, 127.66, 129.19, 131.99, 132.33, 133.28, 137.26, 145.78, 157.54, 169.18. MS (EI): *m*/*z* (%) 448 (M^+^), 352 (31), 324 (5), 311 (6), 291 (79), 263 (22), 252 (13), 195 (100), 186 (28), 174 (43), 171 (16); 161 (17), 155 (22); 146 (32), 126 (29), 112 (23), 92 (21), 77 (46). Anal. Calcd. for C_22_H_17_BrN_4_O_2_ (449.30): C, 58.81; H, 3.81; N, 12.47. Found: C, 59.22; H, 3.68; N, 12.41.

*N-(4-Bromophenyl)-7-cyano-6-(4-methylphenylsulfonamido)-2,3-dihydro-1H-pyrrolizine-5-carboxamide* (**17**): A mixture of compound **12** (1 g, 2.9 mmol) and 4-toluenesulfonyl chloride (2.9 mmol), anhydrous potassium carbonate (0.5 g, 3.65 mmol) in dry acetone (30 mL) was stirred under reflux for 4 h and left to stand, filtered, and the solvent was evaporated to dryness under reduced pressure. The formed precipitate was washed with water and recrystallized from ethanol–acetone mixture. Compound **17** was obtained as white crystals, m.p. 272–274 °C, yield 74%, IR υ_max_/cm^−1^ 3358, 3272 (NHs), 3063, 3033 (C-H aromatic), 2919, 2800 (C-H aliphatic), 2222 (CN), 1668 (COs), 1595 (C=C), 1310, 1291 (SO_2_N). ^1^H-NMR (CDCl_3_, 500 MHz, δ ppm): 2.49 (s, 3H, CH_3_), 2.53 (m, 2H, CH_2_-2), 2.93 (t, 2H, *J* = 7.5 Hz, CH_2_-1), 4.48 (t, 2H, *J* = 7.5 Hz, CH_2_-3), 6.57 (broad s, H, SO_2_NH), 7.37 (d, 2H, *J* = 7.8 Hz, aromatic protons), 7.48 (d, 2H, *J* = 8.0 Hz, aromatic protons), 7.61 (d, 2H, *J* = 8.1 Hz, aromatic protons), 7.74 (d, 2H, *J* = 7.8 Hz, aromatic protons), 9.75 (s, H, CONH). ^13^C-NMR (CDCl_3_, 125 MHz, δ ppm): 21.77, 24.82, 25.35, 50.30, 86.22, 112.64, 117.03, 121.66, 121.99, 123.00, 128.22, 130.22, 131.97, 133.98, 137.00, 145.59, 145.74, 156.89. MS (EI): *m*/*z* (%) 501 (M^+^ + 3, 1), 500 (M^+^ + 2, 6), 499 (M^+^ + 1, 2), 498 (M^+^, 6), 343 (9), 328 (25), 315 (100), 301 (19), 264 (43), 235 (15), 208 (7), 174 (9), 155 (4), 146 (10), 117 (15), 91 (38), 65 (21). Anal. Calcd. for C_22_H_19_BrN_4_O_3_S (499.38): C, 52.91; H, 3.83; N, 11.22. Found: C, 53.28; H, 4.03; N, 11.42.

*N^1^,N^2^-Bis(5-((4-Bromophenyl)carbamoyl)-7-cycno-2,3-dihydro-1H-pyrrolizin-6-yl)oxalamide* (**18**): A mixture of compound **12** (1 g, 2.9 mmol) and oxalyl chloride (5.8 mmol) in dry benzene (20 mL) was stirred for 2 h. The reaction mixture was left aside overnight at room temperature. The formed precipitate was filtered off, washed with two portions of water, dried and washed with hot acetone to give compound **18** as white crystals, m.p. 318–321 °C, yield 63%, IR υ_max_/cm^−1^ 3274 (NHs), 3066 (C-H aromatic), 2989, 2852 (C-H aliphatic), 2231 (CN), 1746, 1700, 1660 (COs). ^1^H-NMR (DMSO-*d*_6_, 500 MHz, δ ppm): 2.47 (m, 4H, CH_2_-2 + CH_2_-2′), 3.01 (t, 4H, *J* = 7.5 Hz, CH_2_-1 + CH_2_-1′), 4.28 (t, 2H, *J* = 7.5 Hz, CH_2_-3 + CH_2_-3′), 7.53 (d, 4H, *J* = 10 Hz, aromatic CH-3 + CH-3 and CH-5 + CH-5′), 7.60 (d, 4H, *J* = 10 Hz, aromatic CH-2 + CH-2′ and CH-6 + CH-6′), 9.70 (s, 2H, two CONHPh), 10.72 (s, 2H, NHCOCONH). ^13^C-NMR (DMSO-*d*_6_, 125 MHz, δ ppm): 24.82, 25.72, 49.73. 56.50, 84.57, 114.97, 115.89, 117.73, 122.47, 131.98, 138.40, 146.77, 157.93, 161.72. MS (EI): *m*/*z* (%) 747 (M^+^ + 5, 1), 746 (M^+^ + 4, 1), 744 (M^+^ + 2, 2), 743 (M^+^ + 1, 1), 742 (M^+^, 3), 729 (4), 657 (2), 371 (3), 344 (17), 310 (4), 262 (72), 228 (36), 206 (4), 186 (10), 174 (37), 162 (32), 146 (100), 119 (13), 91 (15), 60 (26). Anal. Calcd. for C_32_H_24_Br_2_N_8_O_4_ (744.39): C, 51.63; H, 3.25; N, 15.05. Found: C, 52.08; H, 3.72; N, 15.34.

*(R,S)-N-(4-Bromophenyl)-7-cycno-6-(2-(4-isobutylphenyl)propanamido)-2,3-dihydro-1H-pyrrolizine-5-carboxamide* (**19**): A mixture of ibuprofen (1.5 g, 7.5 mmol) and thionyl chloride (2 mL) was heated on water bath for one hour. The reaction mixture was cooled and the excess thionyl chloride was removed under vacuum. The residue obtained was dissolved in 20 mL dry benzene and pyrrolizine-5-carboxamide **12** (1 g, 2.9 mmol) was added. The reaction mixture was stirred for two hours then left aside to stand for 48 h at room temperature. The formed precipitate was filtered, washed with water, and recrystallized from ethanol–acetone to give compound **19** as white crystals, m.p. 217–219 °C, yield 58%, IR υ_max_/cm^−1^ 3402, 3289 (NHs), 3060, 3018 (C-H aromatic), 2954, 2868 (C-H aliphatic), 2225 (CN), 1732, 1663 (COs). ^1^H-NMR (CDCl_3_, 500 MHz, δ ppm): 0.88 (d, 6H, *J* = 5 Hz, CH(CH_3_)_2_), 1.66 (d, 3H, *J* = 7 Hz, COCHCH_3_), 1.82 (m, H, CH(CH_3_)_2_), 4.43 (d, 2H, *J* = 5 Hz, Ph-CH_2_), 2.51 (m, 2H, CH_2_-2), 2.98 (t, 2H, *J* = 7.5 Hz, CH_2_-1),3.83 (q, H, *J* = 7 Hz, COCHCH_3_), 4.28–4.37 (m, 2H, CH_2_-3), 7.08 (d, 2H, *J* = 10 Hz, two aromatic CH), 7.23 (d, 3H, *J* = 10 Hz, two aromatic CH + CONH),7.34 (d, 2H, *J* = 10 Hz, two aromatic CH), 7.38 (d, 2H, *J* = 10 Hz, two aromatic CH), 9.54 (s, H, CONH). ^13^C-NMR (CDCl_3_, 125 MHz, δ ppm):δ 18.10, 22.34, 22.37, 24.49, 25.61, 30.13, 44.98, 46.97, 49.48, 84.40, 113.69, 116.75, 120.30, 121.14, 124.47, 127.41, 130.12, 131.88, 136.38, 141.78, 1455.63, 157.47, 177.14. MS (EI): *m*/*z* (%) 532 (M^+^, 1), 394 (2), 373 (2), 362 (14), 335 (10), 319 (2), 286 (35), 262 (8), 209 (30), 202 (33), 161 (100), 145 (17), 117 (8), 81 (10). Anal. Calcd. for C_28_H_29_BrN_4_O_2_ (533.46): C, 63.04; H, 5.48; N, 10.50. Found: C, 63.37; H, 5.20; N, 10.62.

### 3.3. Biological Evaluation

#### 3.3.1. *In Vitro* COX-1/2 Inhibitory Assay

The ability of the tested compound **12**–**18** to inhibit COX-1 (ovine) and COX-2 (human recombinant) was measured using COX colorimetric inhibitor screening assay kit provided by Cayman Chemical, Ann Arbor, MI, USA (Catalog No. 701050). The assay was carried out according to the manufacturer’s instructions and as described before [26,27,28].

#### 3.3.2. *In Vivo* Biological Evaluation

##### Animals

Adult albino rats of both sex weighing 100–140 g were used in evaluation of the anti-inflammatory, analgesic, ulcerogenic activity in addition to the histopathological study of the new pyrrolizines **12**–**19**. Animal ethical use clearance was obtained from the Ethical Committee at the College of Pharmacy, Umm Al-Qura University. Normalization of the animals with laboratory conditions were achieved by keeping them in laboratory one week before starting the experiments. Standard rat pellet diet was used for feeding animals and water *ad libitum*.

##### Anti-Inflammatory Activity

The anti-inflammatory activity of the novel compounds **12**–**19** was evaluated in comparison with ibuprofen as a standard, using a rat paw edema model as described by Winter *et al.* [29]. Adult albino rats of both sexes (100–140 gm) were used. Rats were uniformly hydrated by giving 3 mL water for each rat through gastric inoculation to reduce variability to edema response. Animals were then divided into groups of six animal each. The control group was given saline solution containing few drops of sodium carboxymethyl cellulose (CMC) 1% and DMSO. Ibuprofen (50 and 100 mg/kg) was given as standard drug. The new compounds **12**–**19** in a dose molecularly equivalent to ibuprofen were dissolved in 0.5 mL DMSO and CMC 1% was added dropwise with continuous triturating to complete the specified volume. The tested compounds were administered intraperitoneally (IP). The induction of inflammation was achieved by subcutaneous (SC) injection of carrageenan-sodium gel, into the sub-plantar region of the right hind paw. The dorsoventral diameter (thickness) of the right and left hind paw of each rat was measured using digital calipers with accuracy of 0.01 mm (Cole-Parmer, Vernon Hills, IL, USA). The thickness of edema was measured 1, 2 and 3 h after induction of inflammation. The left hind paw diameter served as a control for the degree of inflammation in the right hind paw. The changes in the edema thickness were expressed as mean ± SEM, Figure 4. The anti-inflammatory activity was calculated as % inhibition of inflammation = (1 − L_t_/L_c_) × 100. Where L_t_ is the mean increase in paw thickness in rats treated with the tested compounds and L_c_ is the mean increase in paw thickness in control group. Data were collected, and statistically analyzed by One way ANOVA followed by student-Newman-Keuls multiple comparison test, Table 2.

##### Analgesic Activity

The analgesic activity of the novel pyrrolizines were carried out using the thermal model (hot-plate test) according to a previous report [30]. An electronically controlled hot-plate (Harvard Apparatus Ltd., Kent, UK) was used and the cut-off time was 15 s and the temperature was adjusted to 52 ± 0.1 °C. Animals were then divided into groups of six animal each. Rats were assigned numbers of 1–6 in each group. The reference drug was given intraperitoneally at a dose of 50 mg/kg (0.24 mmol/kg) and 100 mg/kg (0.48 mmol/kg). The tested compounds **12**–**19** were given in a dose molecularly equivalent to ibuprofen. The basal time was measured for each rat just immediately after injecting the drugs (T_0_) and two hours after injecting the tested compounds (T_1_). The response latencies (time elapsed until licking paw or jumping) were measured and expressed as mean ± SEM. The percent changes (analgesic effects) of the tested compounds were calculated as percent change in latencies divided by baseline time (100 × (T_1_ − T_0_)/T_0_) and results were expressed as % change ± SEM, Figure 6.

##### Acute Ulcerogenicity Study

GIT toxicity was evaluated according to the previous report [31]. Adult albino rats of both sexes were divided into groups of six animals each. The test compounds were dissolved first in 0.5 mL DMSO, then CMC (1%) was added dropwise with trituration. Rats were fasted 20 h before drug administration. The eight tested compounds and ibuprofen were given orally in a dose of 0.24 and 0.48 mmol/kg/day suspended in 1% CMC, while the control group received vehicle (1% CMC containing few drops of DMSO). Rats were then fasted for 2 h, allowed to feed for 2 h then fasted again for another 20 h. Other doses were given in the second and third days. In the fourth day, Pentobarbital (60 mg/kg IP) was used to induce deep anesthesia then rats were sacrificed. The stomachs were removed, opened along with the greater curvature and rinsed with 0.9% saline. The number of mucosal damages (red spots) were counted using magnifying lens and their severity (ulcerogenic severity) was graded in a scale from 0 to 3 as follows: (0) normal (no injury), (0.5) red coloration, (1) spot ulcer, (1.5) hemorrhagic streaks, (2) ulcer > 3 mm, (3) ulcer > 5 mm. The ulcer index was calculated using the following equation ulcer index (UI) = UN + US + UP/10, where UN is the average number pf ulcers per animal, US is the average of severity score, UP is the percent of animal with ulcer [32]. UI and % protection were calculated for each compound and presented in Table 2.

##### Histopathological Study

The rats sacrificed three days post-treatment with ibuprofen and tested with compounds **12**–**19** in ulcerogenic studies were used in this study. The study investigated the deep effect of the tested compounds on the mucosa, submucosa and mucosal gland. The stomachs which were opened longitudinally along the greater curvature were used and the specimens were taken from the stomachs of all cases. These specimens were fixed in 10% formalin solution for 72 h. The samples were processed and embedded in paraffin wax. Microtomy was done and 5 microns tissue sections were obtained and mounted on clean glass slides. The later was stained with haematoxylin and eosin stain [33]. The results of the histopathological study were presented in Figure 7.

### 3.4. Molecular Docking Study

This comparative docking study was performed by docking our compounds **12**–**19** into the two COX isoforms: COX-1 and COX-2 in addition to 5-LOX and compared with the parent co-crystallized ligands. The main purpose of this study was to predict the binding mode and the binding affinity of the newly synthesized compounds with relevant amino acids in the binding sites of COX-1, COX-2, and 5-LOX enzymes.

#### 3.4.1. Preparation of the COX-1, COX-2, and 5-LOX Protein and Ligands (12–19)

The protein structures were handled by using Accelrys Discovery Studio Visualize v4.1 software (Accelrys Inc., San Diego, CA, USA (2005)). The crystal structures of proteins were retrieved from Protein Data Bank (http://www.rscb. org/pdb) in a pdb format. COX-1, COX-2, and 5-LOX (PDB code: 1EQG, 1CX2, and 3O8Y) of X-ray resolutions: 2.61, 3.0, and 2.39 Å, respectively) [34,35,36]. All water molecules were removed because the extra water molecules will mask the protein surface from the ligand. The three-dimensional structures (pdb format) of the ligands (**12**–**19**) were constructed using Chem3D Ultra 8.0 software, then energetically minimized by using MOPAC with 100 iterations and minimum RMS gradient of 0.10. The AutoDock Tool (ADT) automatically computes Gasteiger charges to the 3D structures of the ligands.

#### 3.4.2. Preparation of the Flexible Residue File

AutoDock 4.2 has the ability to manipulate the flexibility not only of the ligand but also for the flexible moieties of the protein structure during docking process. Therefore, we have to consider this advancement by selecting the key amino acid residues that are able to modulate their conformation during ligand interaction into the binding site. In this study, the flexible residues for COX-1 are Arg120 and Tyr355, for COX-2 are Arg120 and His90, and for 5-LOX are Ser171, Phe177, Leu607, and Gln363.

#### 3.4.3. Calculation of Affinity Maps by Using AutoGrid

AutoGrid calculates grid parameter files and generates maps for each type of atom within a given area. We used the 3D grid of 60 × 60 × 60 Å size (x, y, z) with a spacing of 0.375 Å centered at 26.643, 33.106, and 200.251 Å for docking into COX-1, at 23.947, 21.582, and 15.436 Å for docking into COX-2, and centered at −2.917, 19.760, and −1.690 Å for docking into 5-LOX. The co-crystallized ibuprofen was used to guide the docked inhibitors within COX-1 receptor while S58 native ligand was used within COX-2 receptor, whereas, the co-crystalized arachidonic acid (ACD) into the mutated 15-lipoxygenase (pdb code: 3V99) [37] was used to guide the docking into human 5-LOX (pdb code: 3O8Y) [36] because the human 5-LOX has no bound ligand. Therefore, molecular overlay superimposition was applied for human 5-LOX (3O8Y) and the mutated 15-lipoxygenase (3V99). The new center of the co-crystalized arachidonic acid (ACD) was utilized as a reference for the docking of our compounds into 3O8Y.

#### 3.4.4. Defining the Docking Parameters and Running the Docking Simulation

The default values of AutoDock4.2 program were set during the ten docking runs, and the Lamarckian Genetic Algorithm, which is the most recent docking algorithm, was involved. Ten conformations were obtained for the protein-ligand complex and the flexible residues (namely: Arg120 and Tyr355 for COX-1, Arg120 and His90 for COX-2, and Ser171, Phe177, Leu607, and Gln363 for 5-LOX) within the binding pocket. The docking clusters were scored and ranked descendingly by the program according their calculated binding free energies. The binding interactions were analyzed and images were generated using Accelrys Discovery Studio visualizer v4.1 software.

## 4. Conclusions

In summary, a series of novel 6-amino-*N*-(4-bromophenyl)-7-cyano-2,3-dihydro-1*H*-pyrrolizine-5-carboxamide **12** and its derivatives **13**–**19** was designed and synthesized. Some of these new compounds have displayed anti-inflammatory and analgesic activities higher than ibuprofen. The molecular mechanism underlying the anti-inflammatory activity of these derivatives was investigated via the ability of these compounds to inhibit COX-1/-2 enzymes. In addition, almost all the newly synthesized compounds showed a safer gastric profile than ibuprofen as indicated by their low ulcerogenic indices and the histopathological studies. The docking studies revealed that the new compounds have variable degrees of binding affinity for COX/5-LOX enzymes. This binding affinity was modulated with the change of the substituents on the 6-amino group of the pyrrolizine scaffold. The results obtained in this study warrant that the new pyrrolizine derivatives **12**–**19** represent a promising scaffold for further development into potent and safe anti-inflammatory/analgesic agents.

## Supplementary Materials

Supplementary materials including all spectral data and copies of IR, Mass, ^1^H-NMR and ^13^C-NMR spectra, of all final compounds (Figures S1–S35). Supplementary materials can be accessed at: http://www.mdpi.com/1420-3049/21/2/201/s1.
