# Supplementary material for: Design, Synthesis, and Biological Evaluation of Some Novel Pyrrolizine Derivatives as COX Inhibitors with Anti-Inflammatory/Analgesic Activities and Low Ulcerogenic Liability
Source: Molecules. 2016 Feb 8;21(2):201. doi: 10.3390/molecules21020201 (PMC6273963; doi:10.3390/molecules21020201)
Supplement: Supplementary file 1 [file molecules-21-00201-s001.pdf]

Supplementary Materials: Design, Synthesis, and Biological Evaluation of Some Novel Pyrrolizine Derivatives as COX Inhibitors with Anti-inflammatory/Analgesic Activities and Low Ulcerogenic Liability

Ahmed M. Gouda [1,2,*], Hamed I. Ali [1,3], Waleed H. Almalki [4], Mohamed A. Azim [1,5], Mohammed A. S. Abourehab [6] and Ahmed H. Abdelazeem [2]

IR Spectra

Infrared spectra (IR) were done using BRUKER TENSOR 37 spectrophotometer and absorption were expressed in wave number (cm-1) using KBr disc.

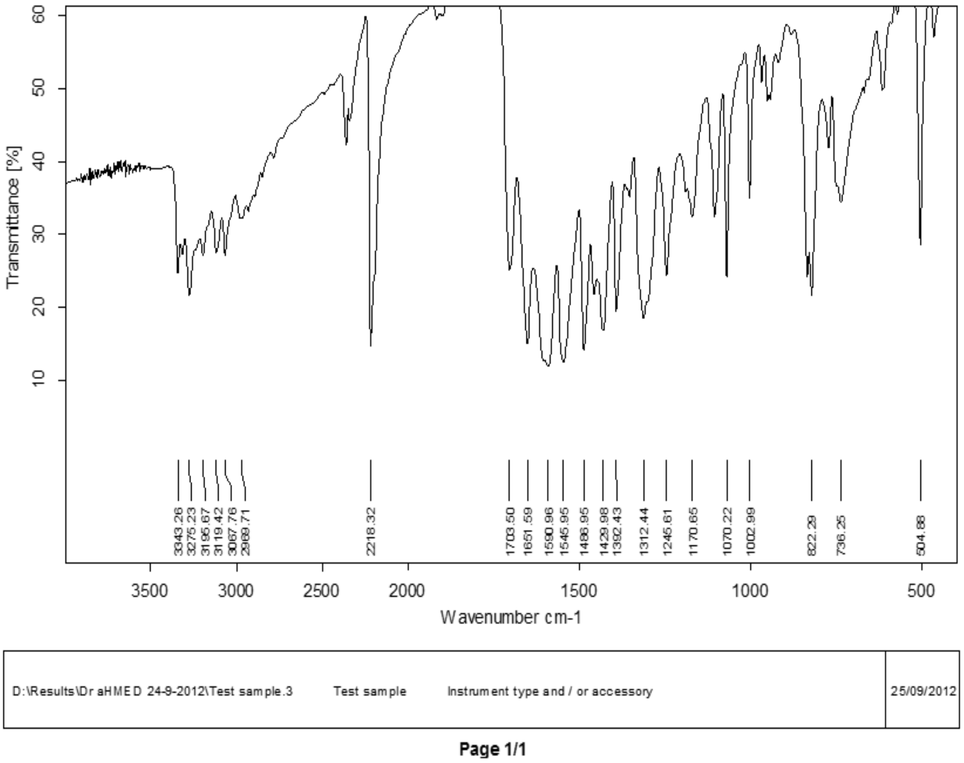

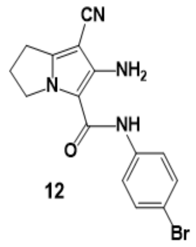

Figure S1: IR spectrum of compound 12

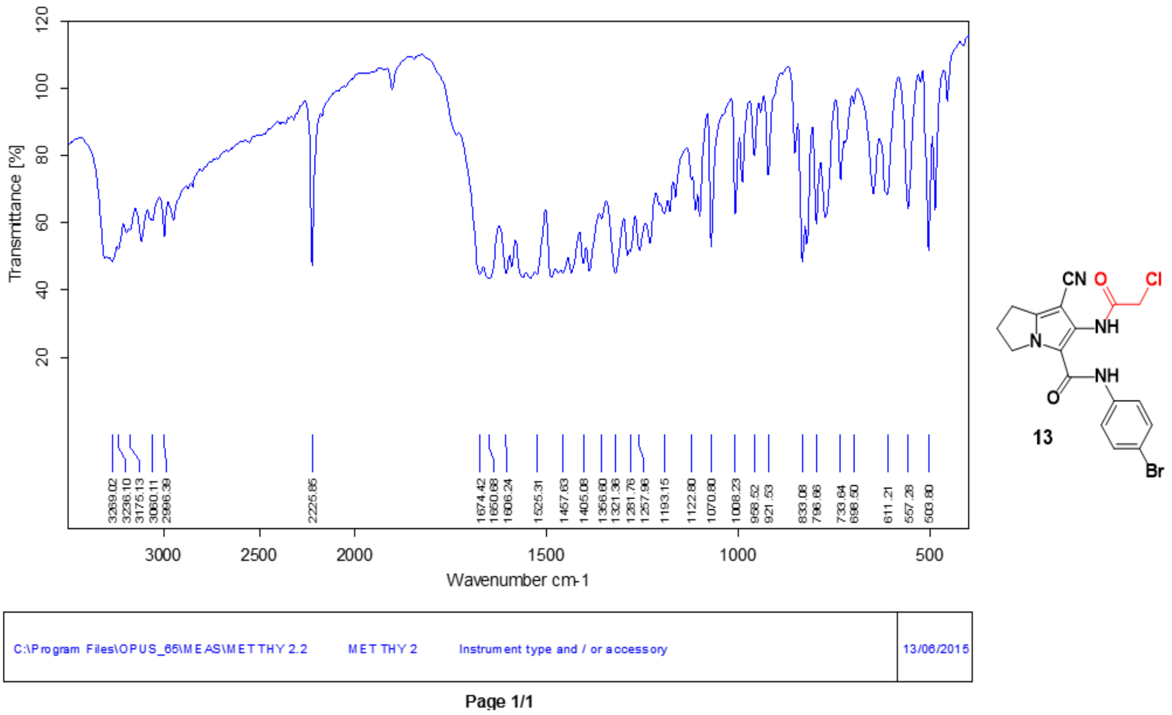

Figure S2: IR spectrum of compound 13

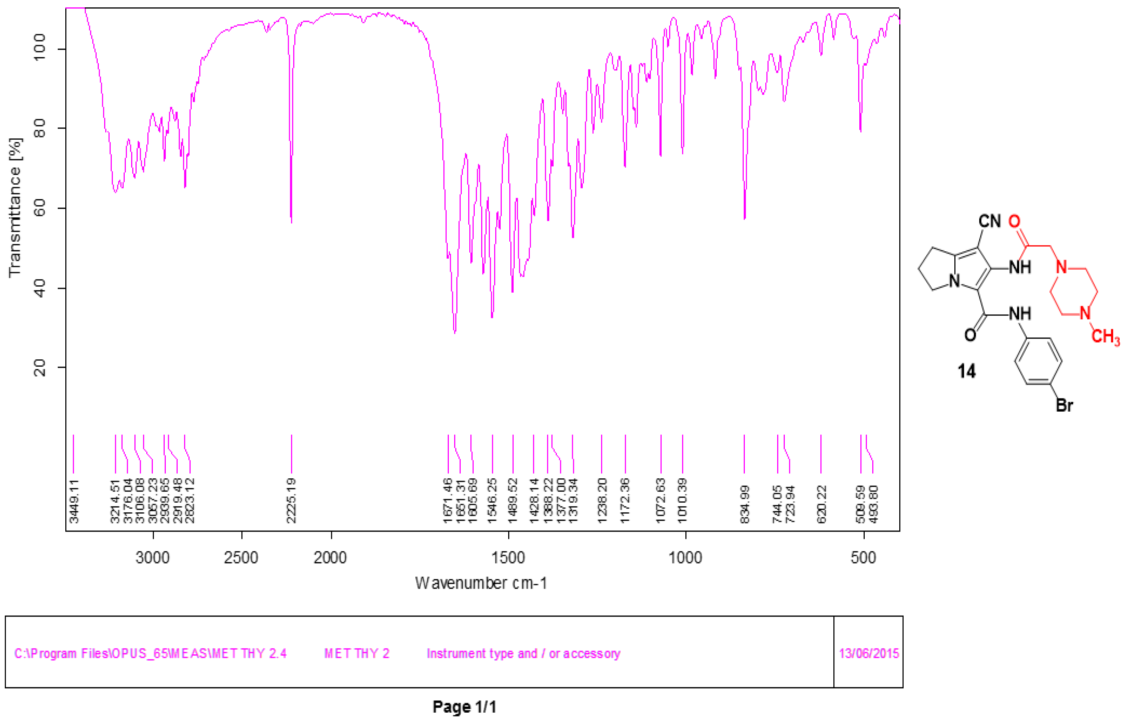

Figure S3: IR spectrum of compound 14

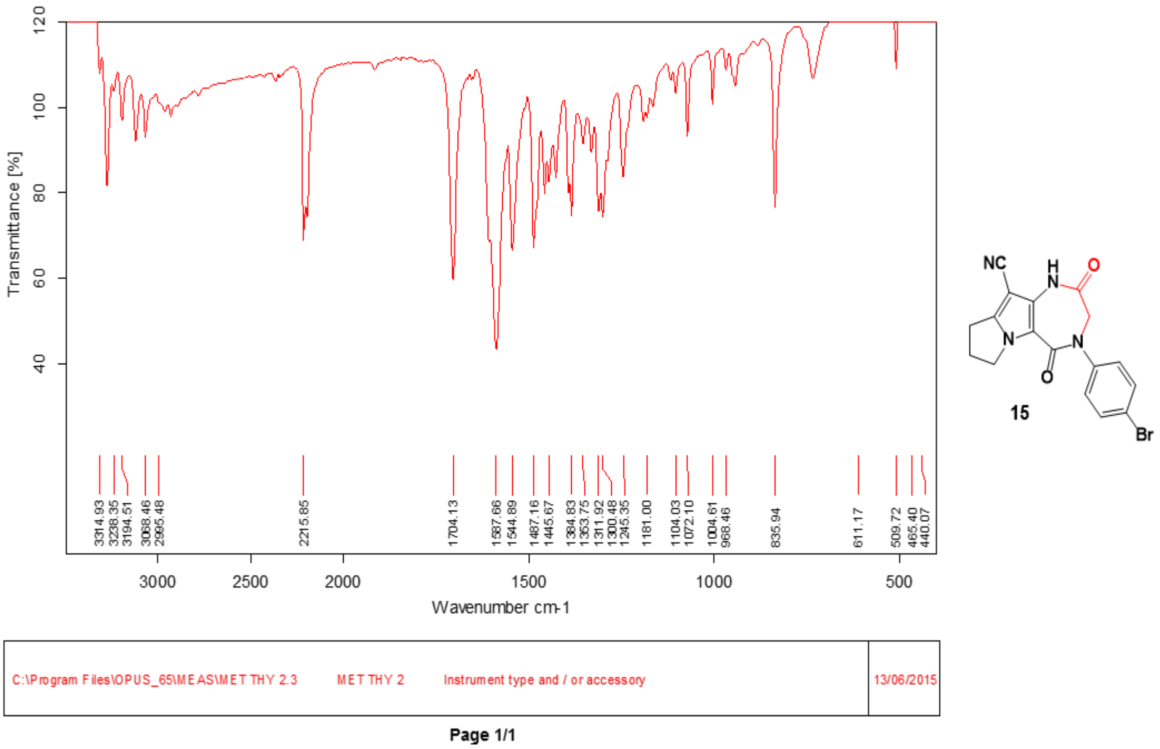

Figure S4: IR spectrum of compound 15

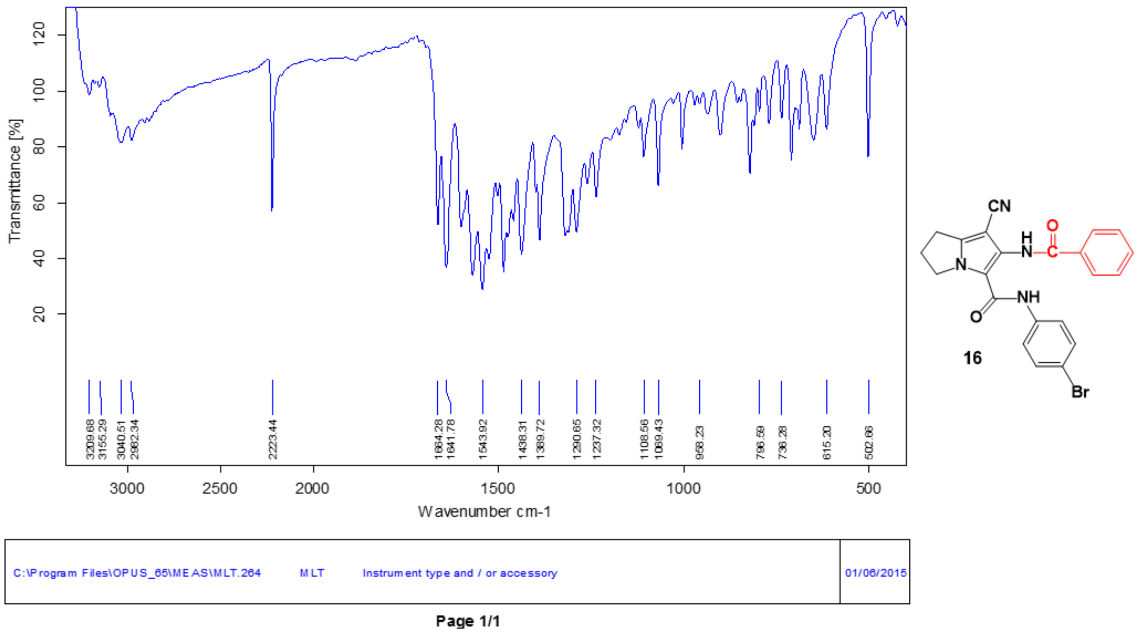

Figure S5: IR spectrum of compound 16

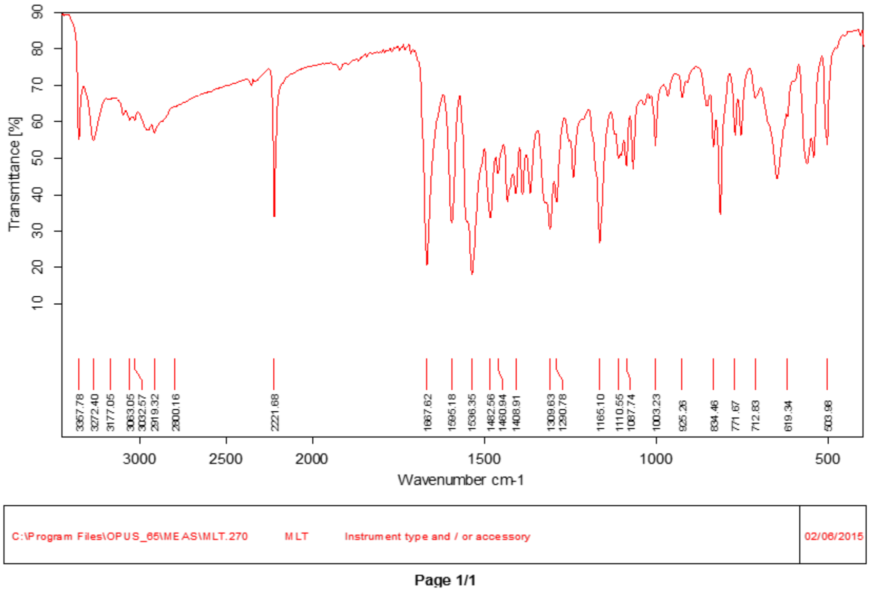

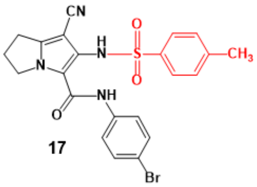

Figure S6: IR spectrum of compound 17

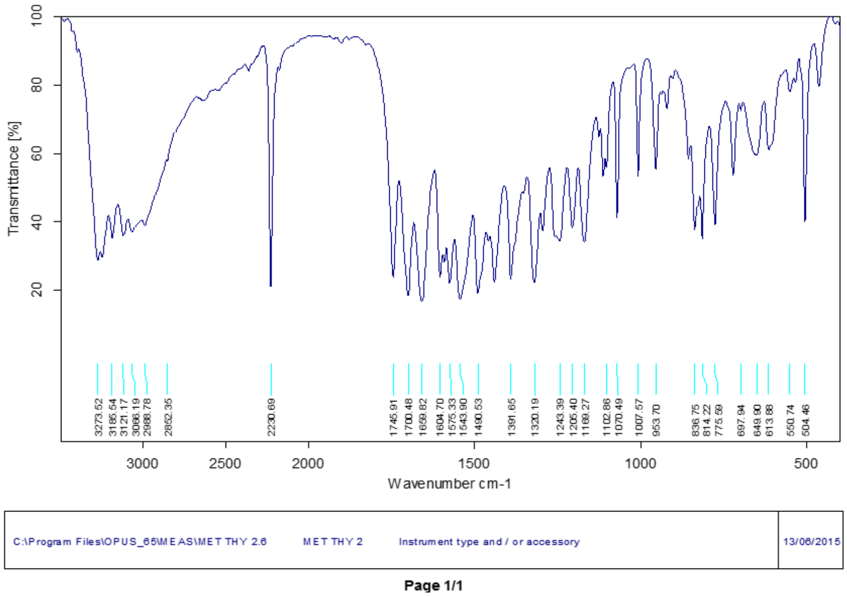

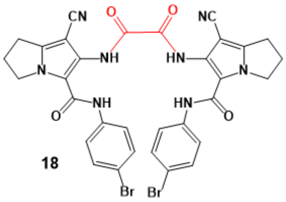

Figure S7: IR spectrum of compound 18

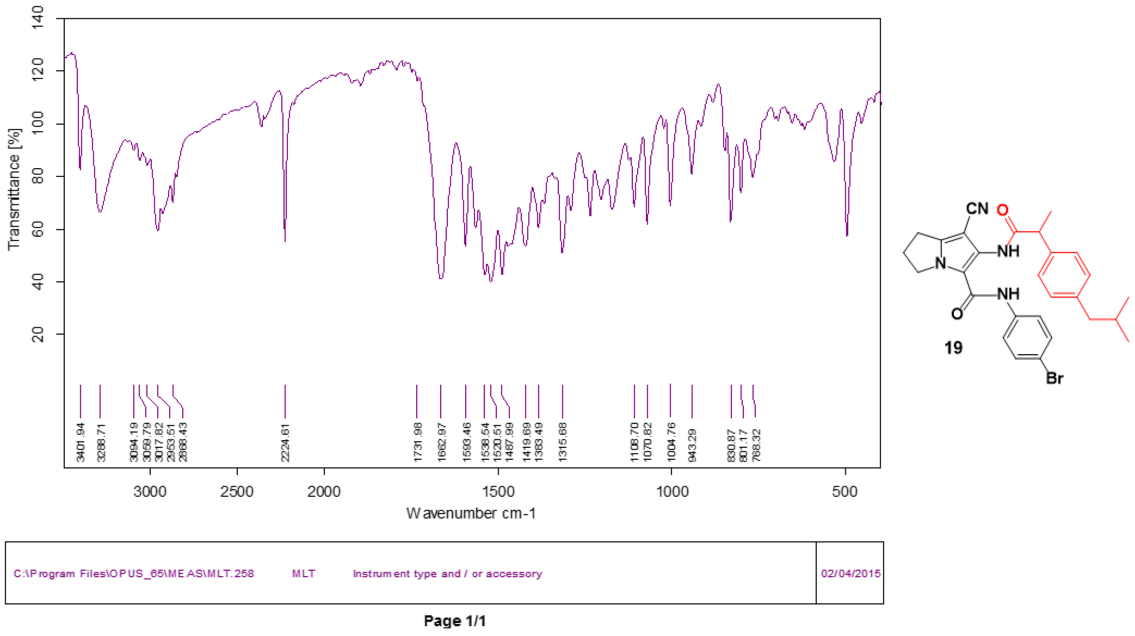

Figure S8: IR spectrum of compound 19

Mass Spectra

Mass spectra were recorded on Shimadzu GCMS QP5050A spectrometer, at 70 eV (EI) at the regional center for mycology and biotechnology, Al-Azhar University.

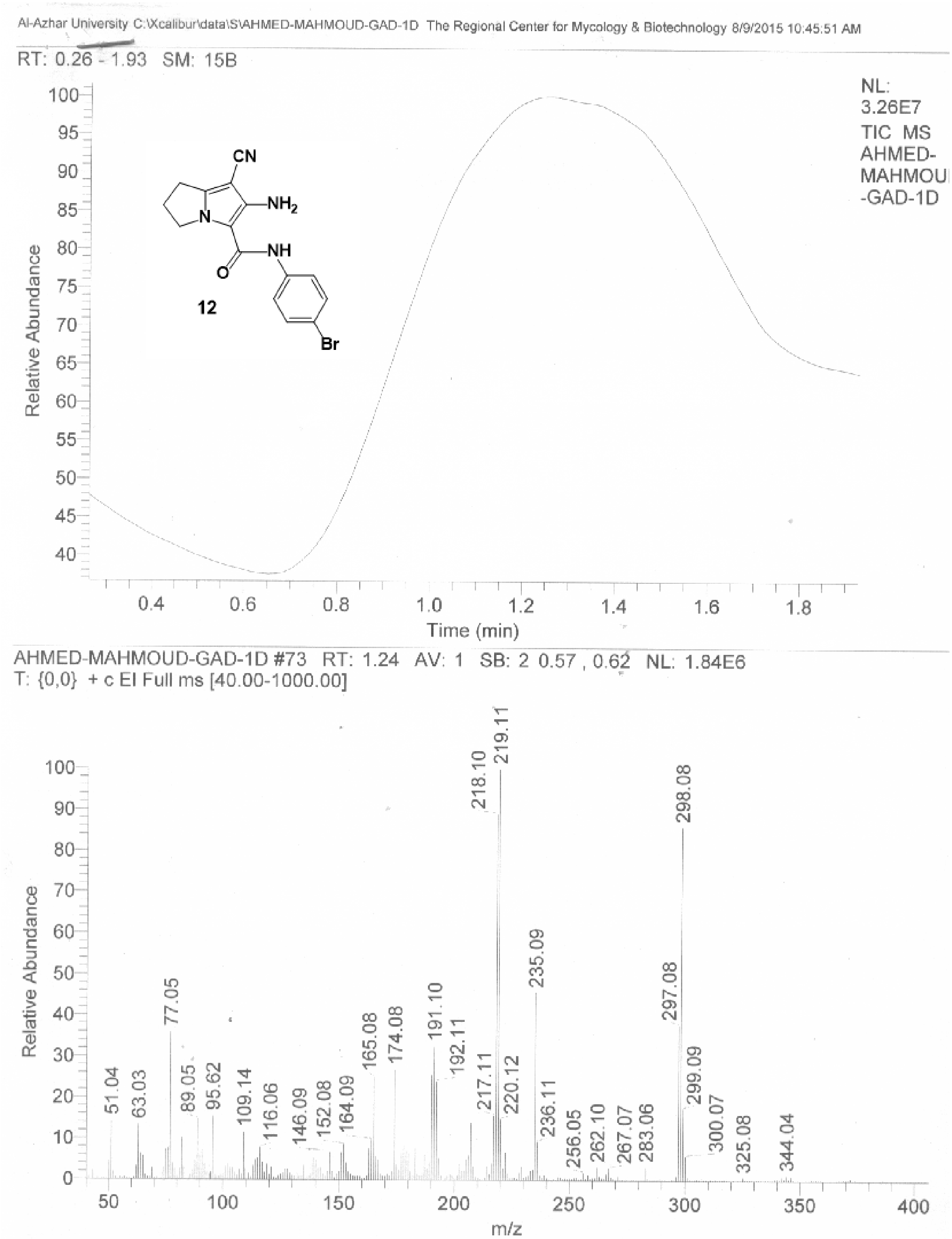

Figure S9: Mass spectrum of compound 12.

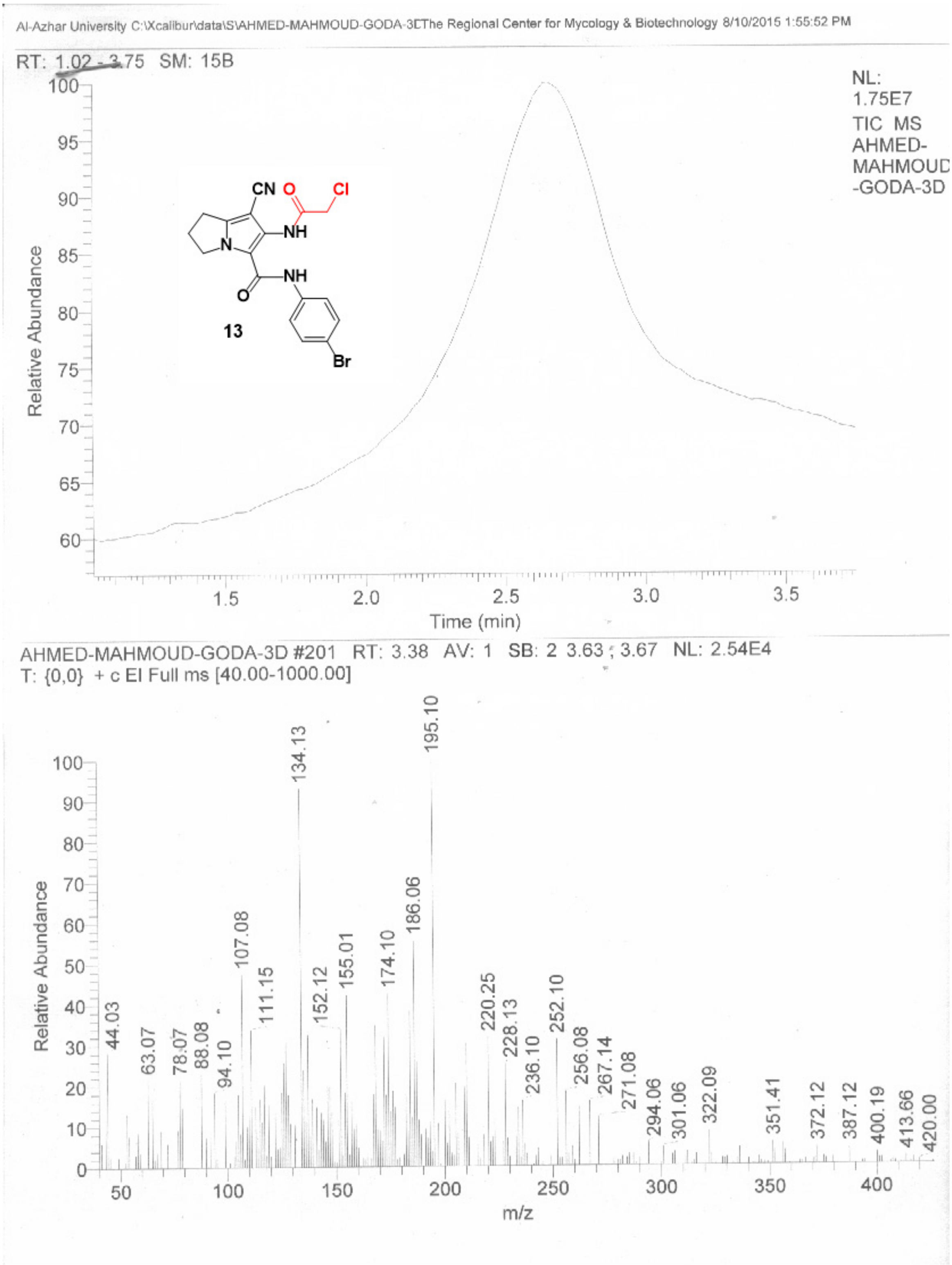

Figure S10: Mass spectrum of compound 13

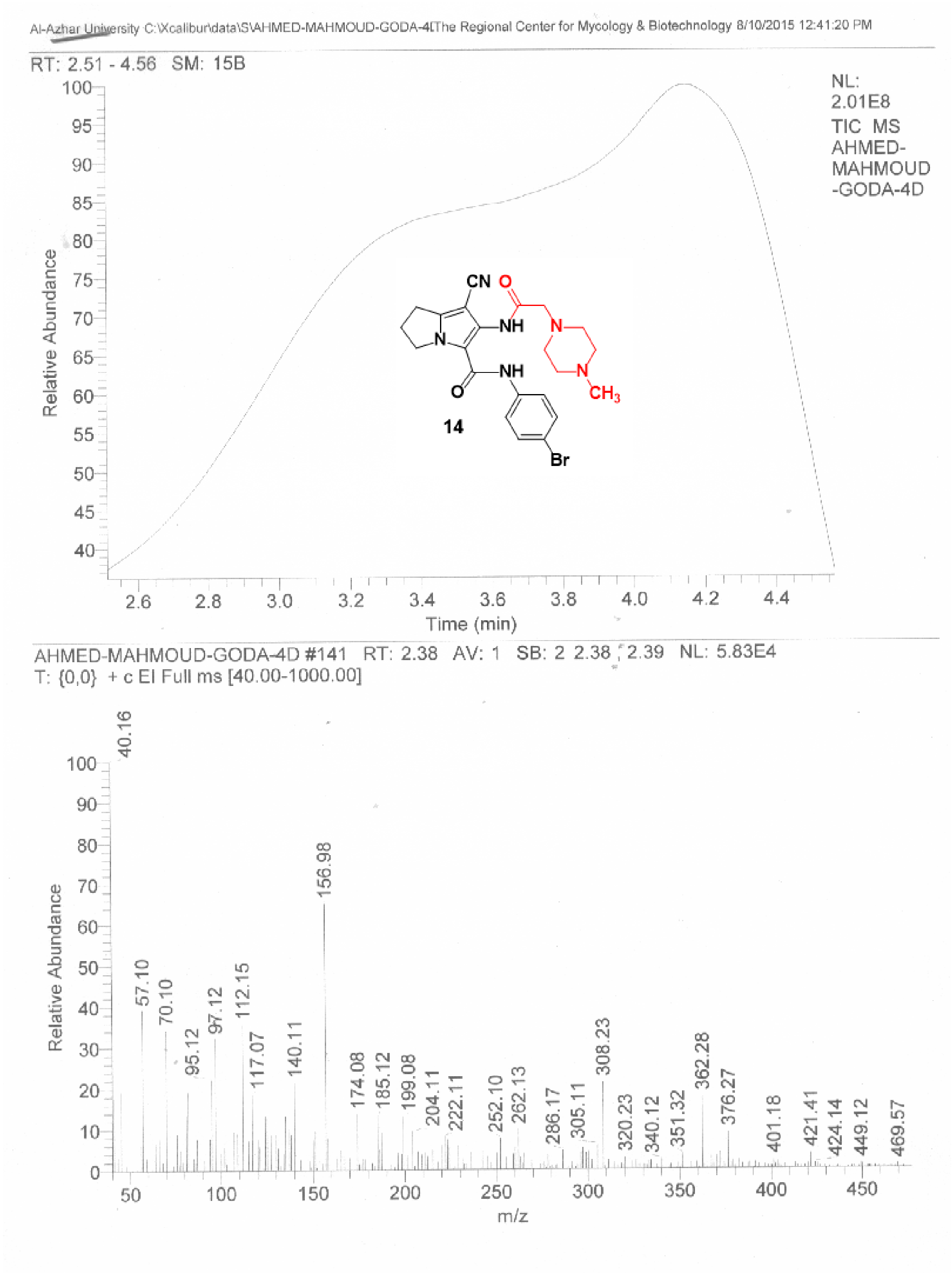

Figure S11: Mass spectrum of compound 14

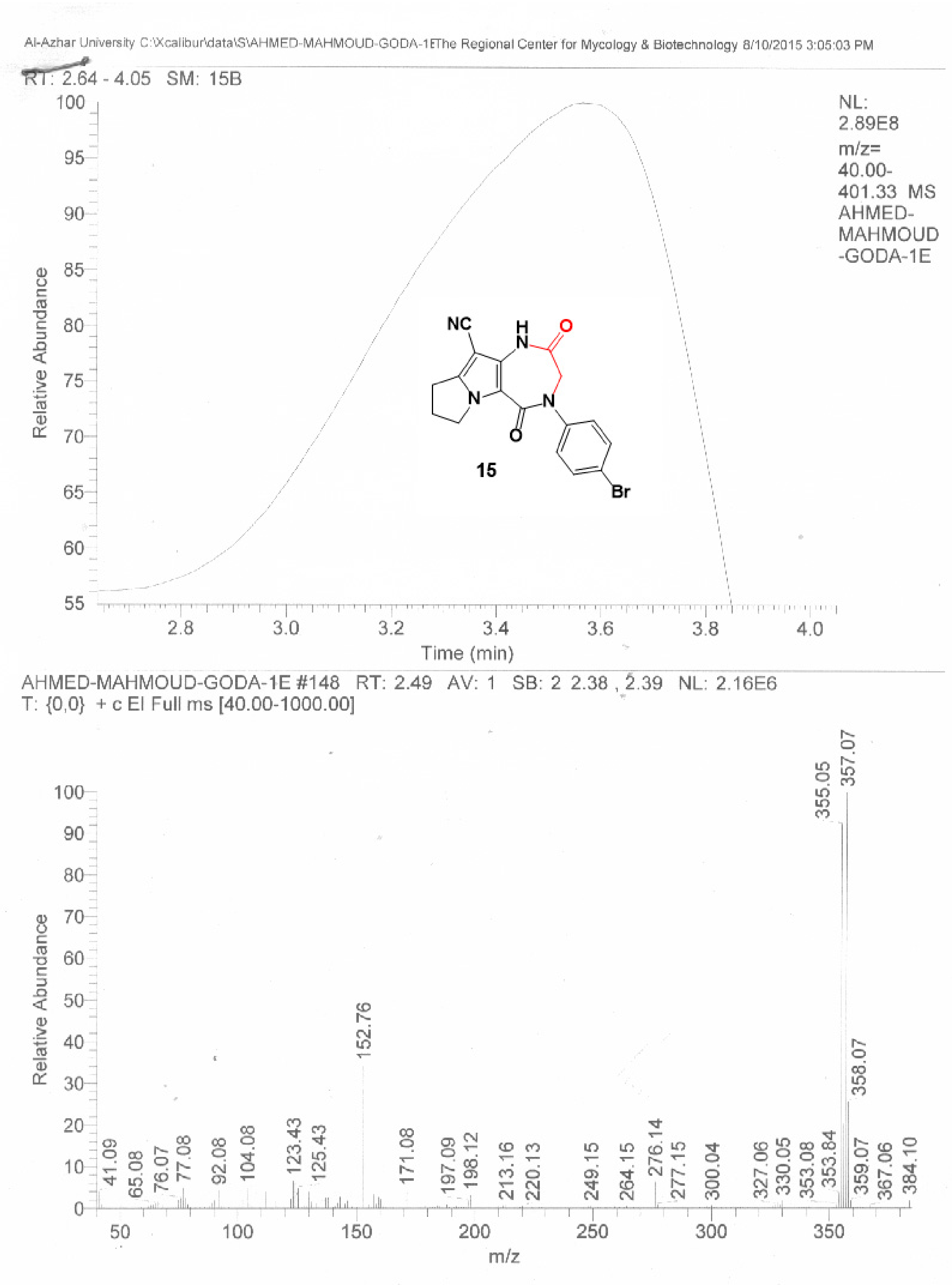

Figure S12: Mass spectrum of compound 15

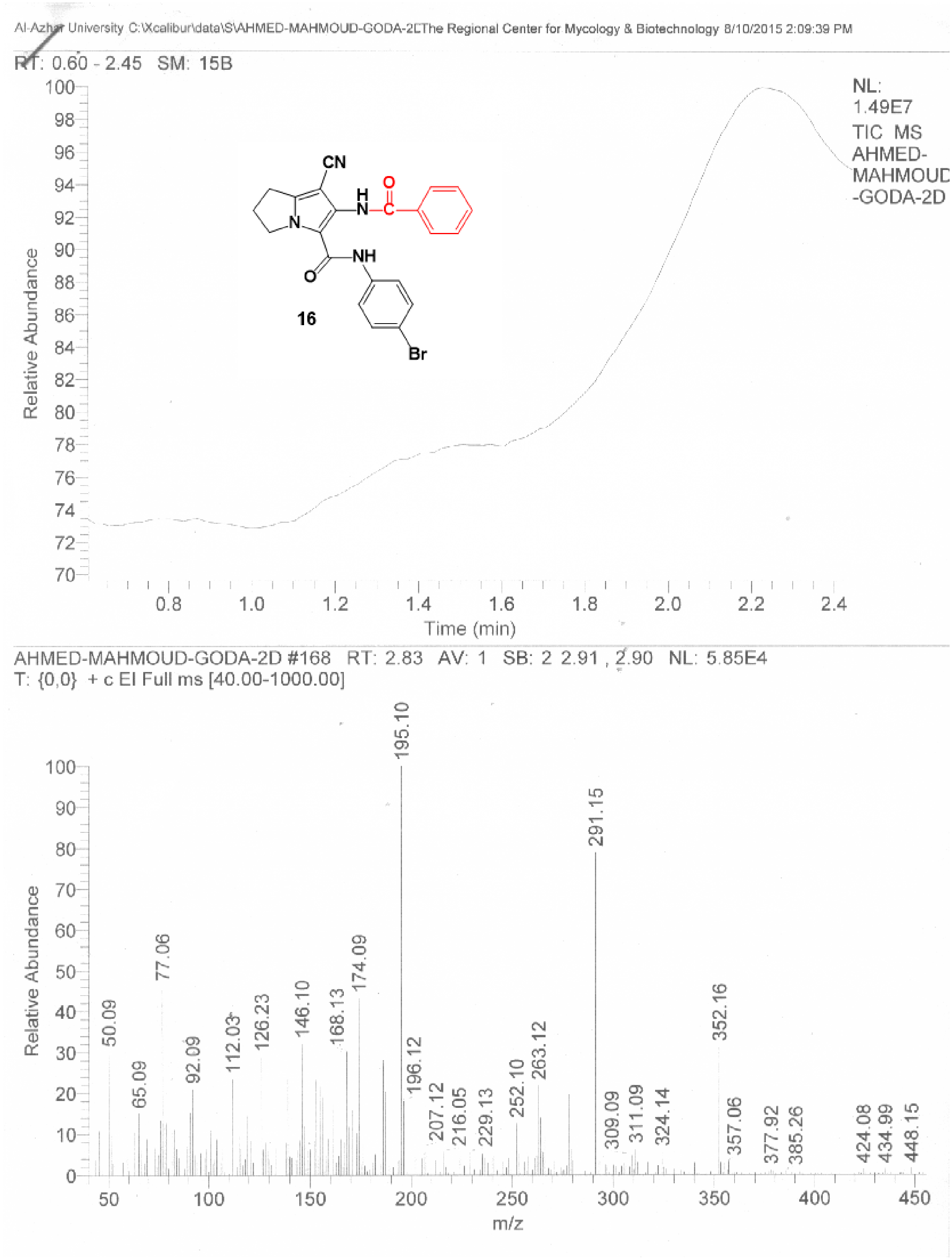

Figure S13: Mass spectrum of compound 16

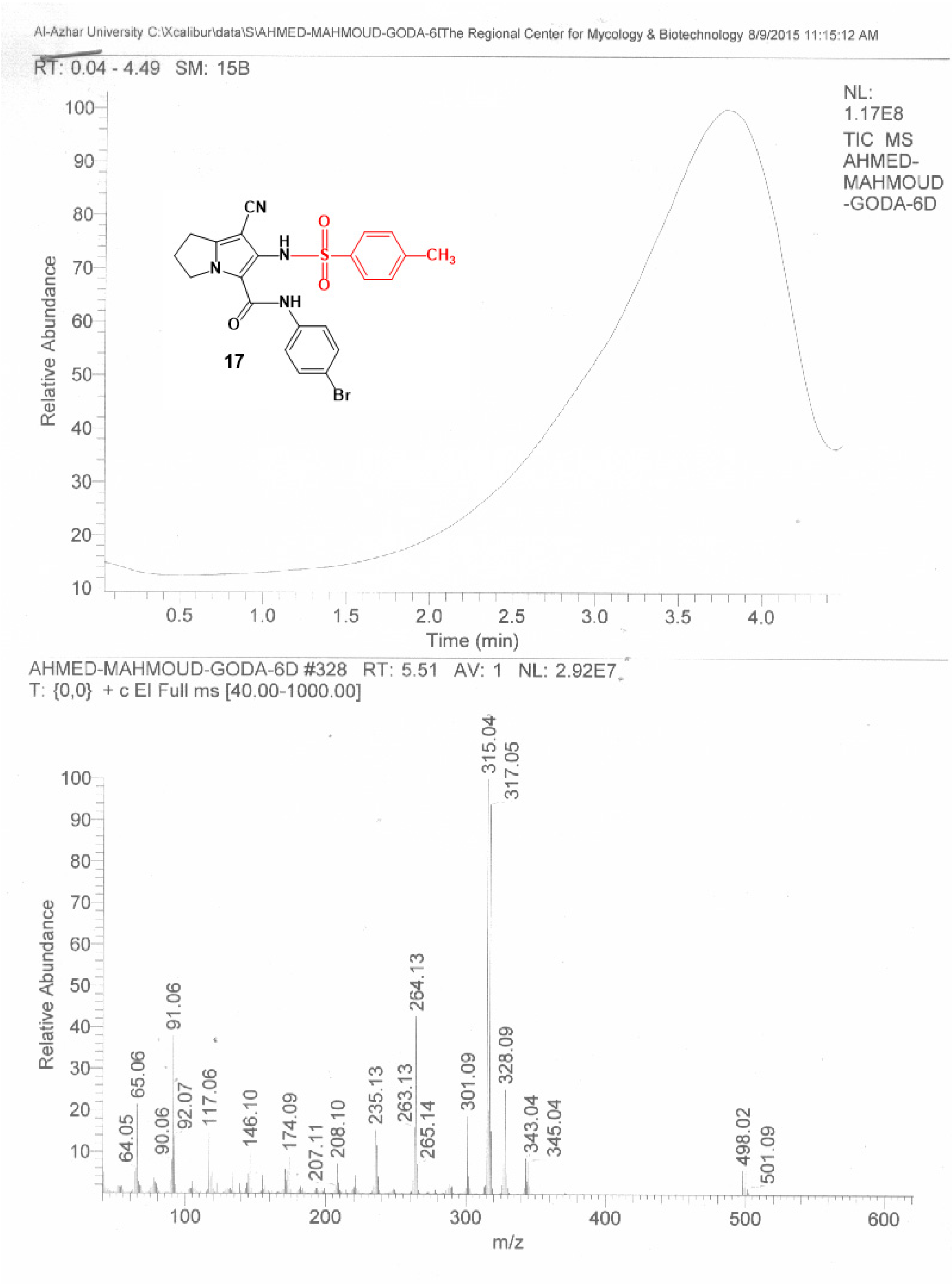

Figure S14: Mass spectrum of compound 17

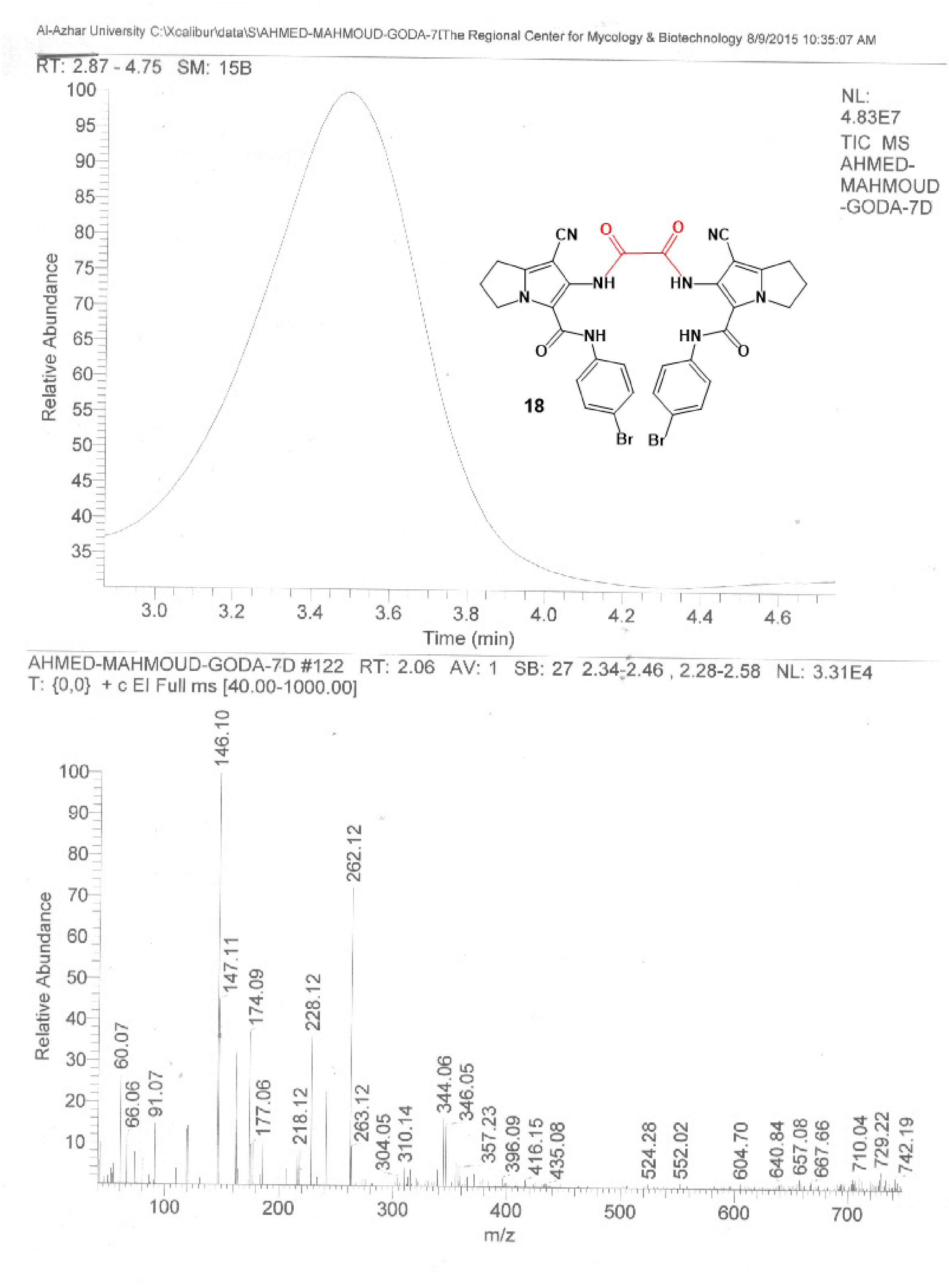

Figure S15: Mass spectrum of compound 18

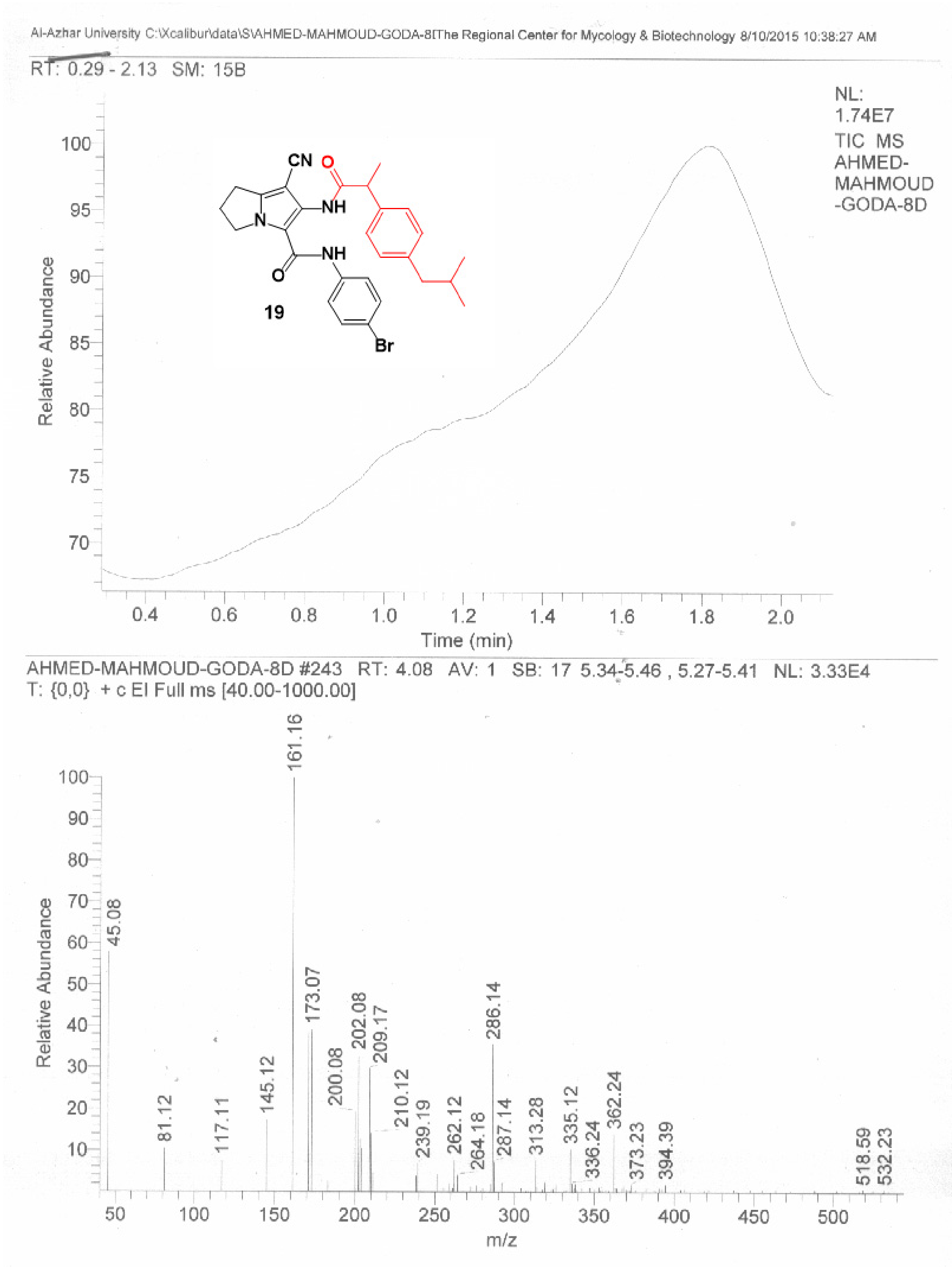

Figure S16: Mass spectrum of compound 19

1H-NMR and 13C-NMR Spectra

1H-NMR spectra were recorded on a BRUKER AVANCE II spectrometer (at the faculty of pharmacy, Umm Al-Qura University) at 500 MHz in the specified solvent, chemical shifts were reported on the δ scale and were related to that of the solvent and J values are given in Hz. 13C NMR and DEPT135 spectra were obtained on a BRUKER AVANCE II at 125 MHz (at the faculty of pharmacy, Umm Al-Qura University).

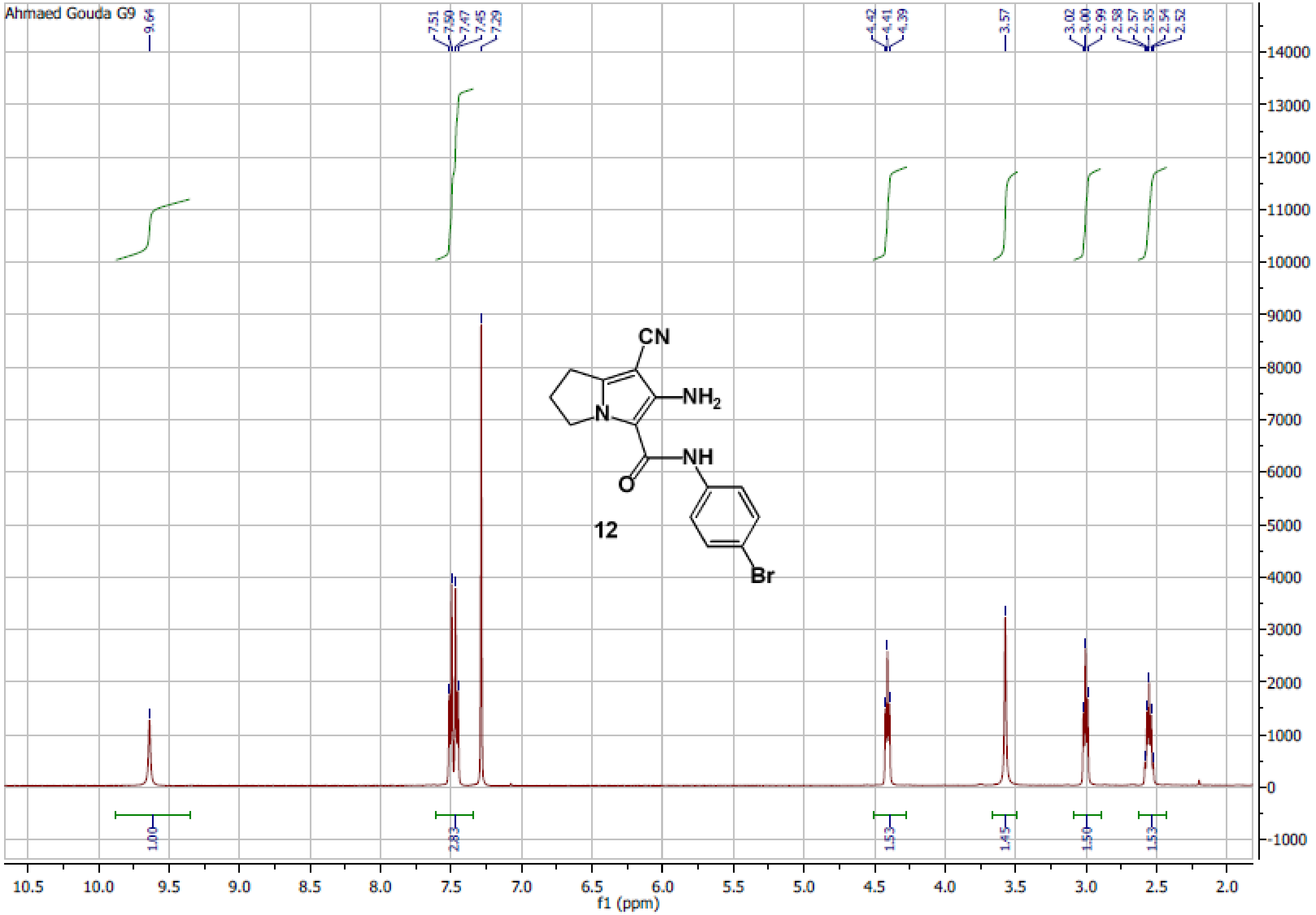

Figure S17a: 1H-NMR (CDCl3, 500 MHz, δ ppm) spectrum of compound 12

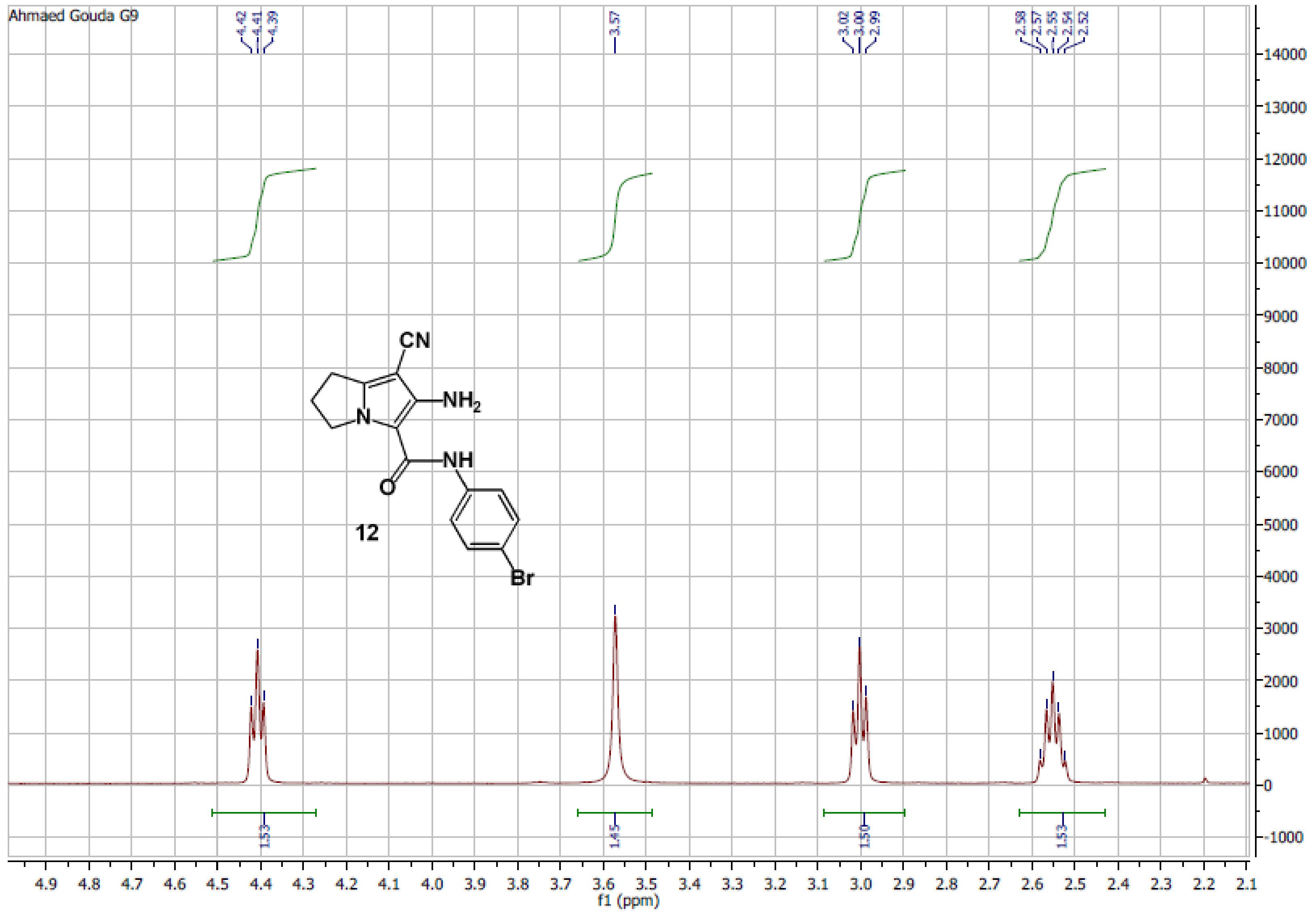

Figure S17b: 1H-NMR (CDCl3, 500 MHz, δ ppm) spectrum of compound 12 (ZOOM on Aliphatic Protons)

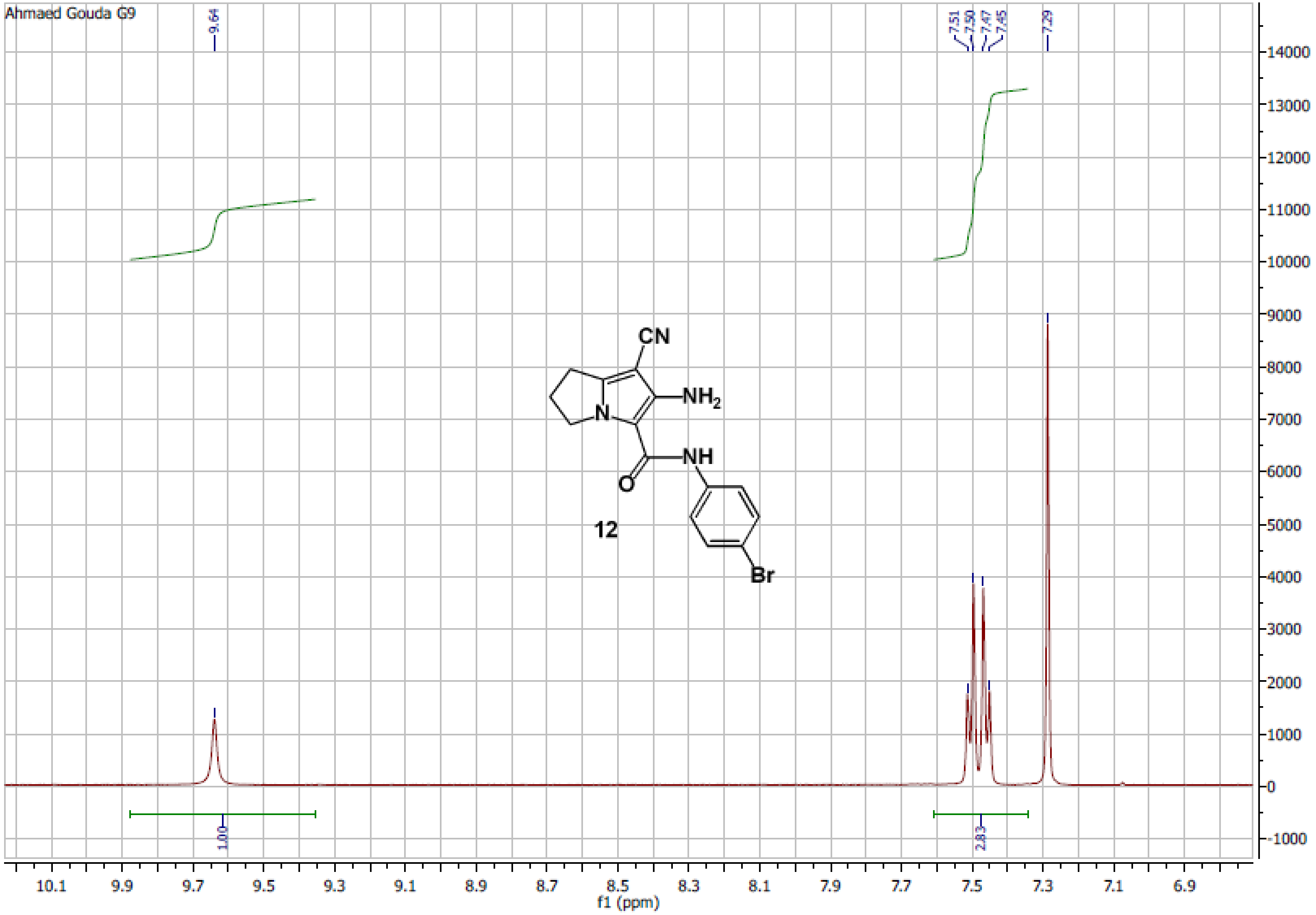

Figure S17c: 1H-NMR (CDCl3, 500 MHz, δ ppm) spectrum of compound 12 (ZOOM on Aromatic Protons)

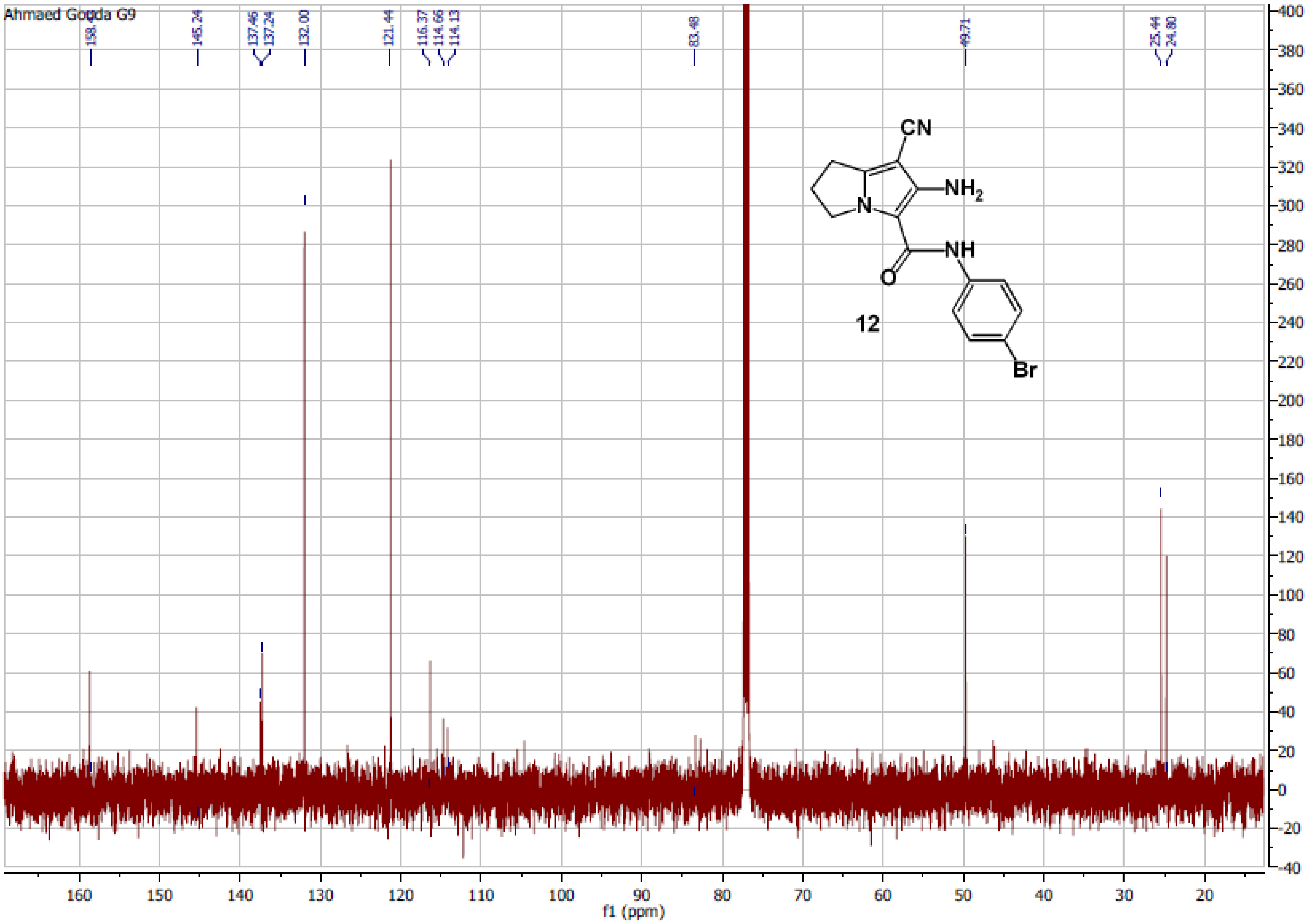

Figure S18a: 13C-NMR (CDCl3, 125 MHz, δ ppm) spectrum of compound 12

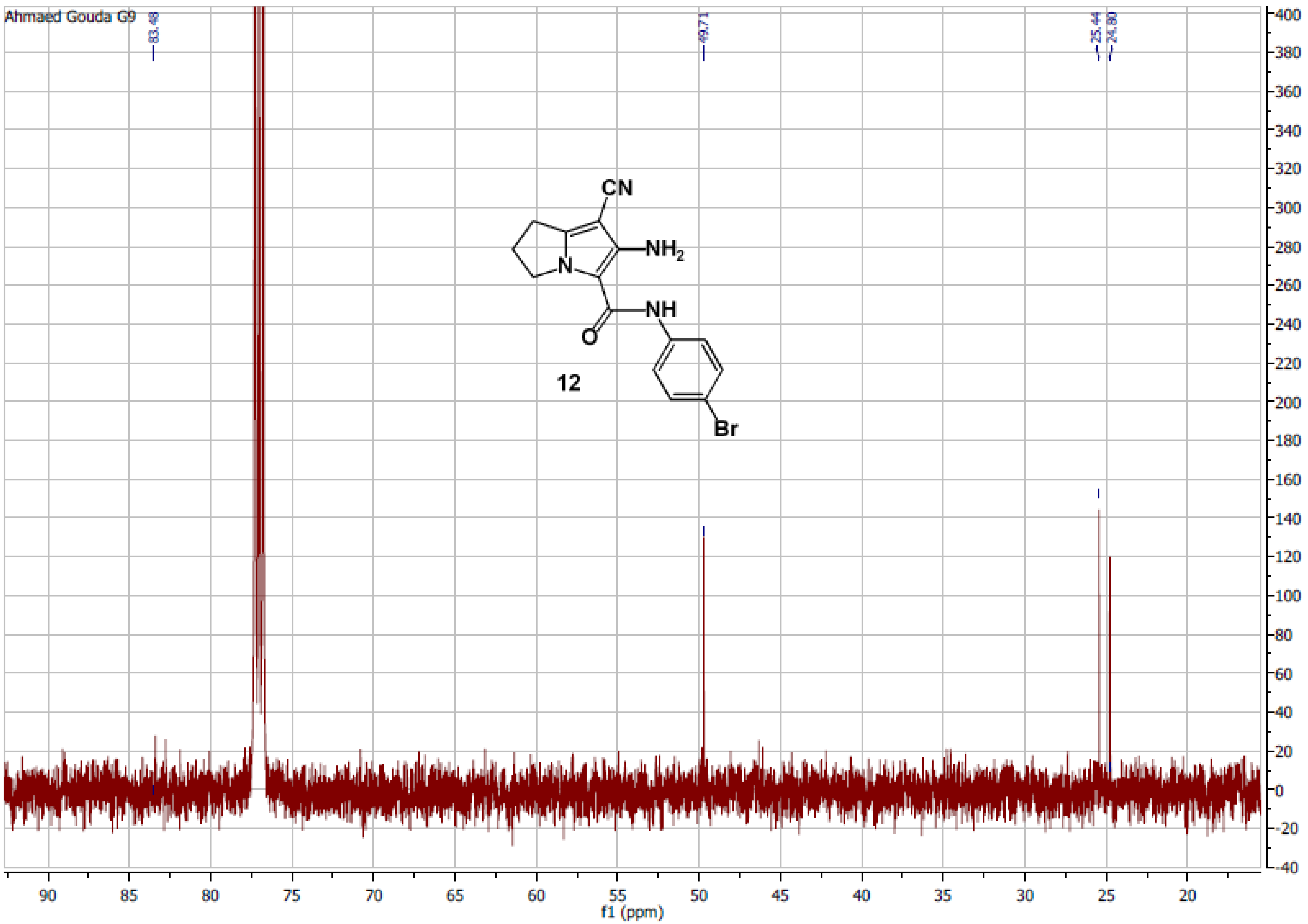

Figure S18b: 13C-NMR (CDCl3, 125 MHz, δ ppm) spectrum of compound 12 (ZOOM on Aliphatic Carbons)

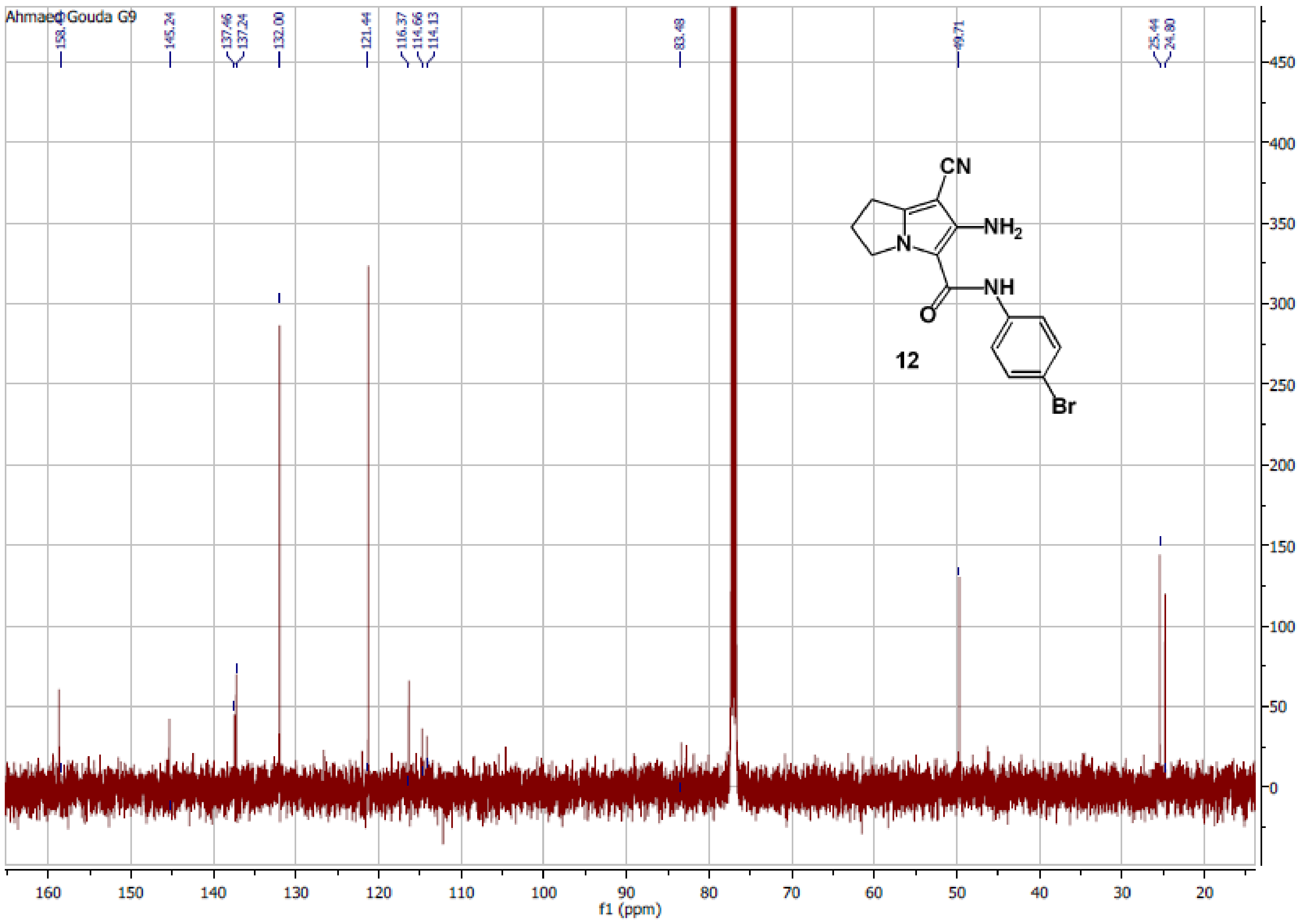

Figure S18c: 13C-NMR (CDCl3, 125 MHz, δ ppm) spectrum of compound 12 (ZOOM on Aromatic Carbons)

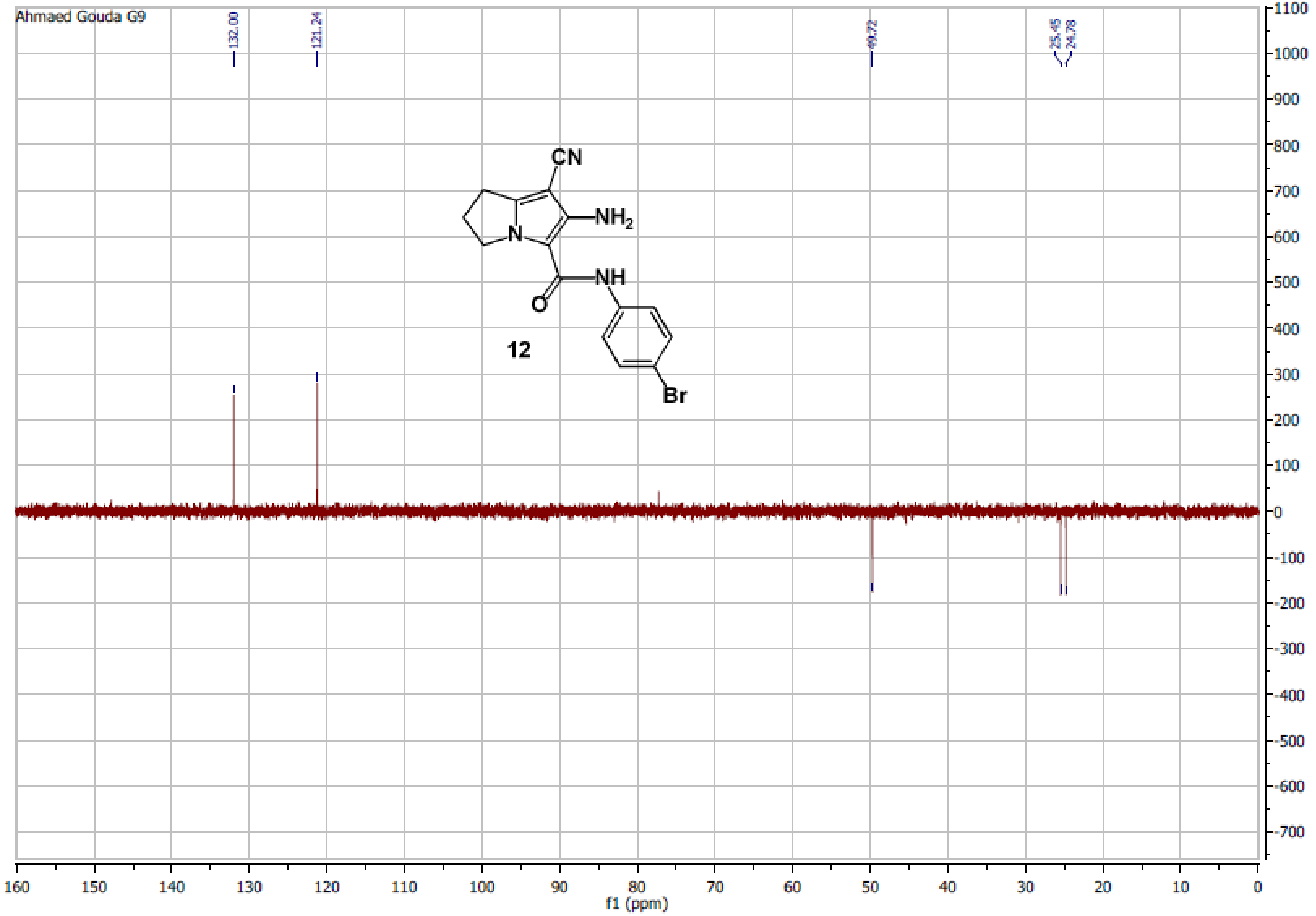

Figure S19: DEPT 135 of compound 12

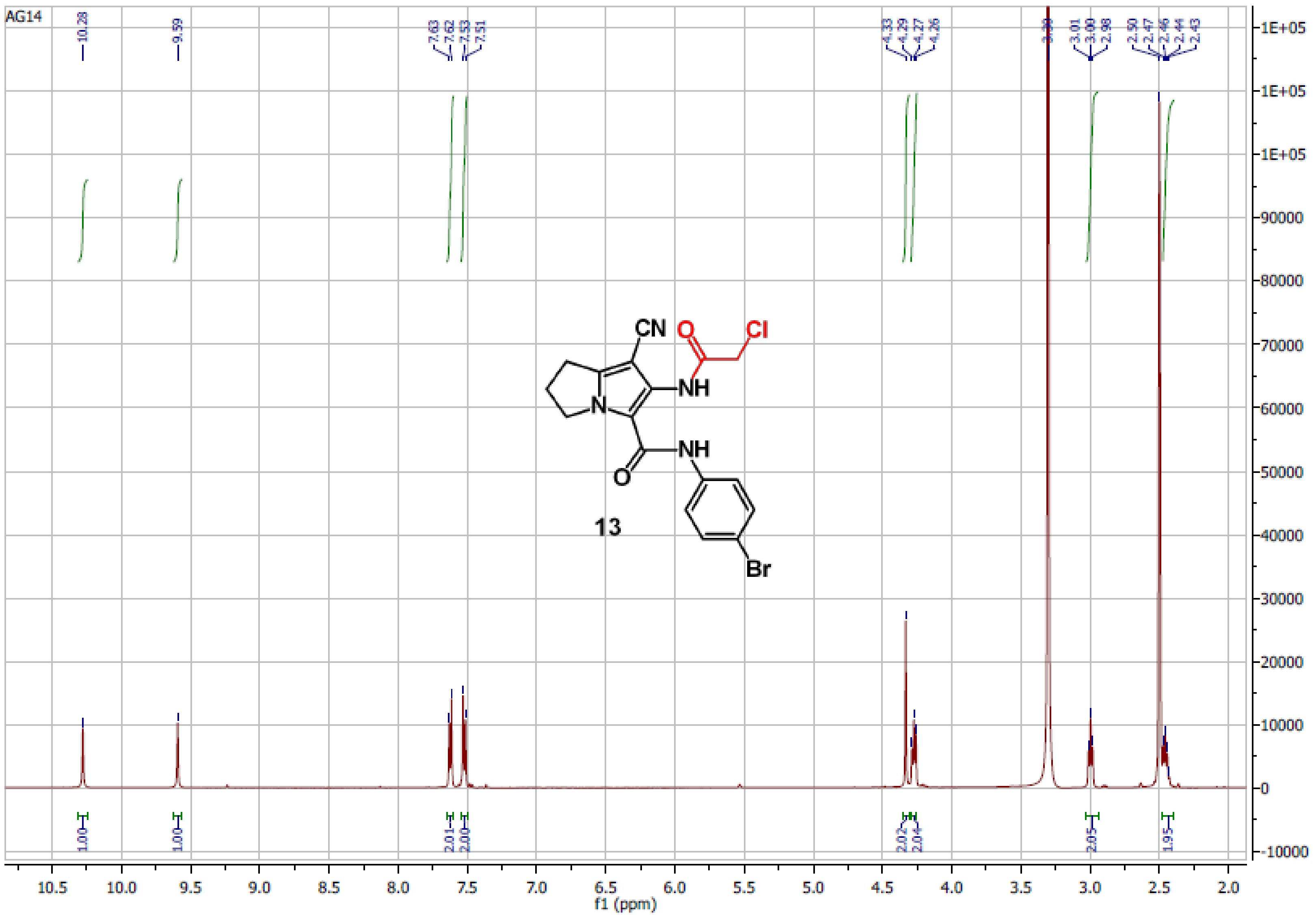

Figure S20a: 1H-NMR (DMSO, 500 MHz, δ ppm) spectrum of compound 13

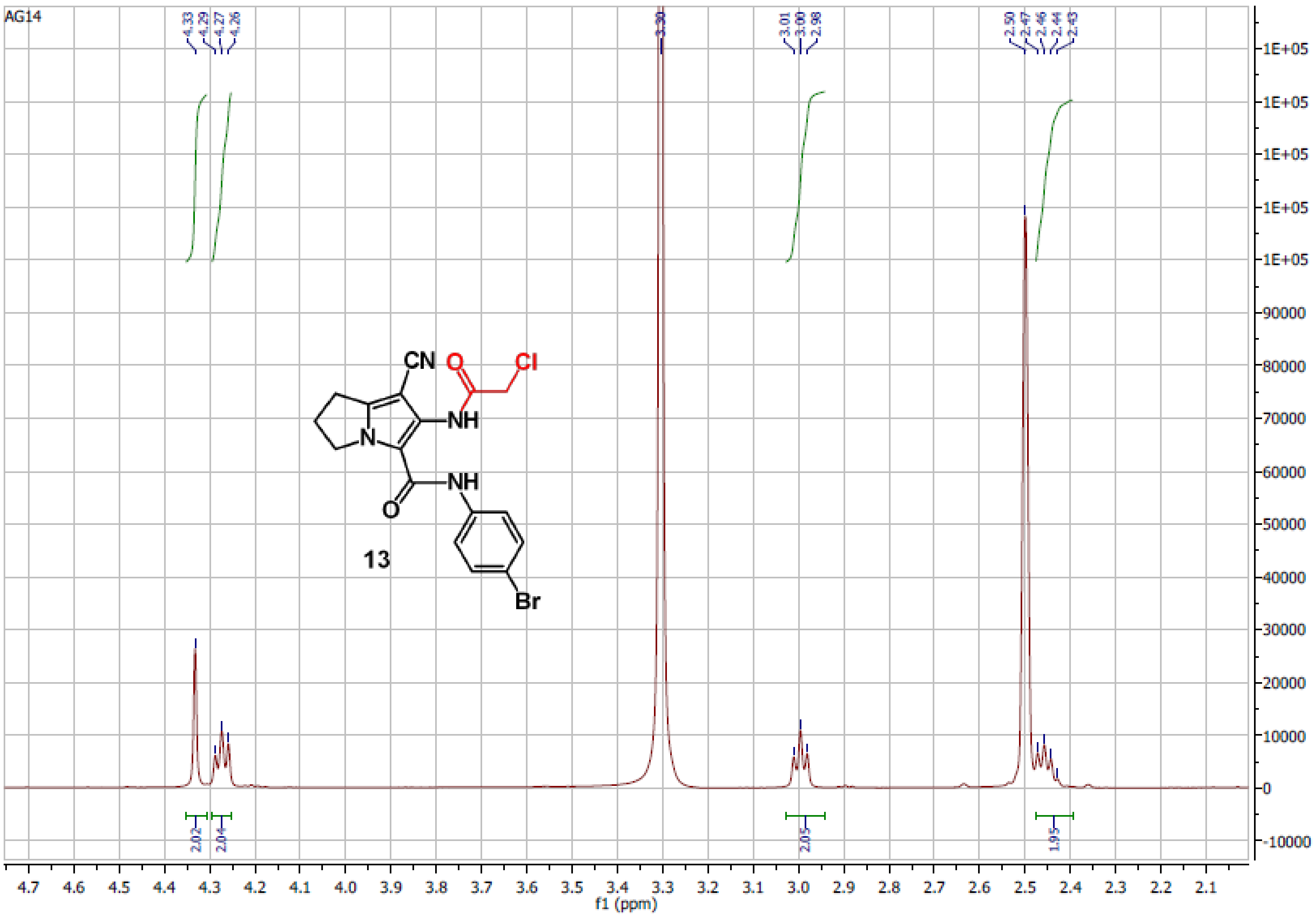

Figure S20b: 1H-NMR (DMSO, 500 MHz, δ ppm) spectrum of compound 13 (ZOOM on Aliphatic Protons)

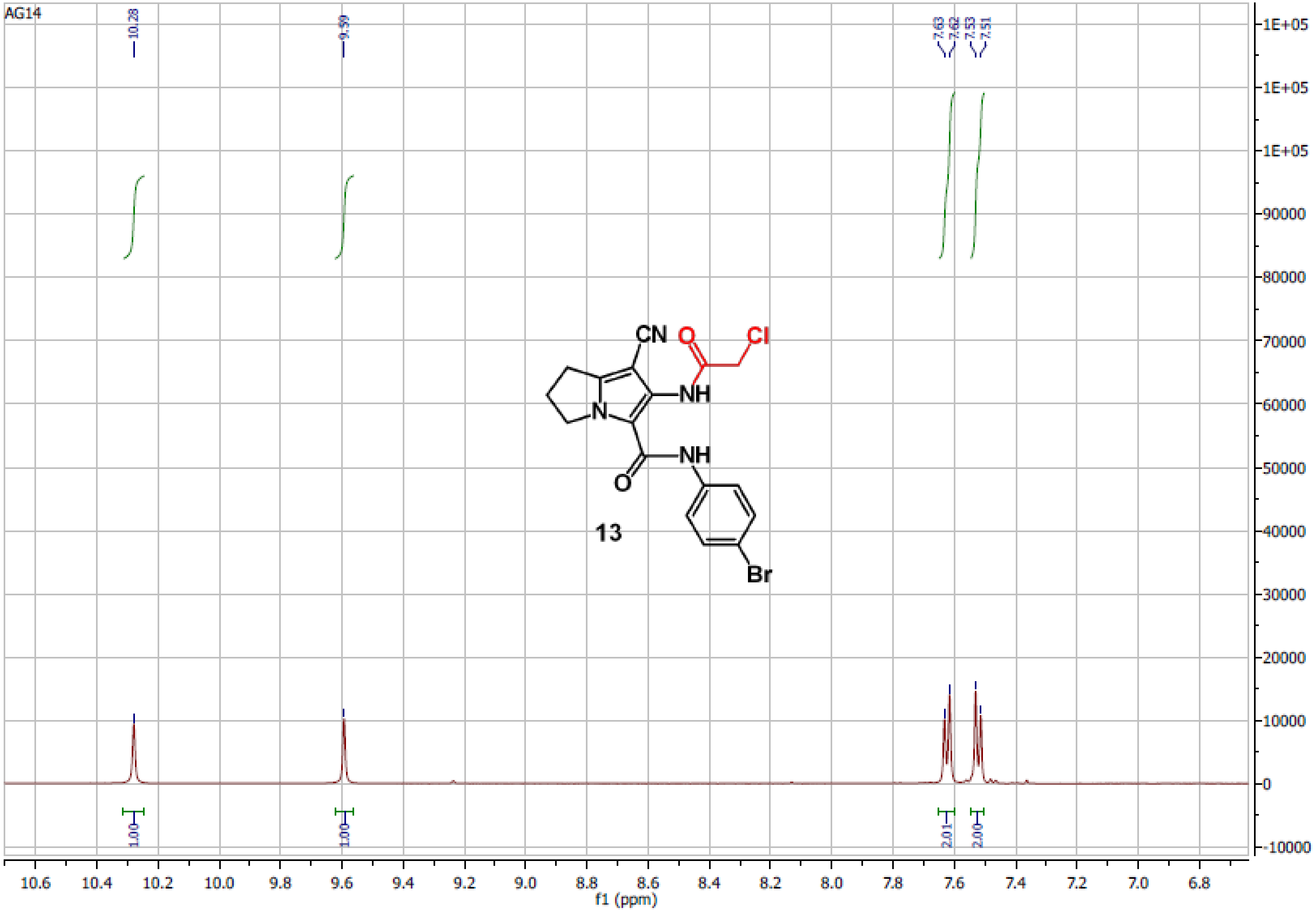

Figure S20c: 1H-NMR (DMSO, 500 MHz, δ ppm) spectrum of compound 13 (ZOOM on Aromatic Protons)

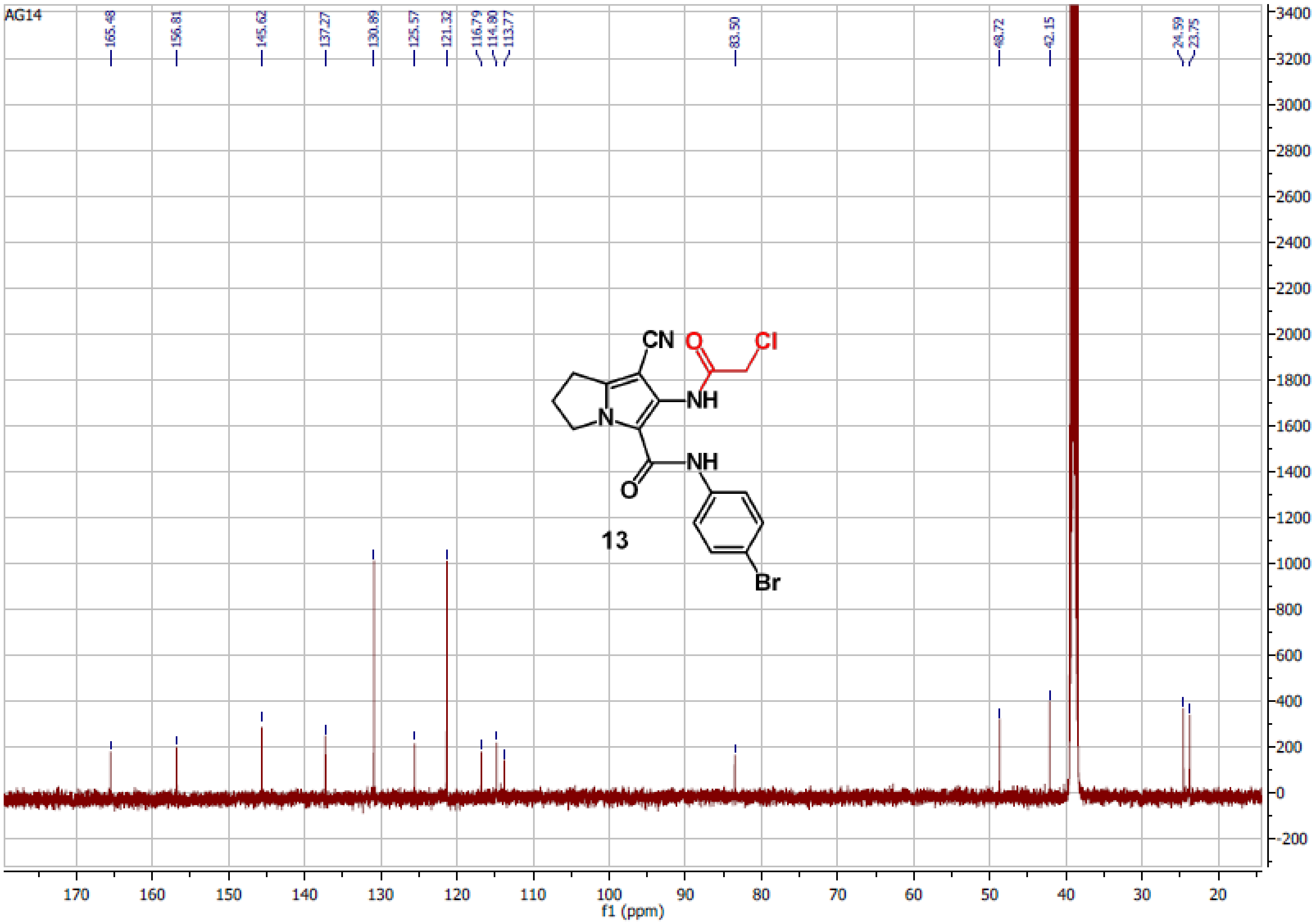

Figure S21a: 13C-NMR (DMSO, 125 MHz, δ ppm) spectrum of compound 13

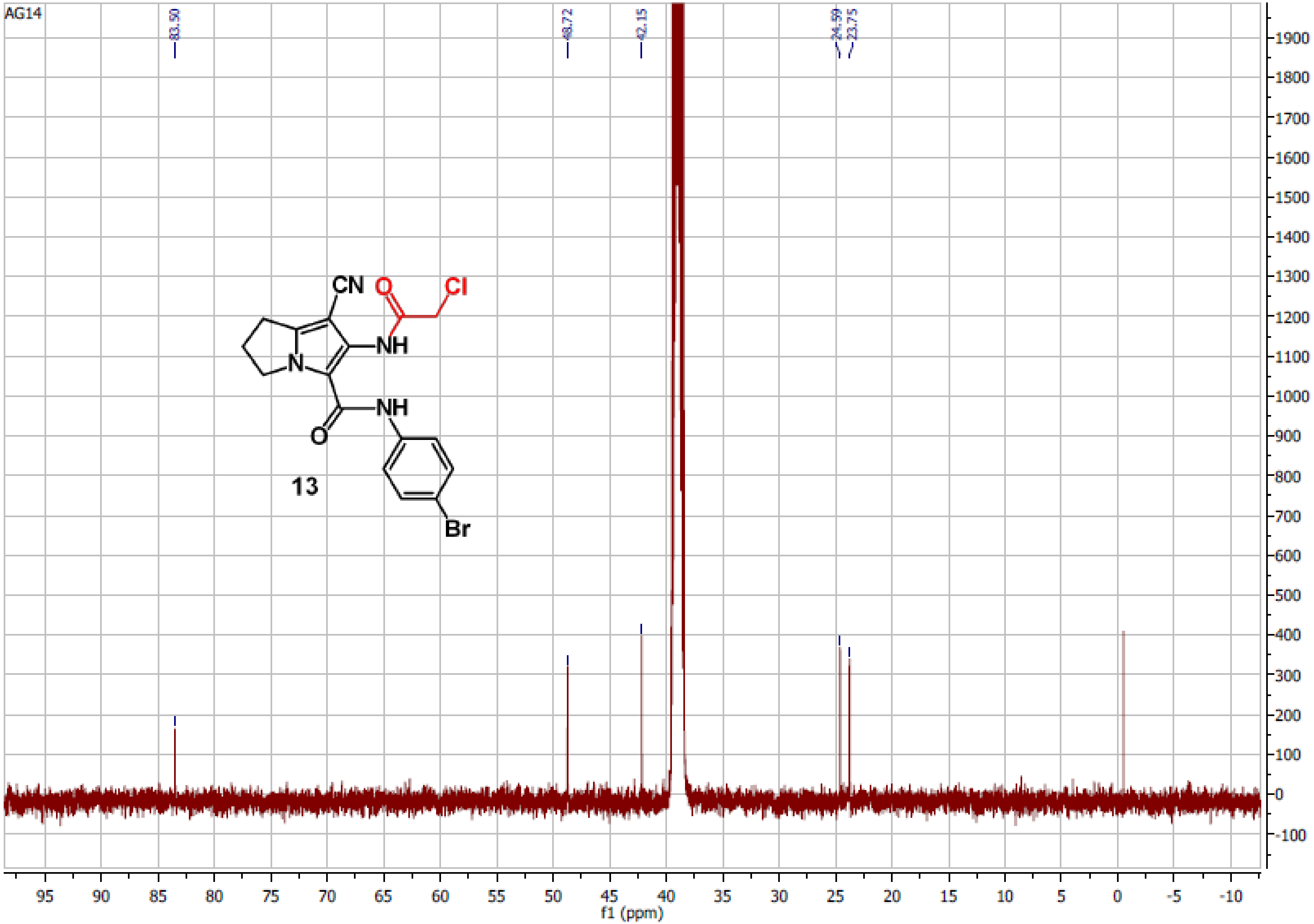

Figure S21b: 13C-NMR (DMSO, 125 MHz, δ ppm) spectrum of compound 13 (ZOOM on Aliphatic Carbons)

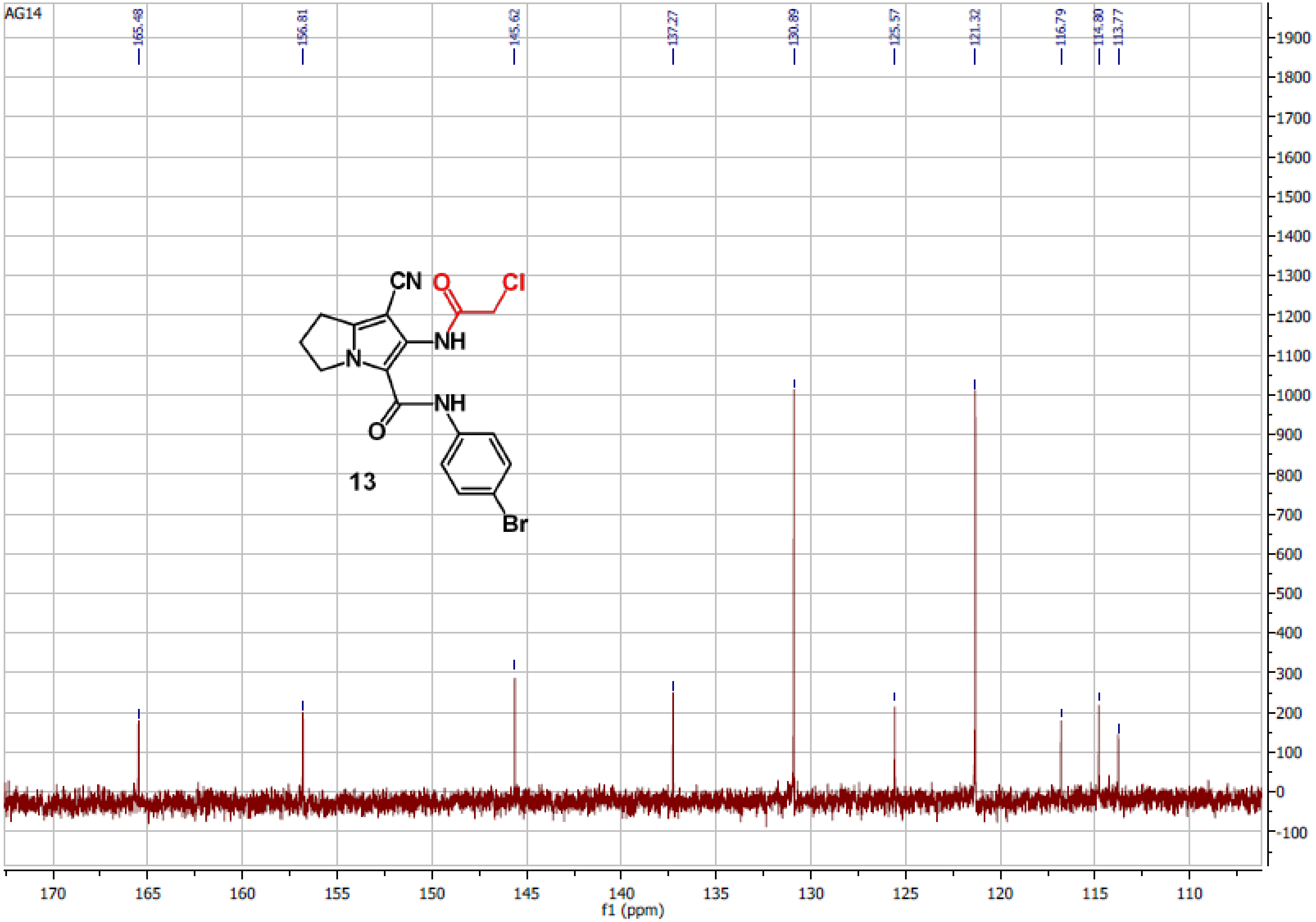

Figure S21c: 13C-NMR (DMSO, 125 MHz, δ ppm) spectrum of compound 13 (ZOOM on Aromatic Carbons)

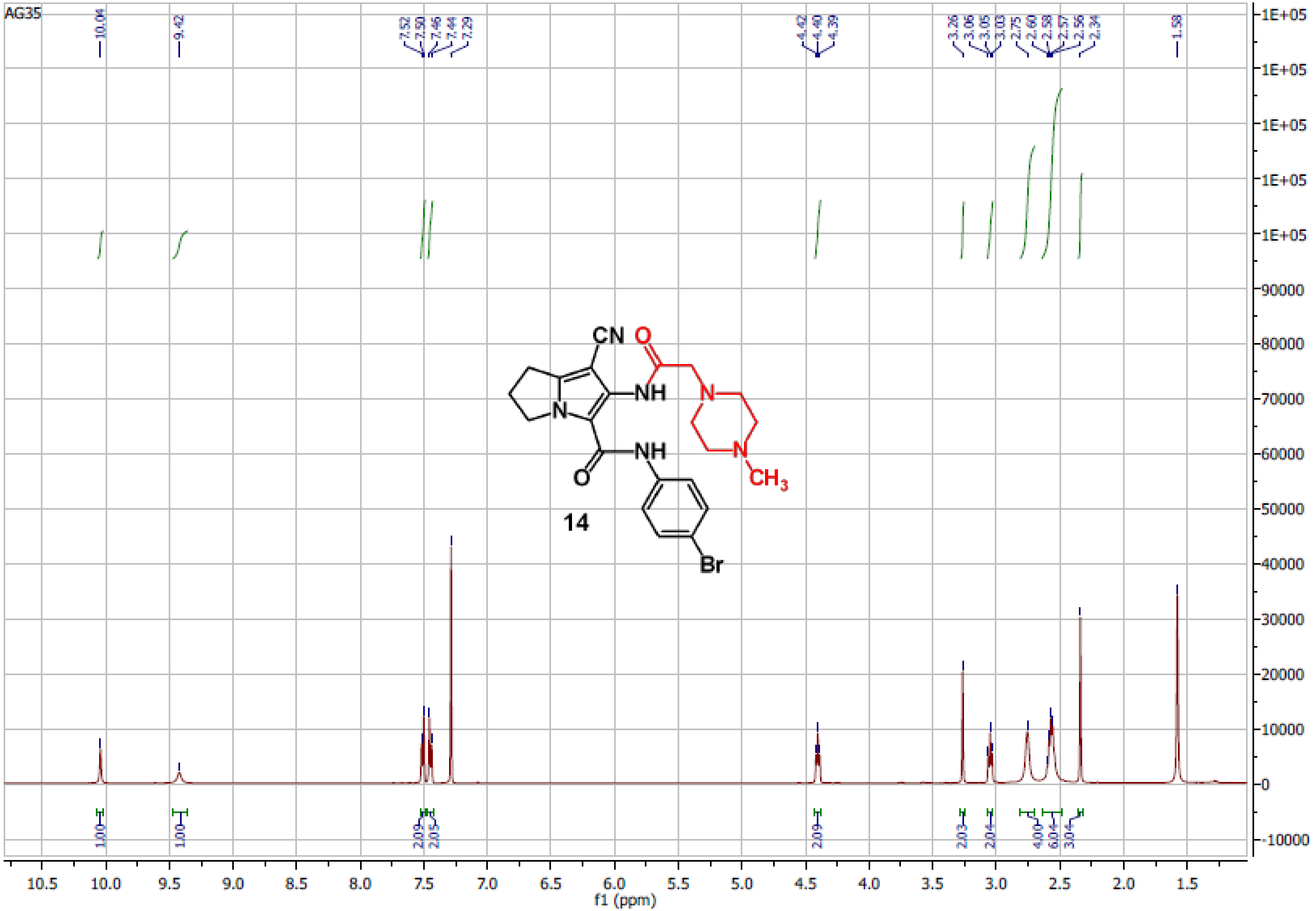

Figure S22a: 1H-NMR (CDCl3, 500 MHz, δ ppm) spectrum of compound 14

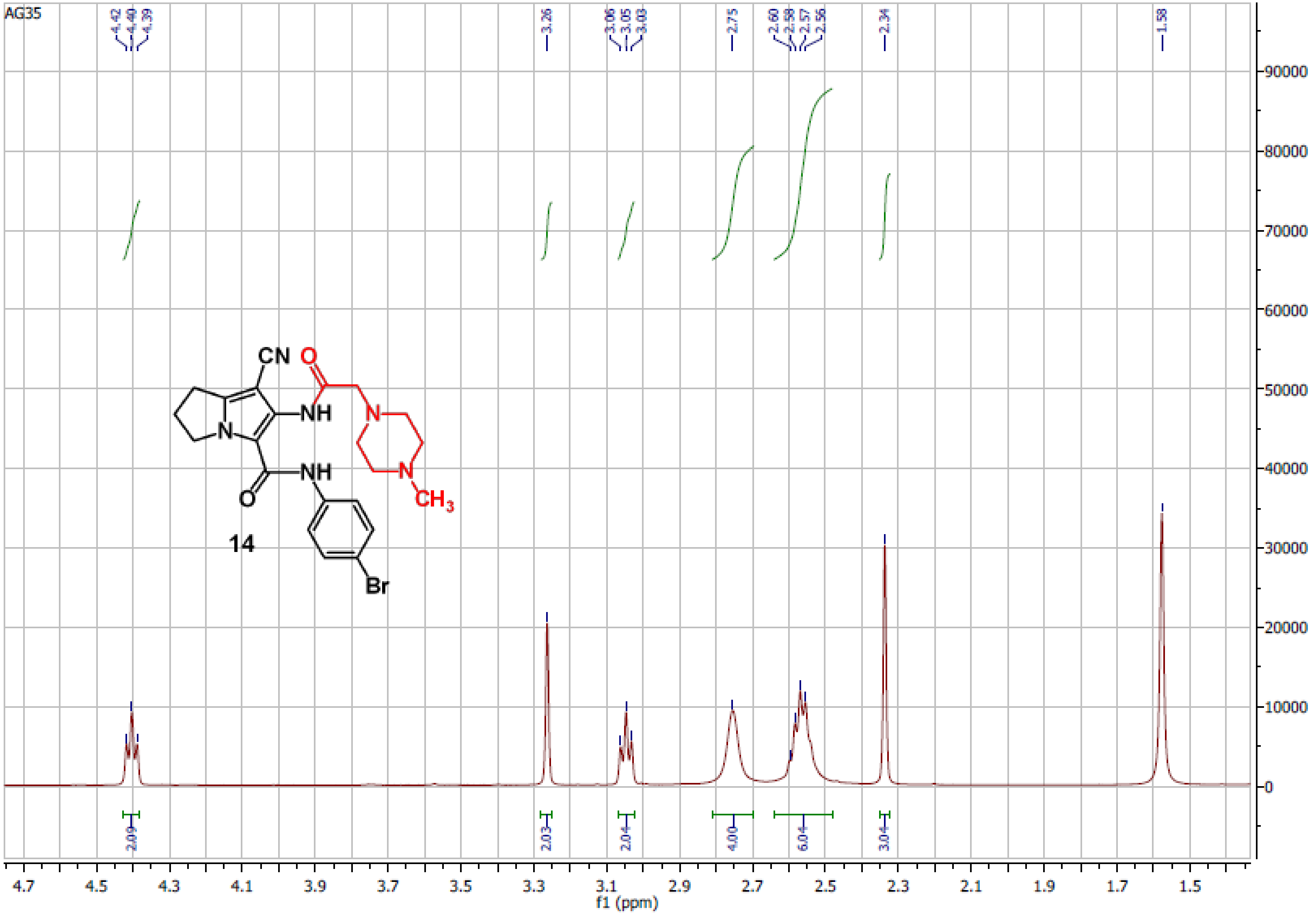

Figure S22b: 1H-NMR (CDCl3, 500 MHz, δ ppm) spectrum of compound 14 (ZOOM on Aliphatic Protons)

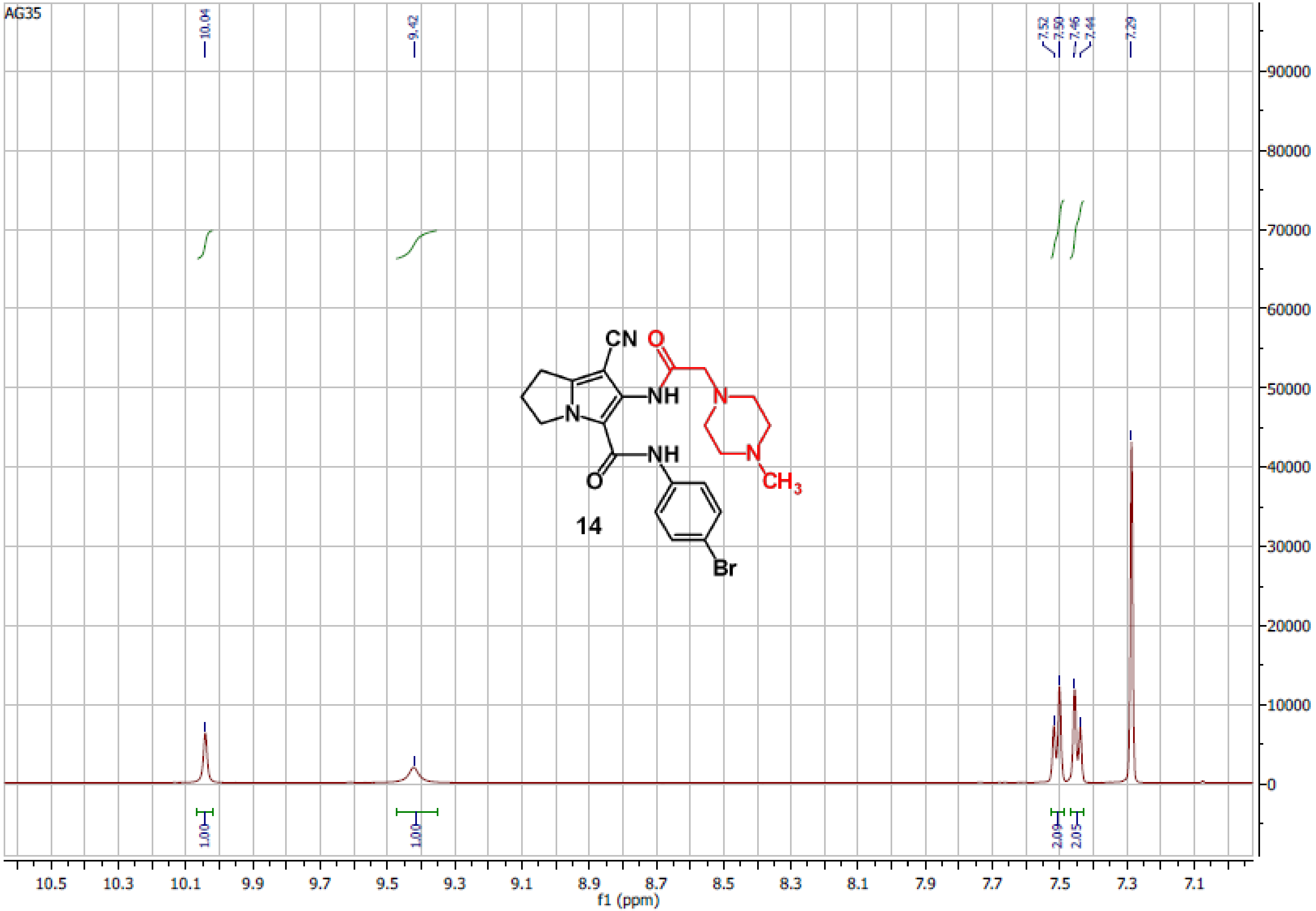

Figure S22c: 1H-NMR (CDCl3, 500 MHz, δ ppm) spectrum of compound 14 (ZOOM on Aromatic Protons)

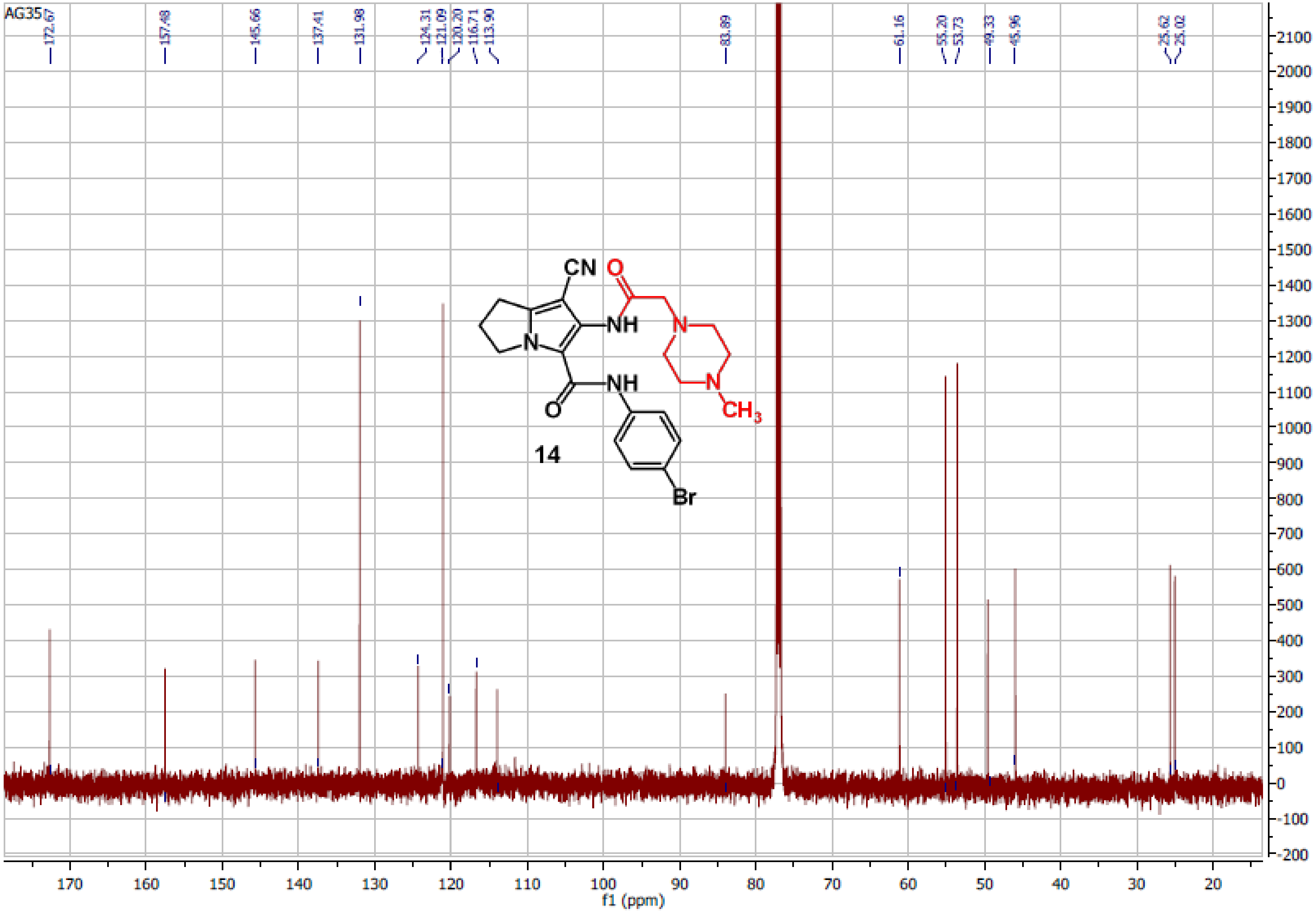

Figure S23a: 13C-NMR (CDCl3, 125 MHz, δ ppm) spectrum of compound 14

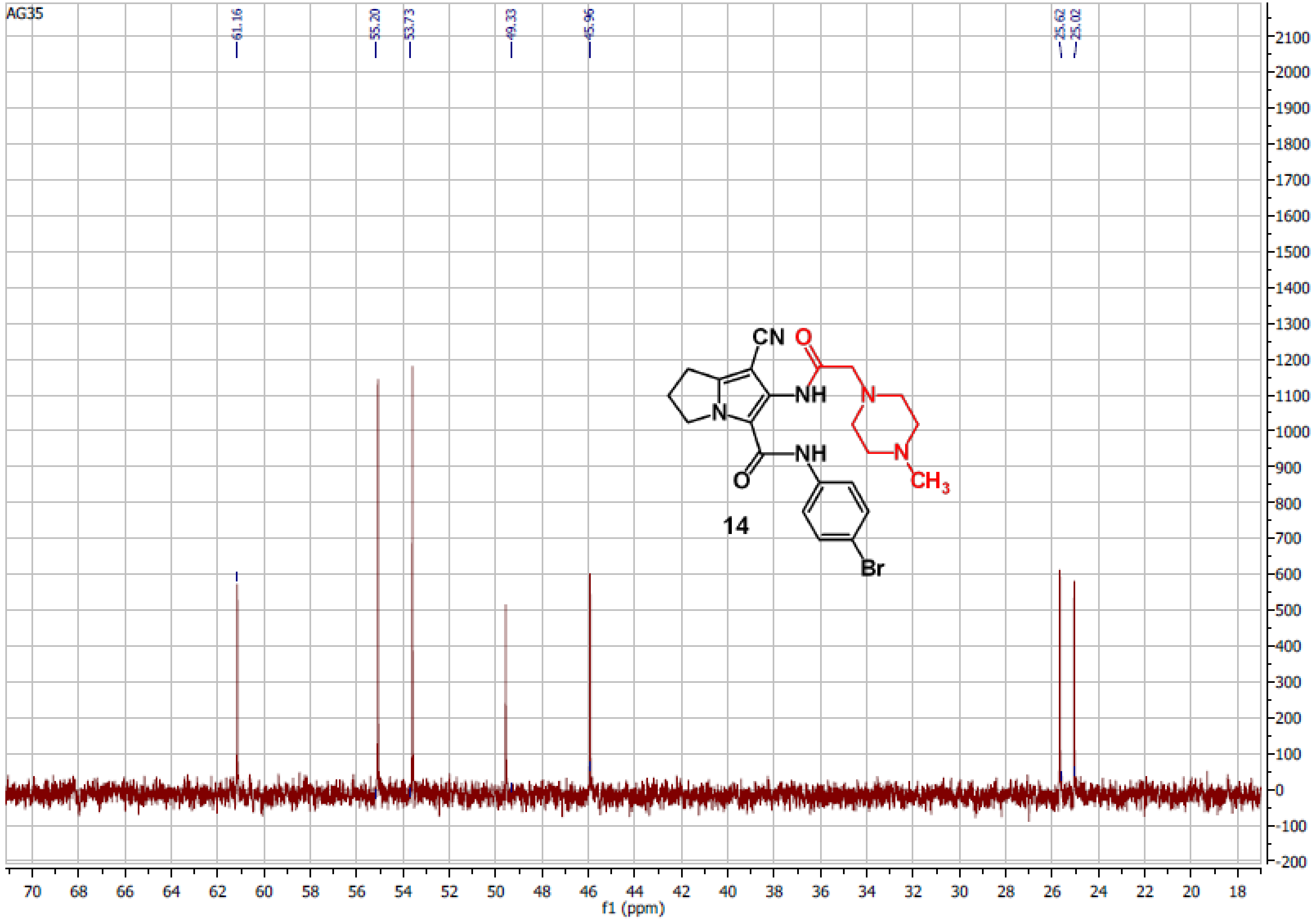

Figure S23b: 13C-NMR (CDCl3, 125 MHz, δ ppm) spectrum of compound 14 (ZOOM on Aliphatic Carbons)

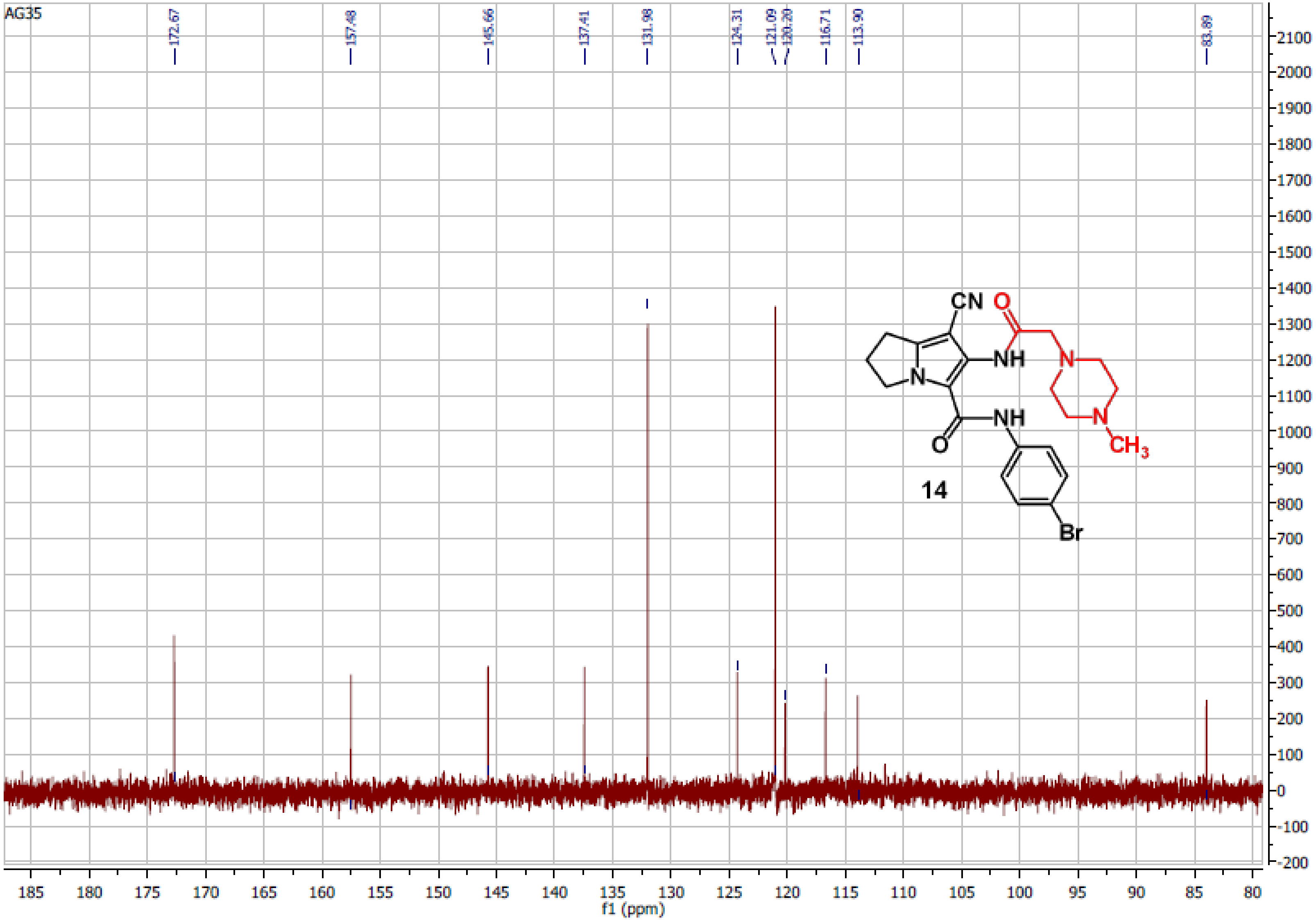

Figure S23c: 13C-NMR (CDCl3, 125 MHz, δ ppm) spectrum of compound 14 (ZOOM on Aromatic Carbons)

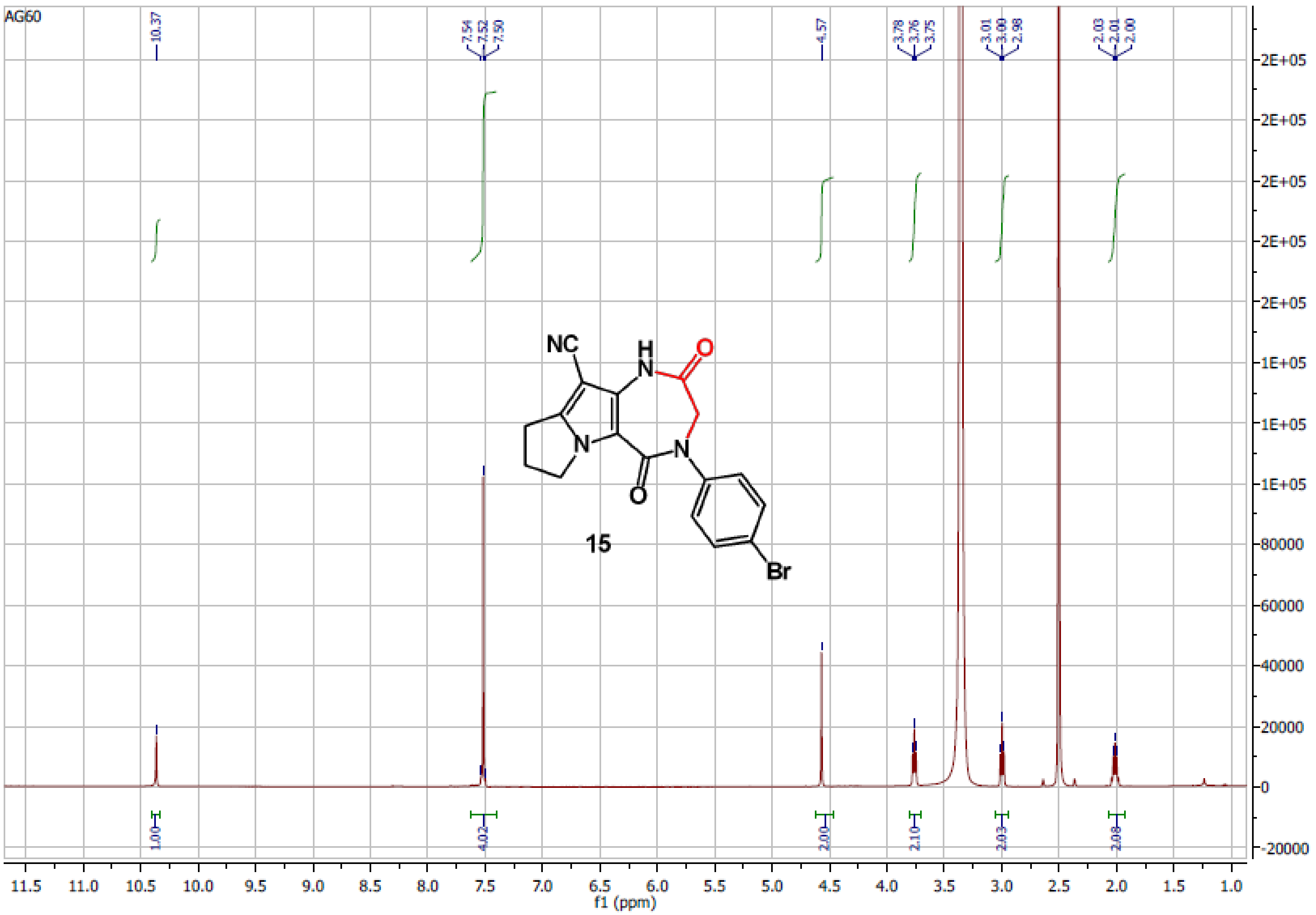

Figure S24a: 1H-NMR (DMSO, 500 MHz, δ ppm) spectrum of compound 15

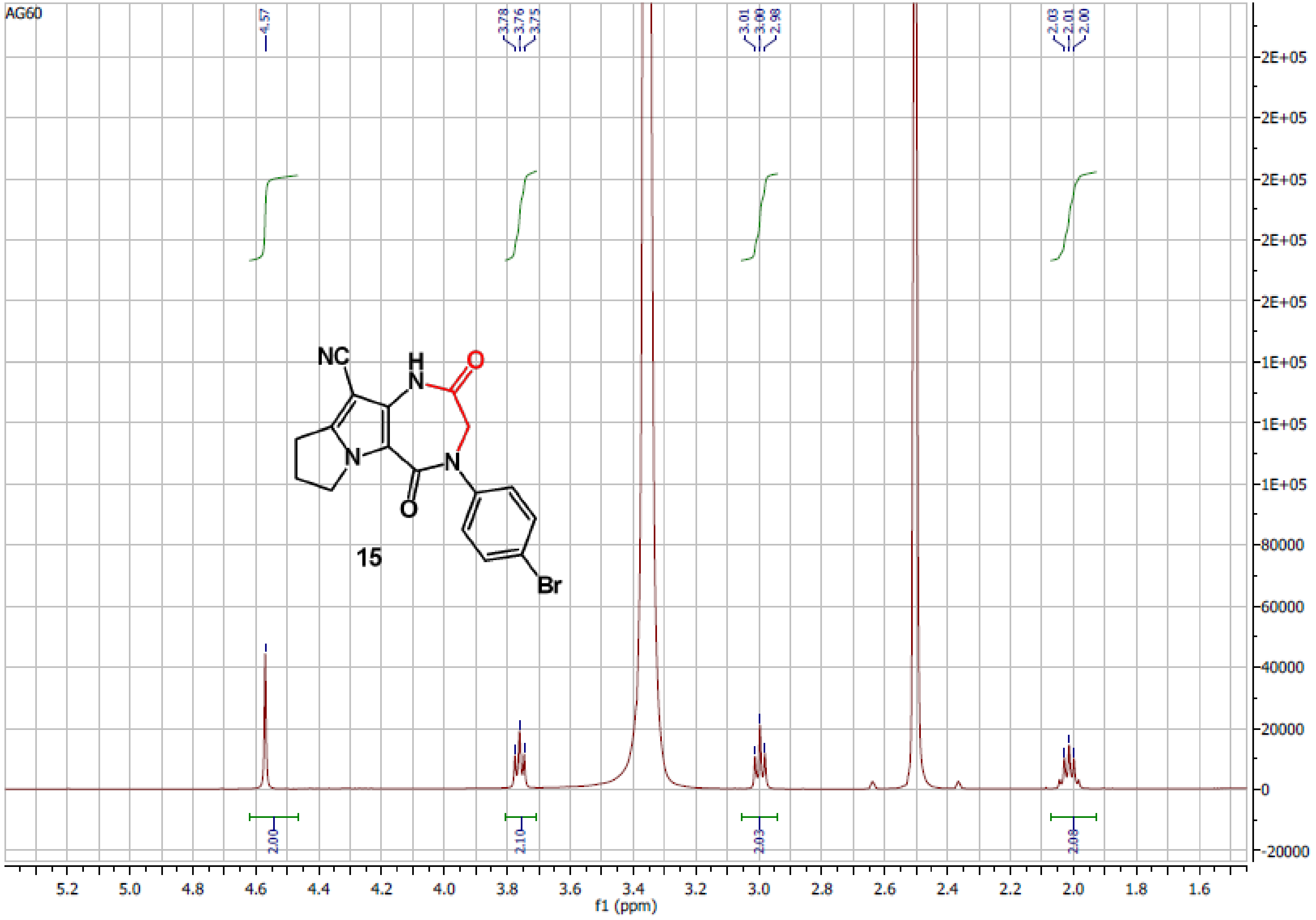

Figure S24b: 1H-NMR (DMSO, 500 MHz, δ ppm) spectrum of compound 15 (ZOOM on Aliphatic Protons)

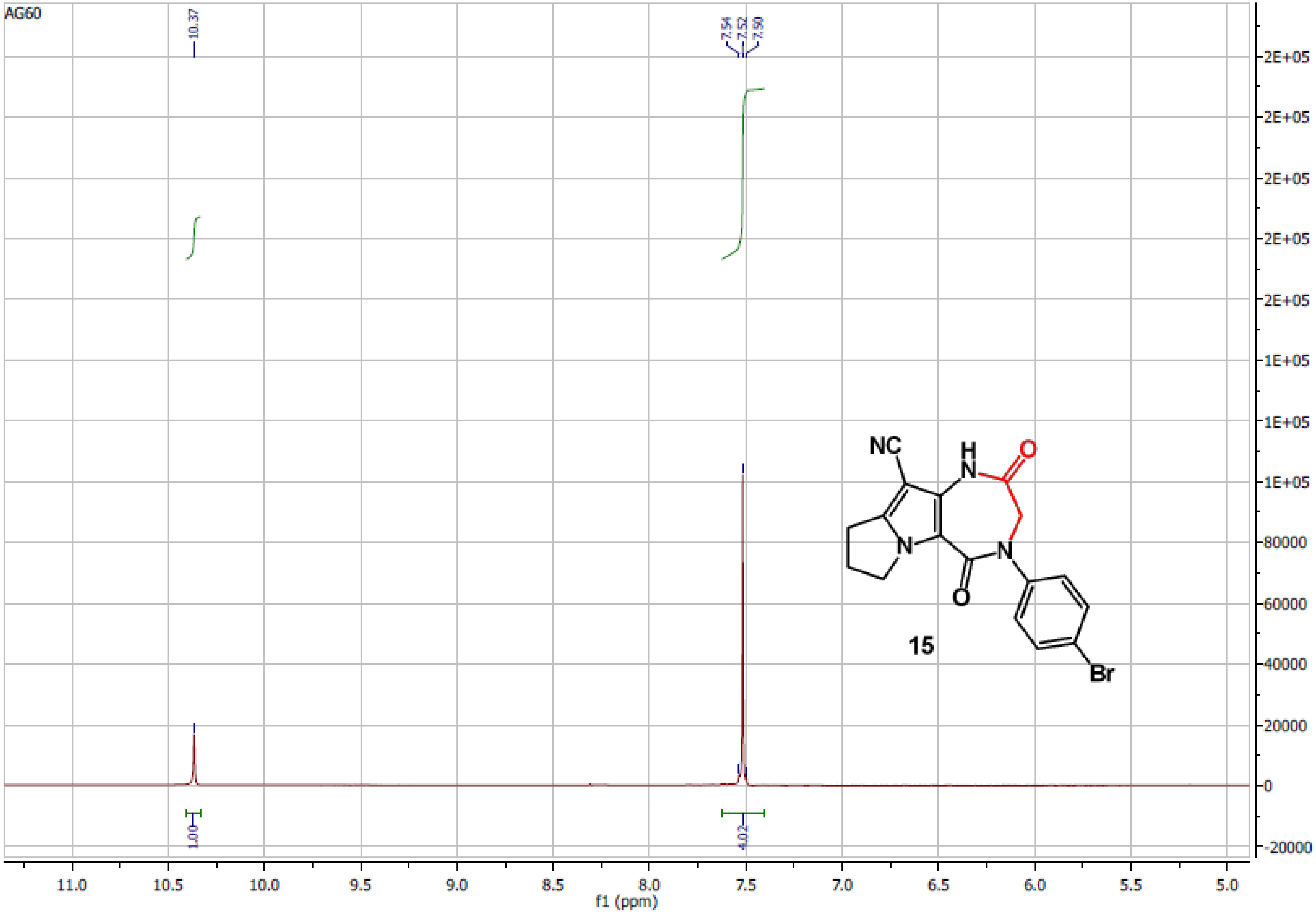

Figure S24c: 1H-NMR (DMSO, 500 MHz, δ ppm) spectrum of compound 15 (ZOOM on Aromatic Protons)

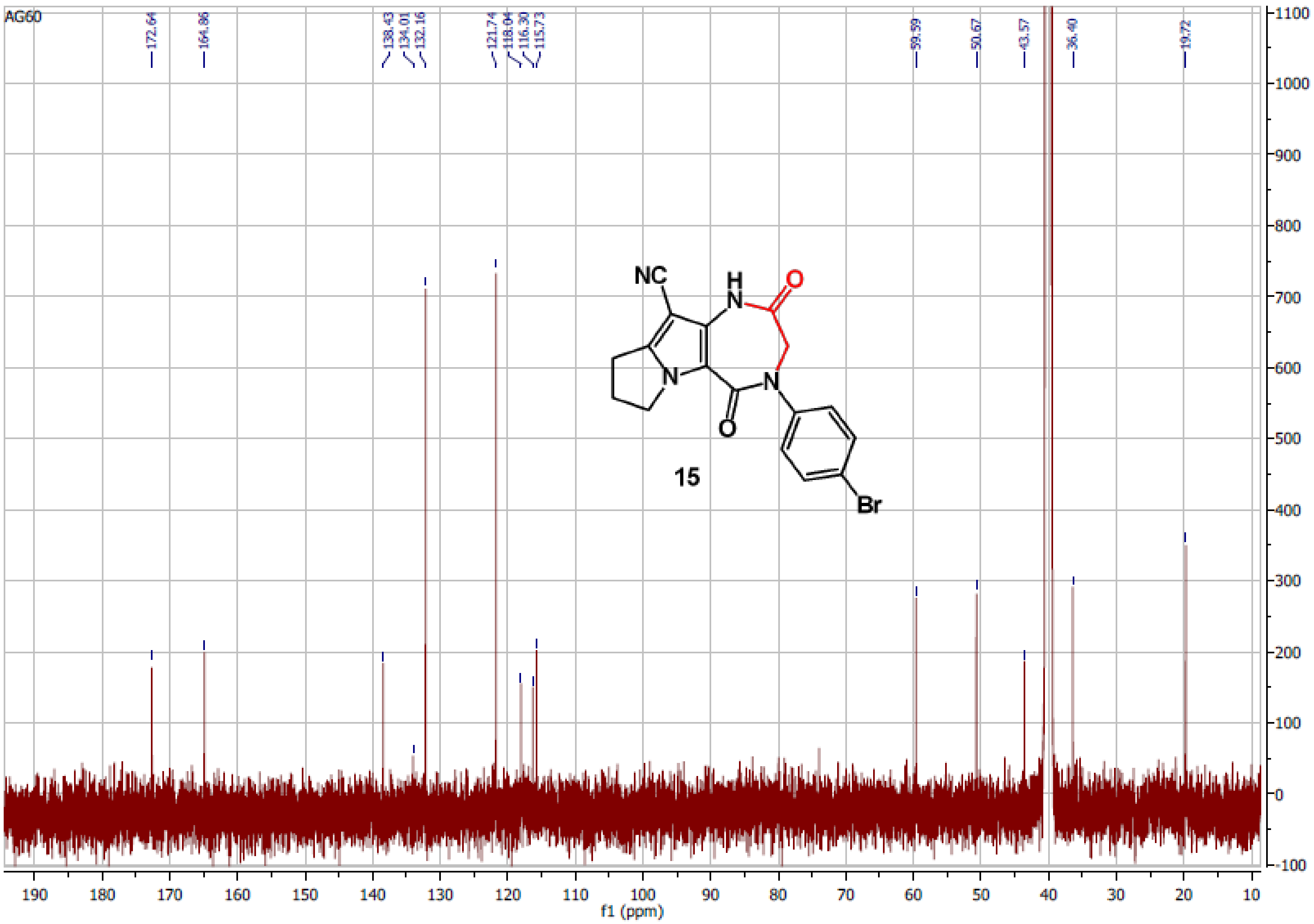

Figure S25a: 13C-NMR (DMSO, 125 MHz, δ ppm) spectrum of compound 15

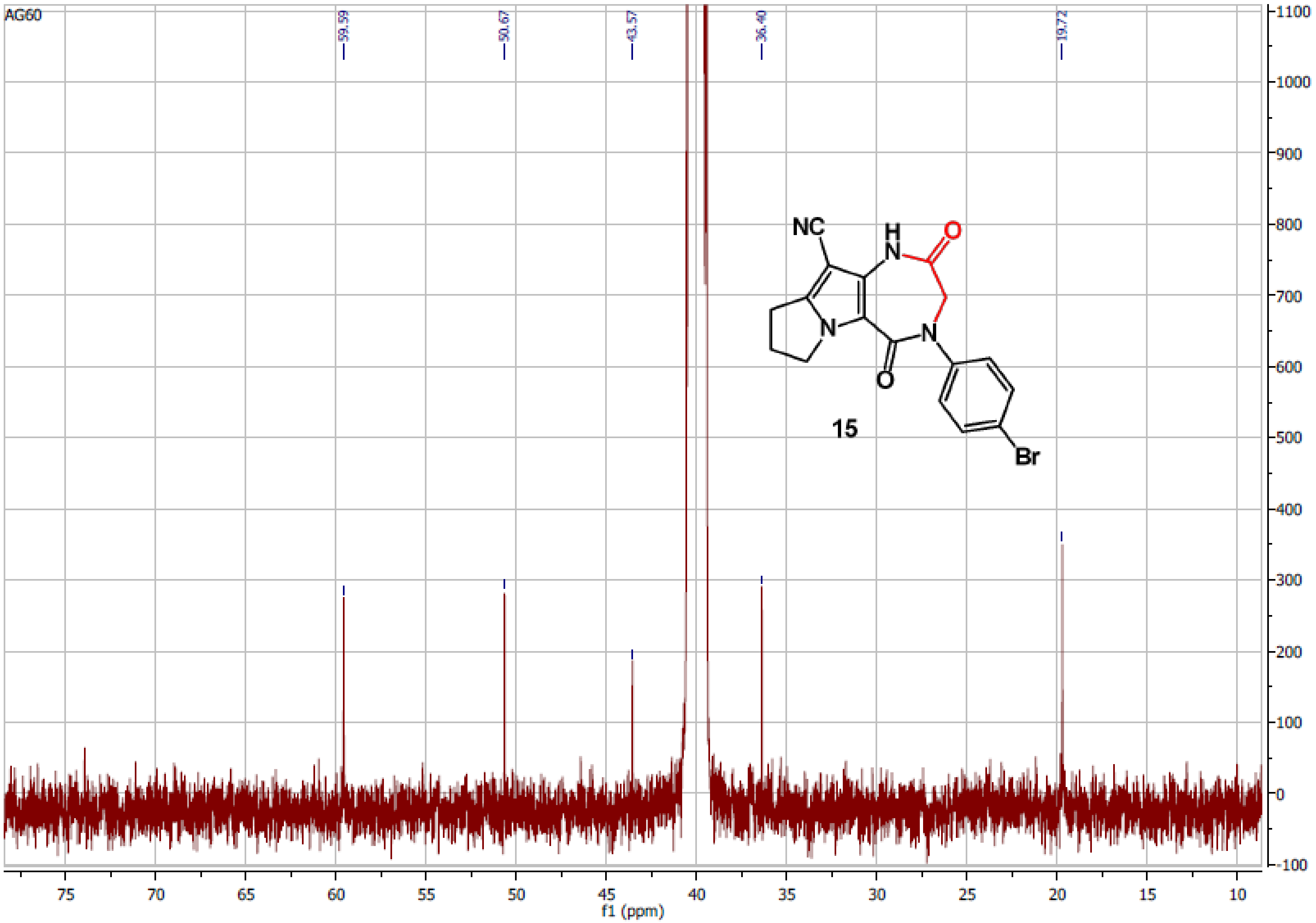

Figure S25b: 13C-NMR (DMSO, 125 MHz, δ ppm) spectrum of compound 15 (ZOOM on Aliphatic Carbons)

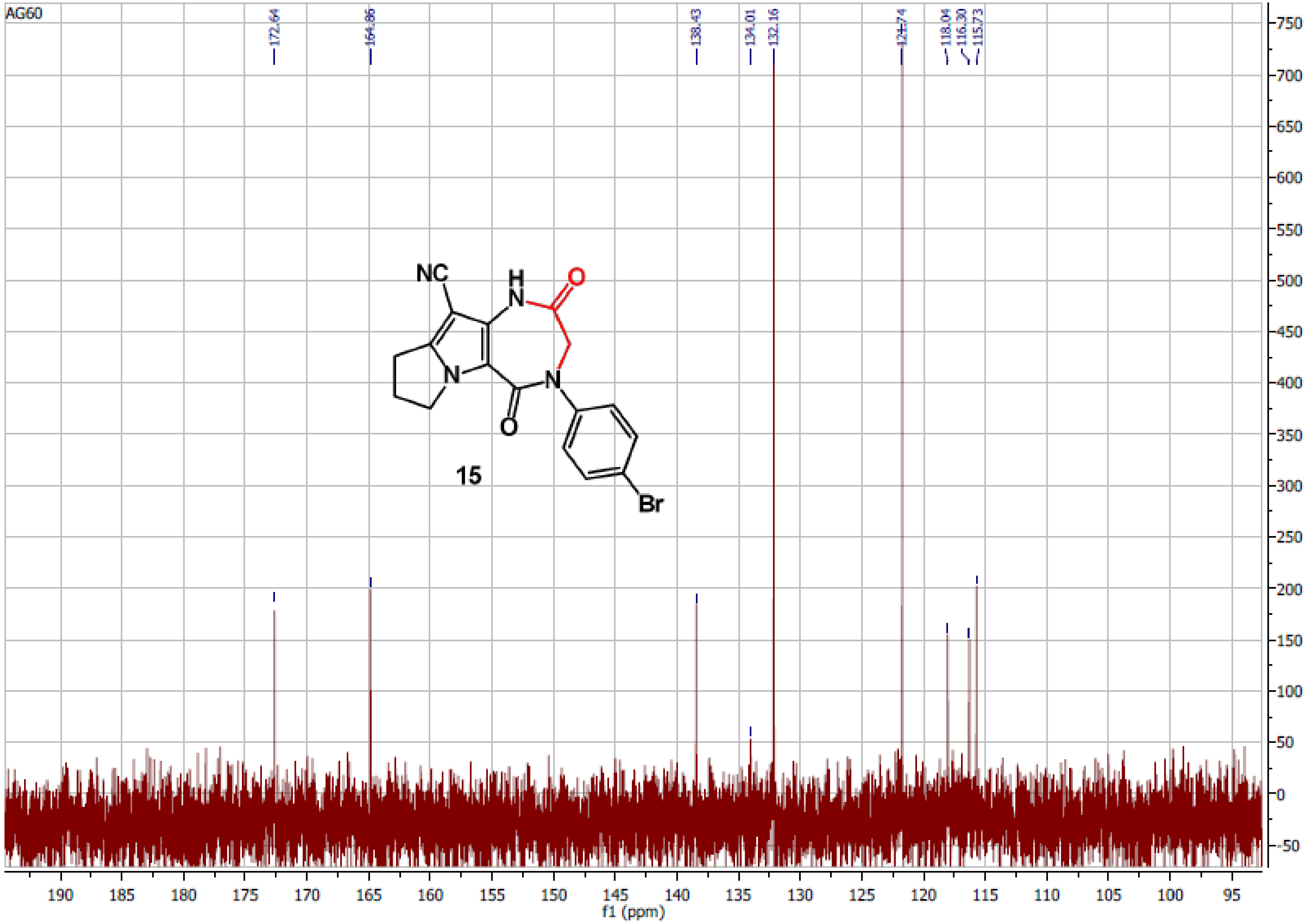

Figure S25c: 13C-NMR (DMSO, 125 MHz, δ ppm) spectrum of compound 15 (ZOOM on Aromatic Carbons)

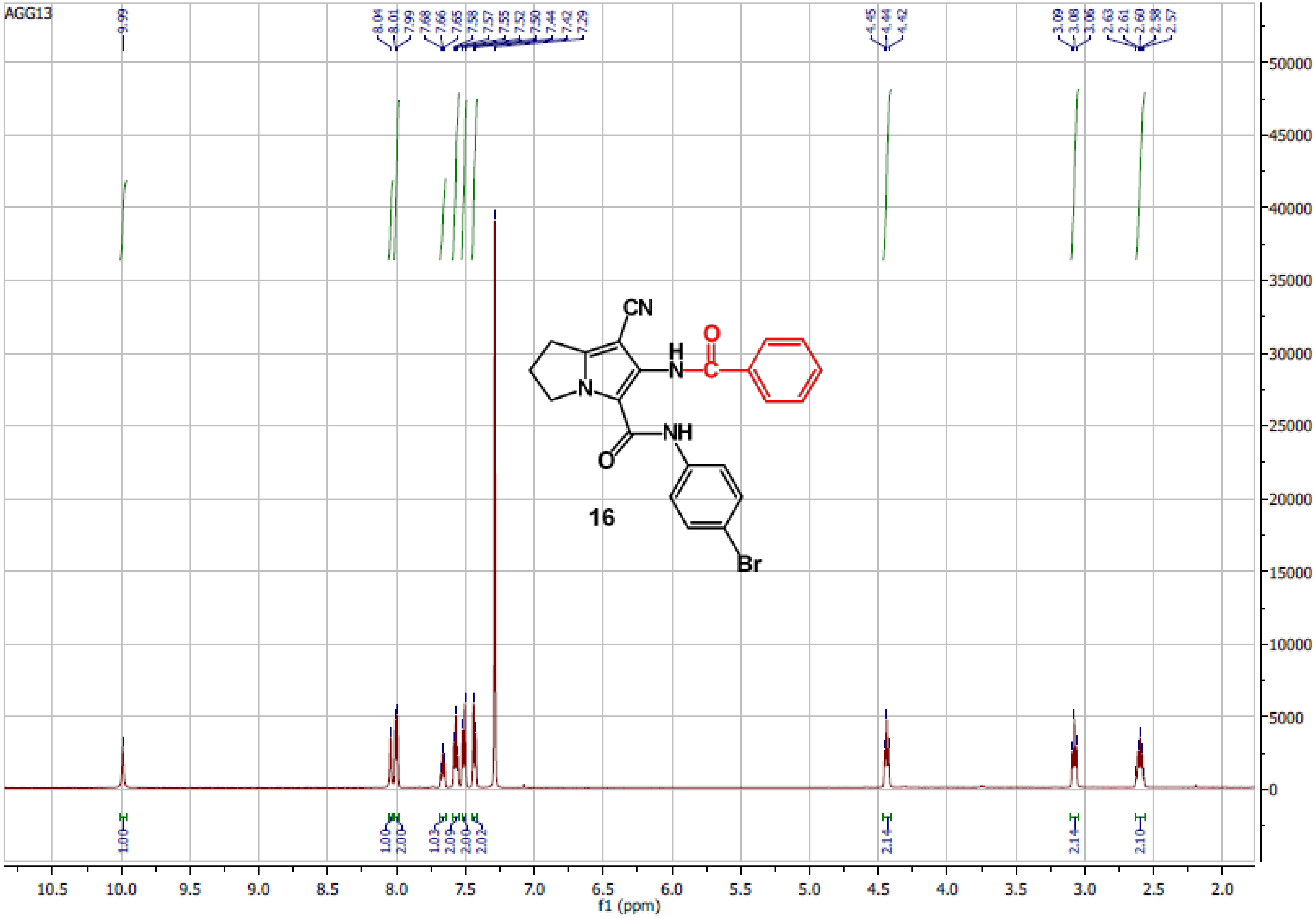

Figure S26a: 1H-NMR (CDCl3, 500 MHz, δ ppm) spectrum of compound 16

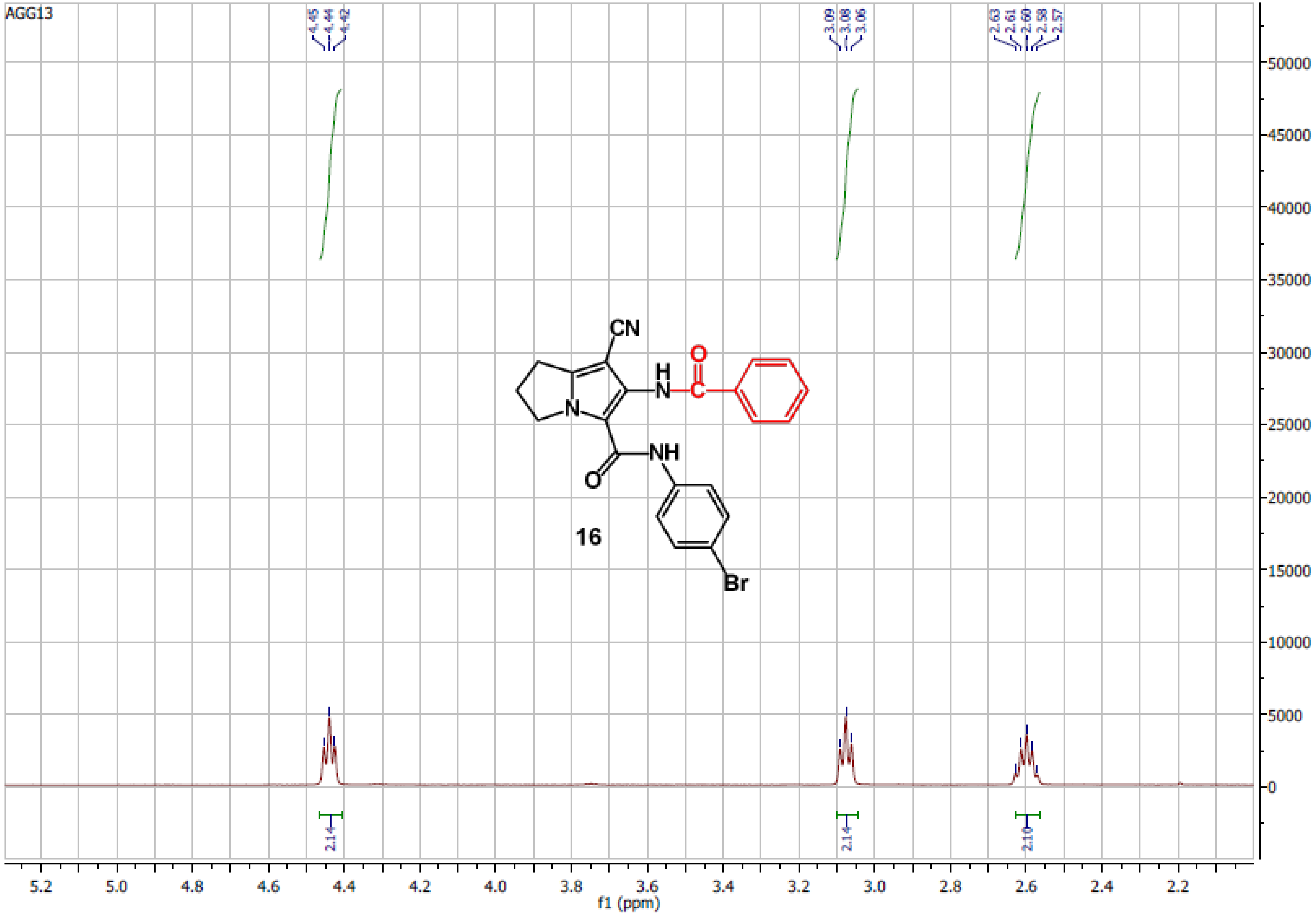

Figure S26b: 1H-NMR (CDCl3, 500 MHz, δ ppm) spectrum of compound 16 (ZOOM on Aliphatic Protons)

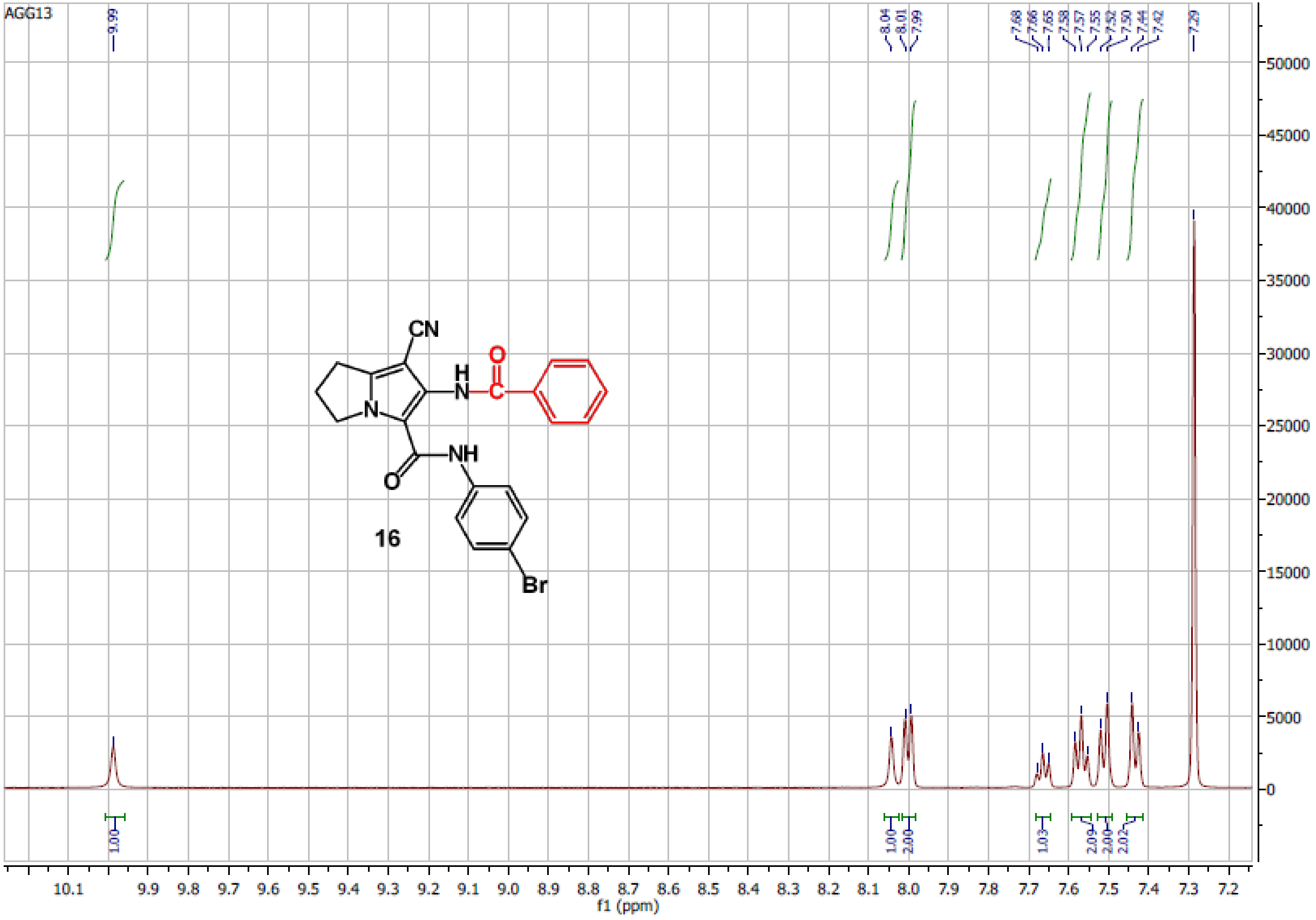

Figure S26c: 1H-NMR (CDCl3, 500 MHz, δ ppm) spectrum of compound 16 (ZOOM on Aromatic Protons)

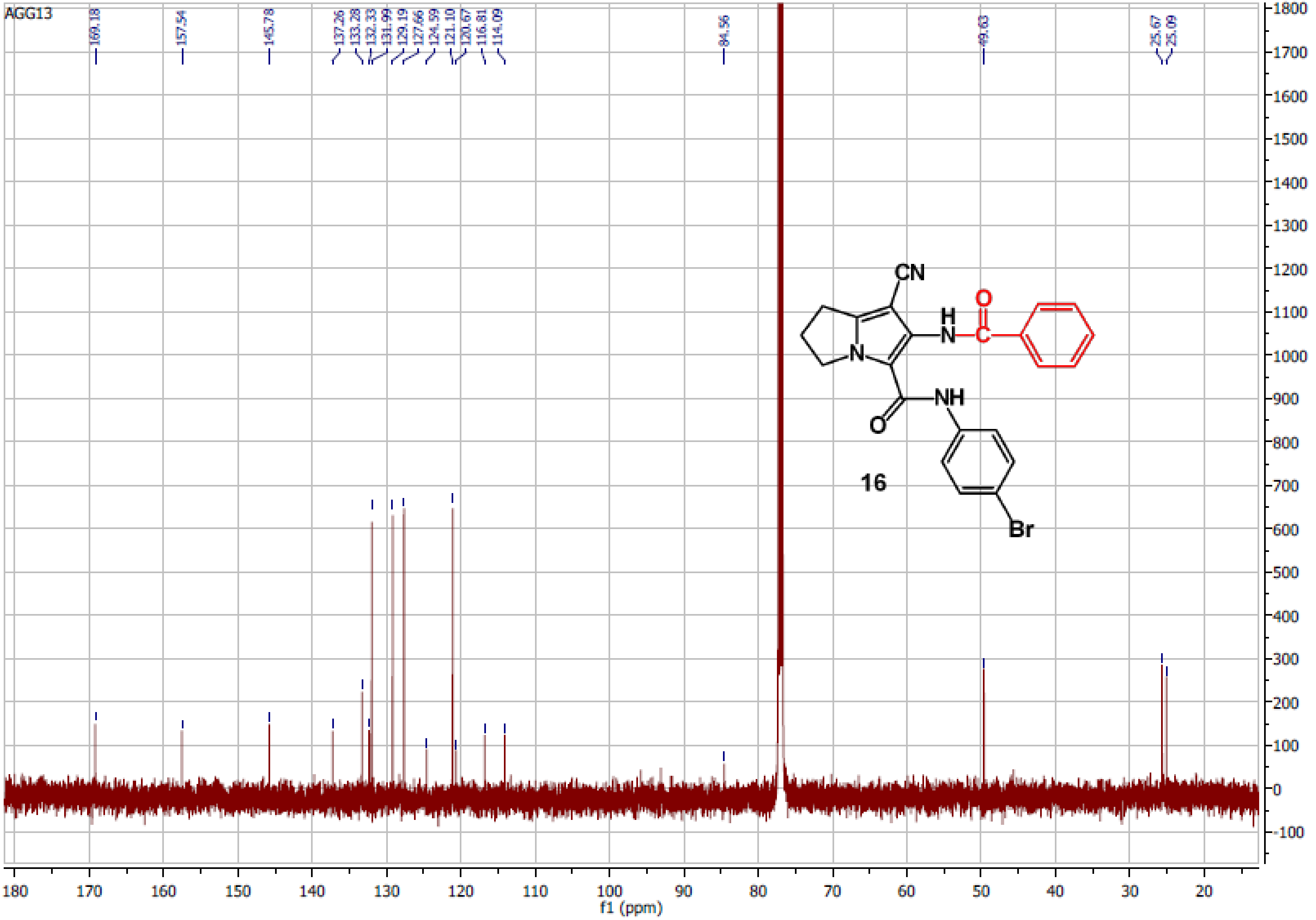

Figure S27a: 13C-NMR (CDCl3, 125 MHz, δ ppm) spectrum of compound 16

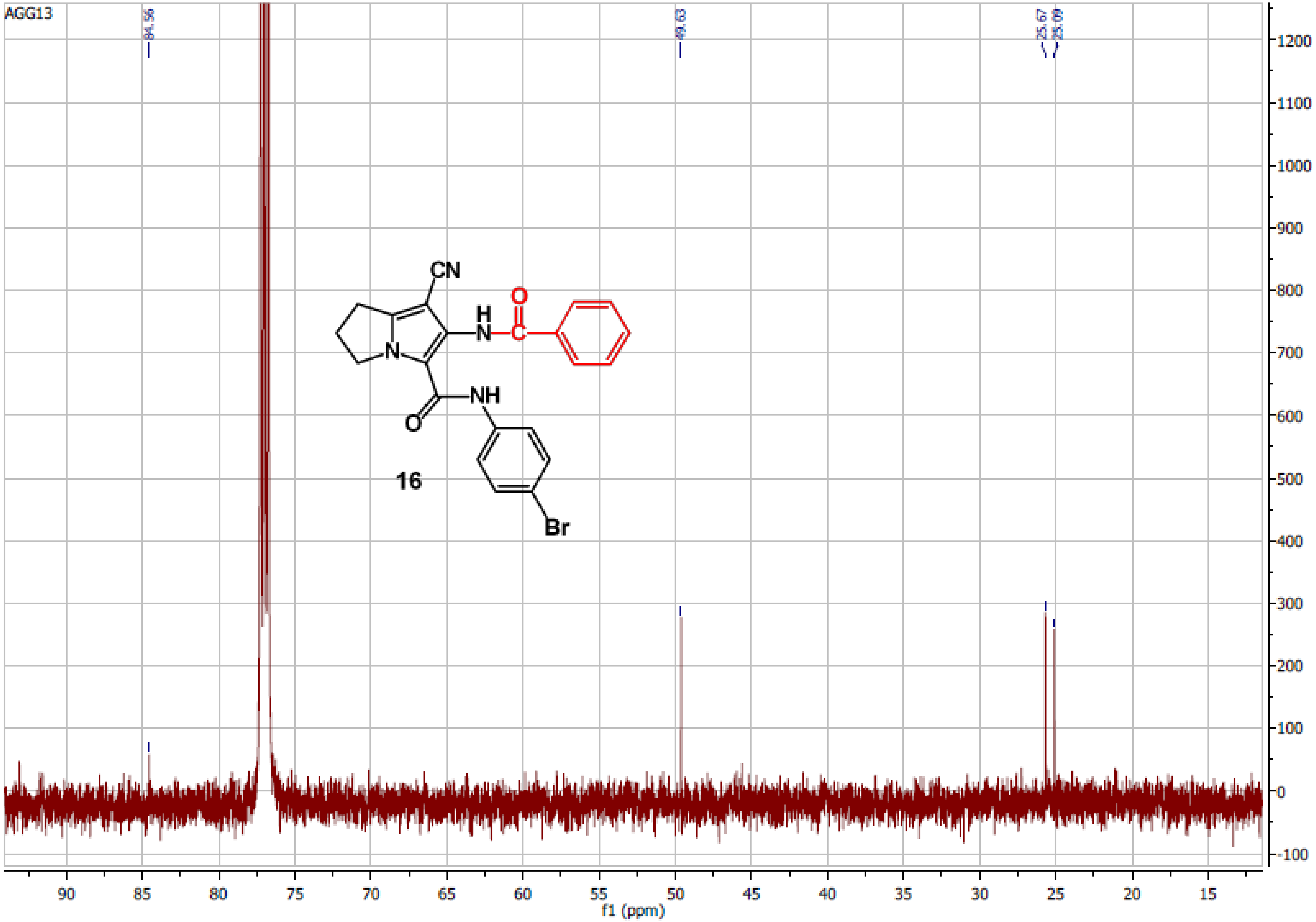

Figure S27b: 13C-NMR (CDCl3, 125 MHz, δ ppm) spectrum of compound 16 (ZOOM on Aliphatic Carbons)

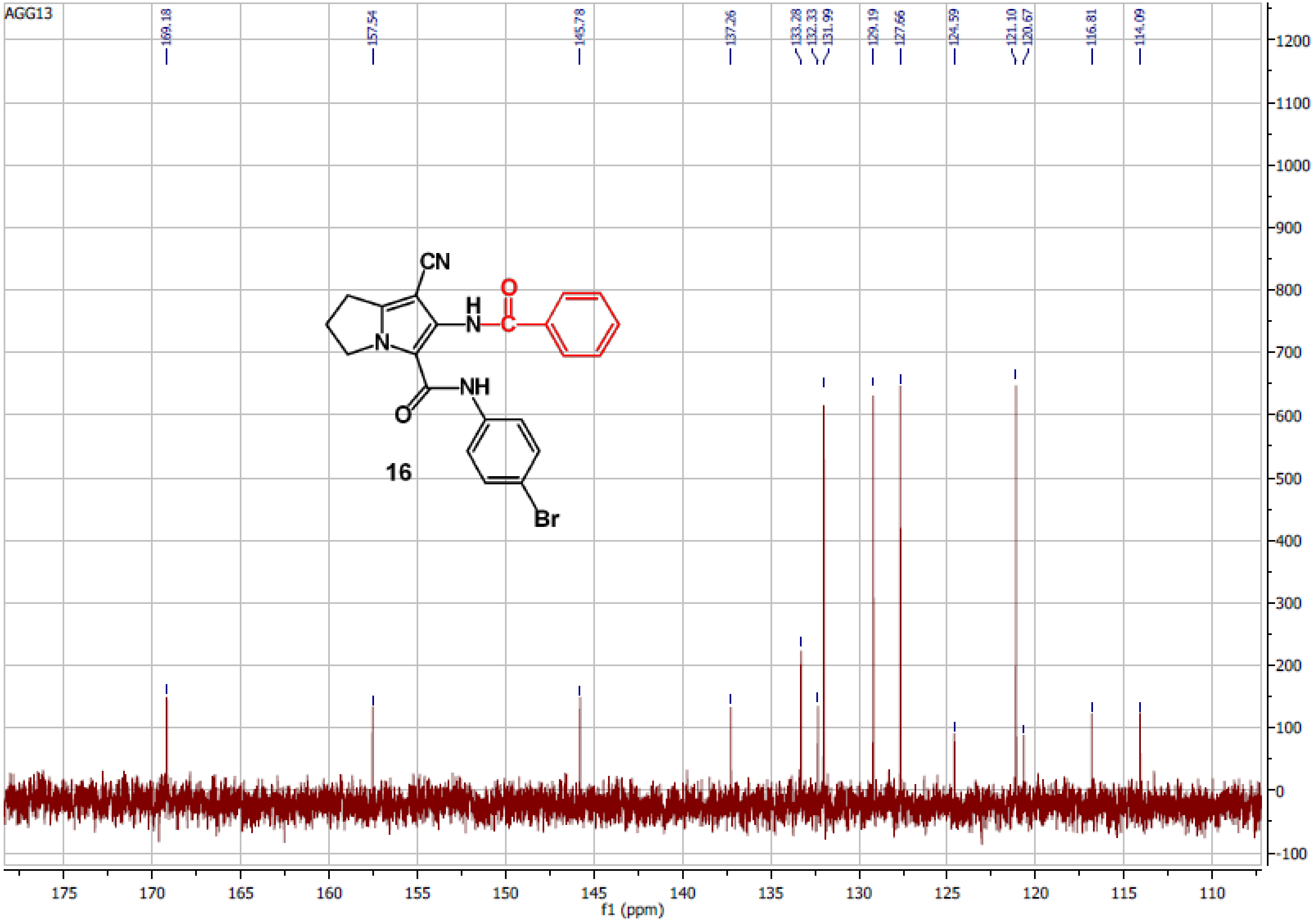

Figure S27c: 13C-NMR (CDCl3, 125 MHz, δ ppm): spectrum of compound 16 (ZOOM on Aromatic Carbons)

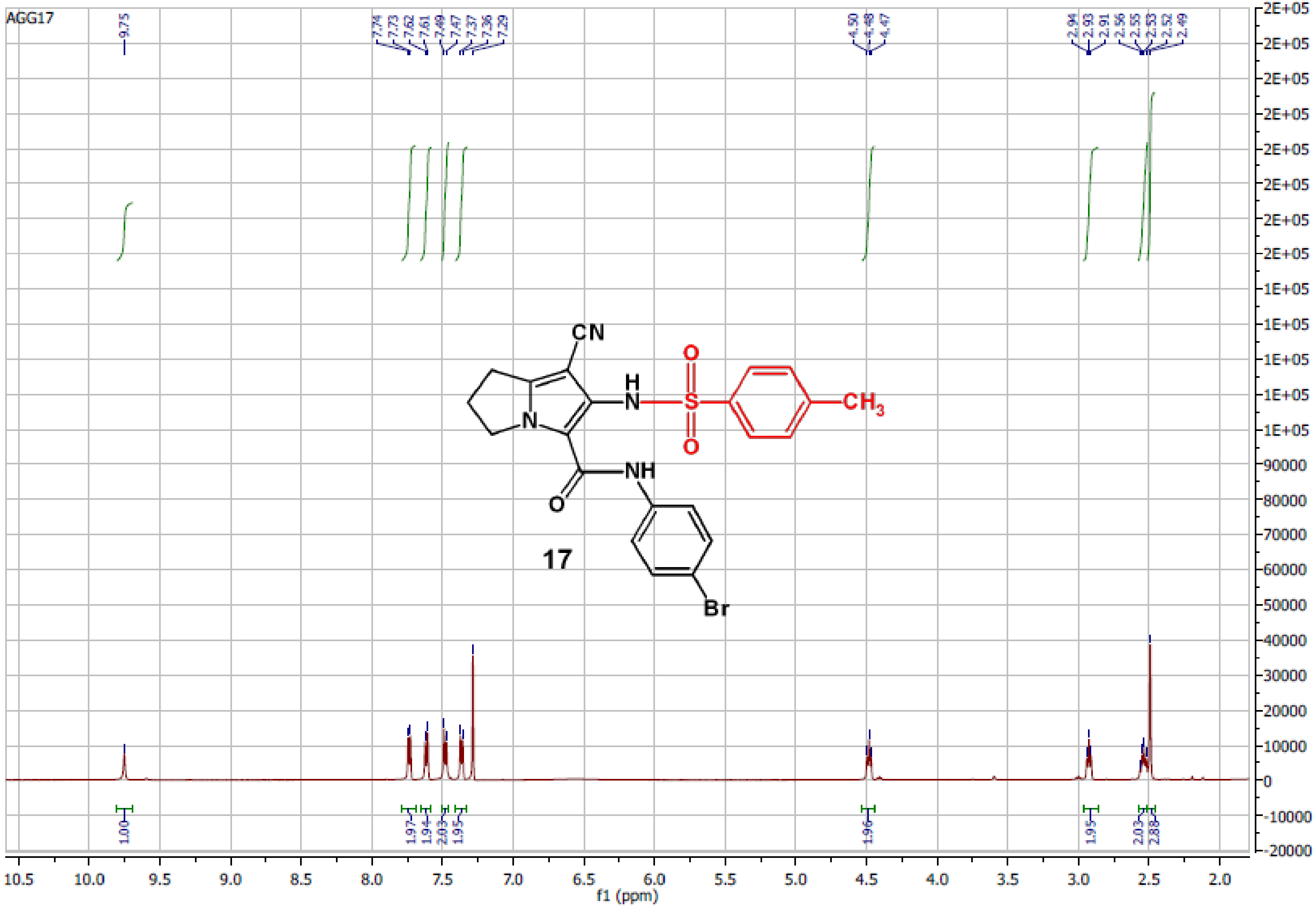

Figure S28a: 1H-NMR (CDCl3, 500 MHz, δ ppm) spectrum of compound 17

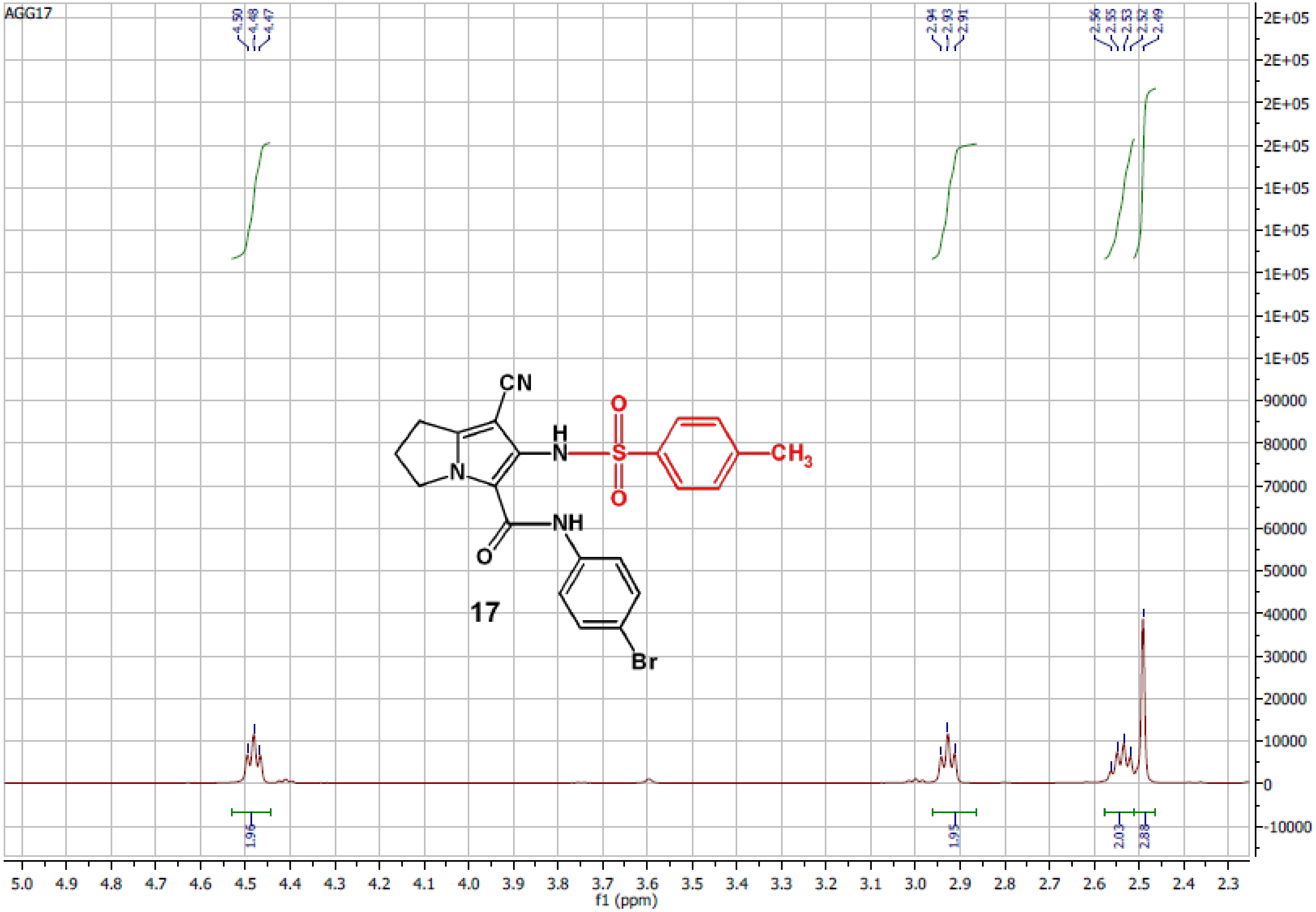

Figure S28b: 1H-NMR (CDCl3, 500 MHz, δ ppm) spectrum of compound 17 (ZOON on Aliphatic Protons)

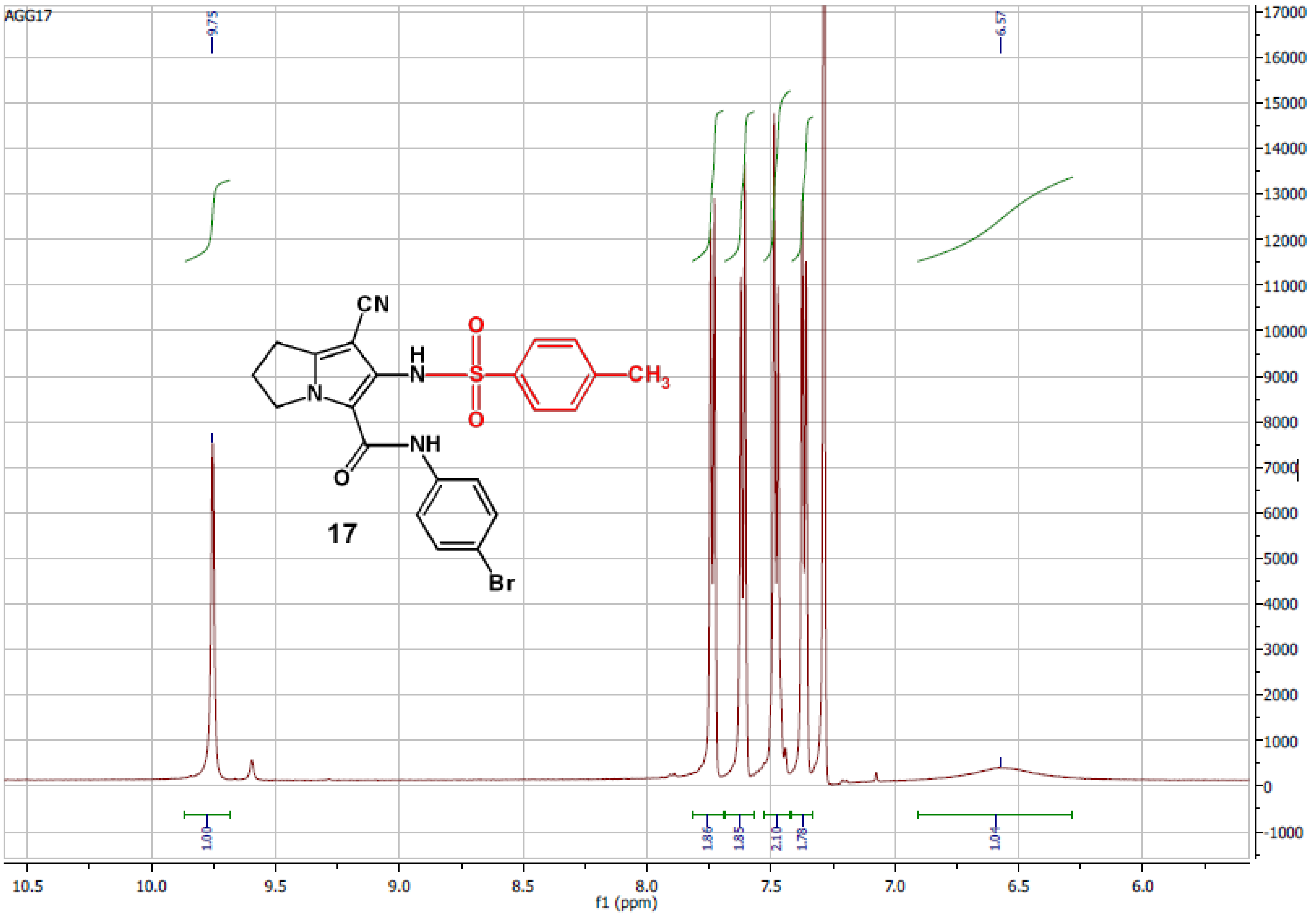

Figure S28c: 1H-NMR (CDCl3, 500 MHz, δ ppm) spectrum of compound 17 (ZOON on NHs + Aromatic Protons)

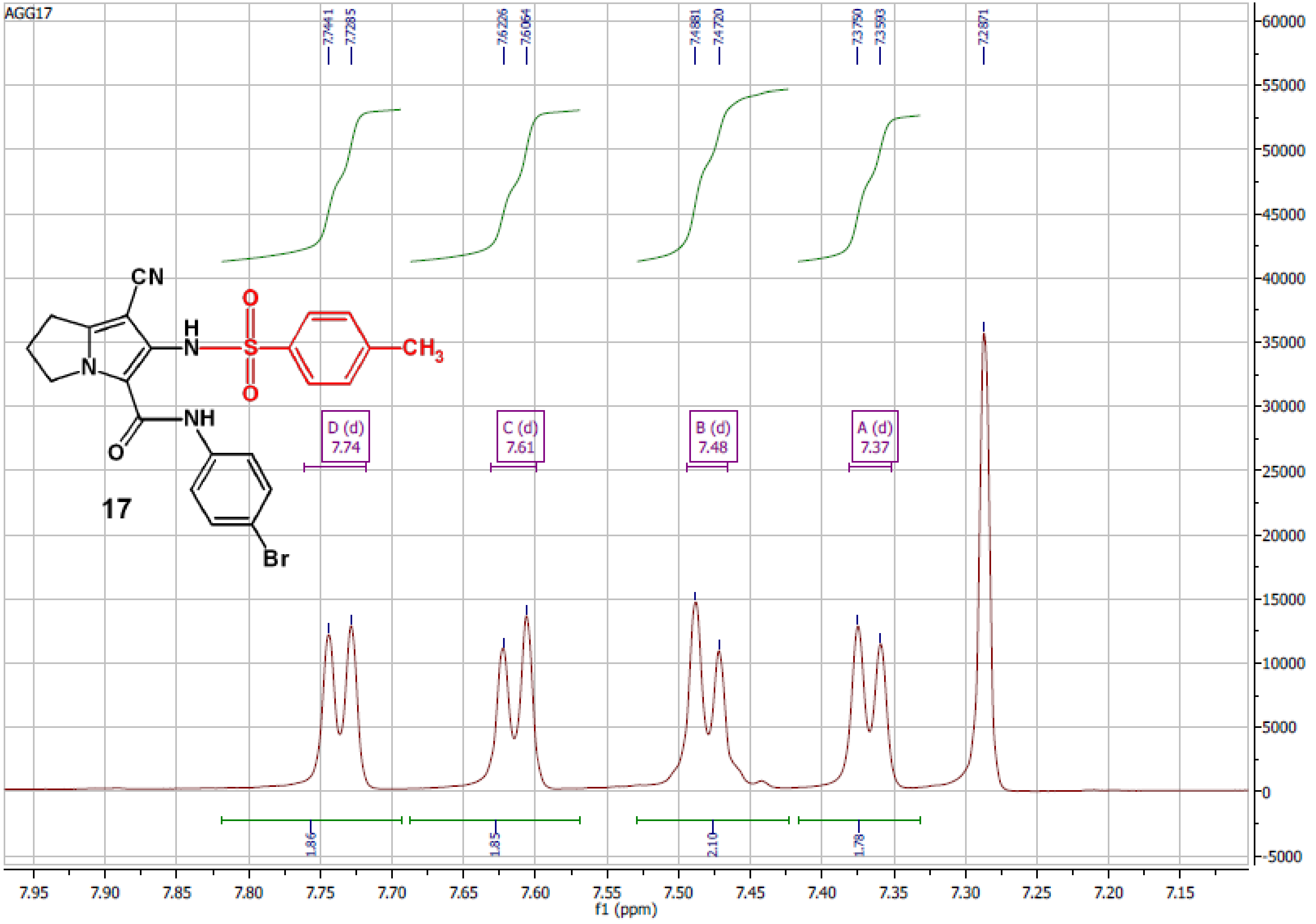

Figure S28d: 1H-NMR (CDCl3, 500 MHz, δ ppm) spectrum of compound 17 (ZOON on Aromatic Protons)

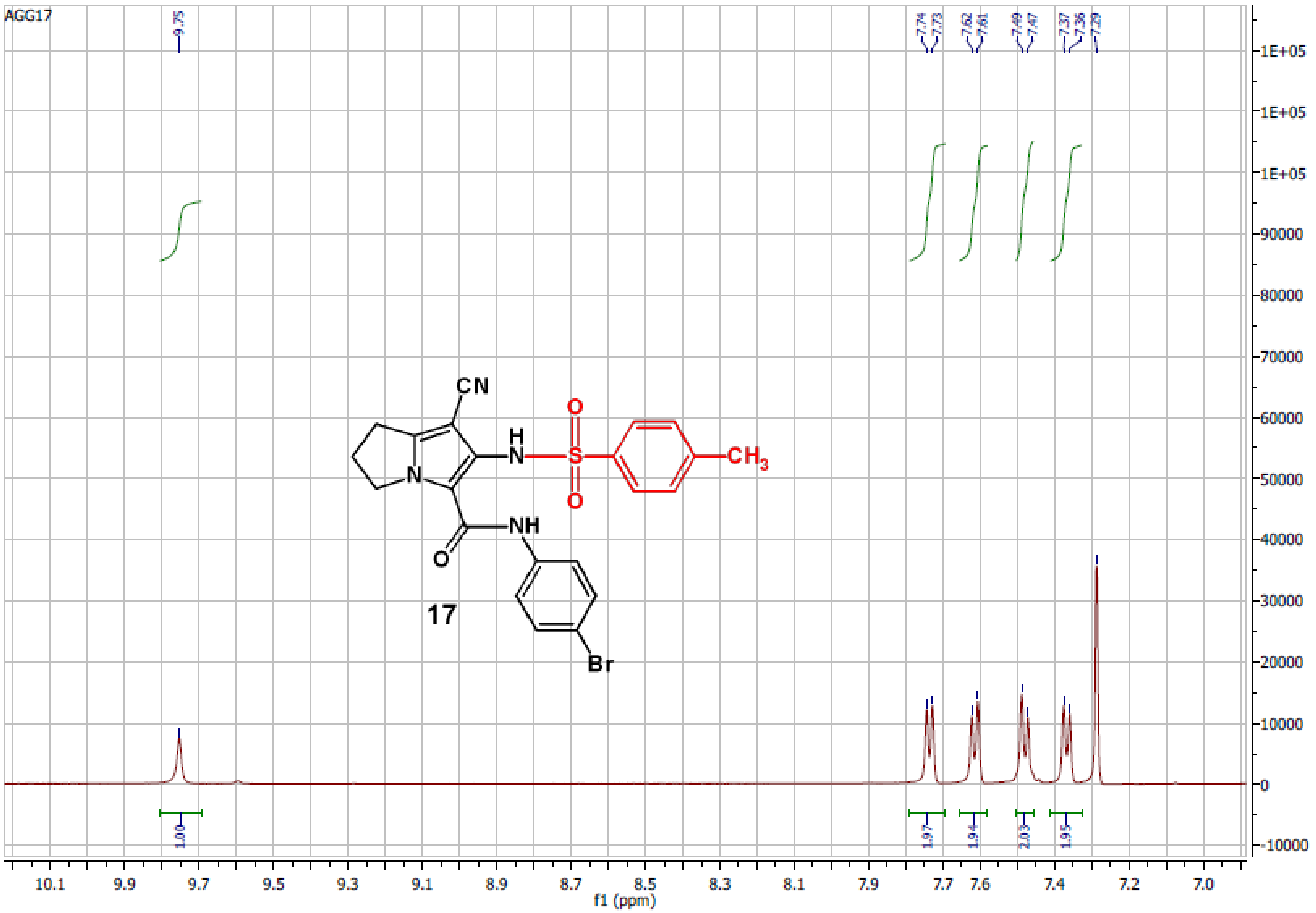

Figure S28e: 1H-NMR (CDCl3, 500 MHz, δ ppm) spectrum of compound 17 (ZOON on Aromatic Protons)

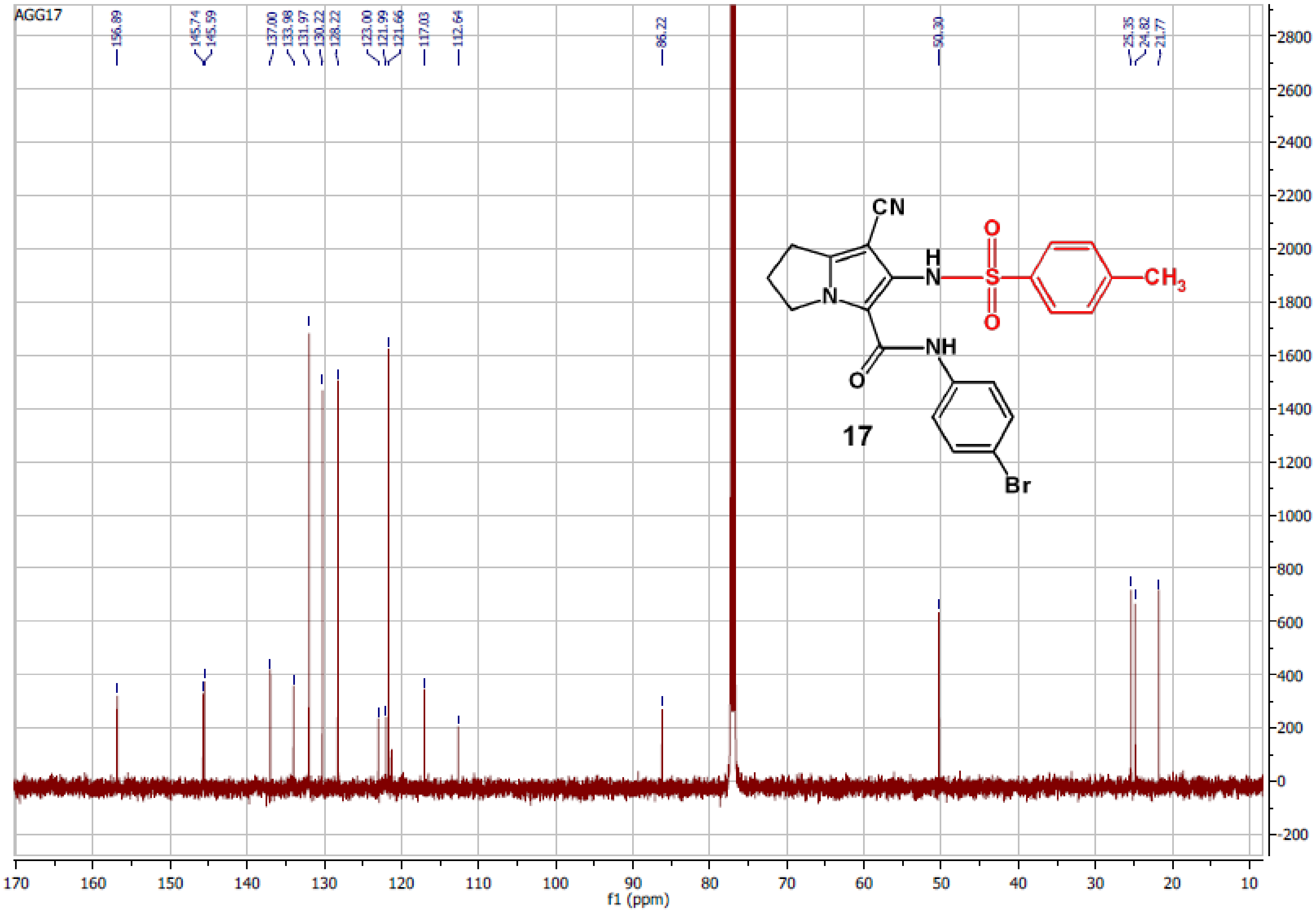

Figure S29a: 13C-NMR (CDCl3, 125 MHz, δ ppm) spectrum of compound 17

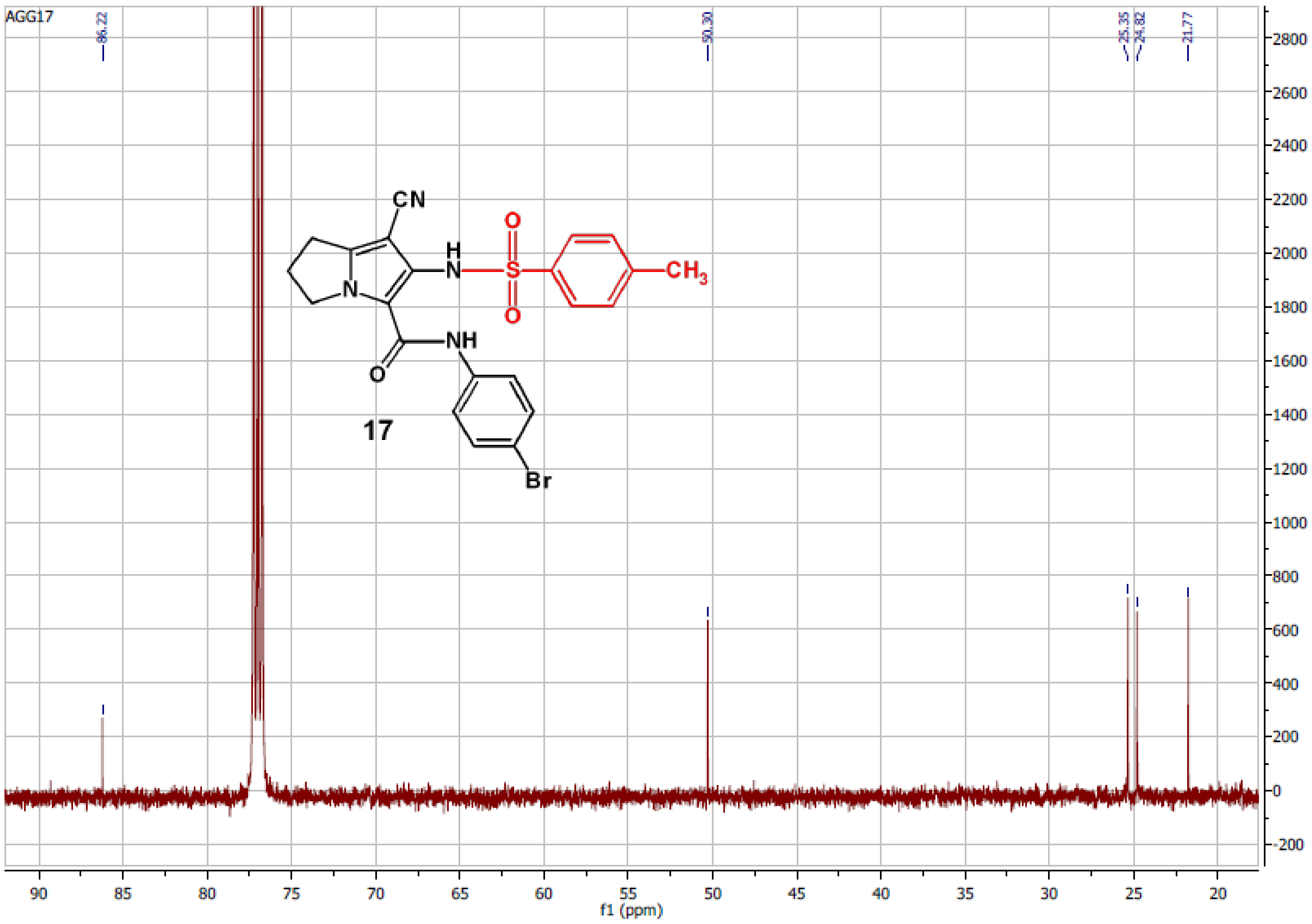

Figure S29b: 13C-NMR (CDCl3, 125 MHz, δ ppm) spectrum of compound 17 (ZOOM on Aliphatic Carbons)

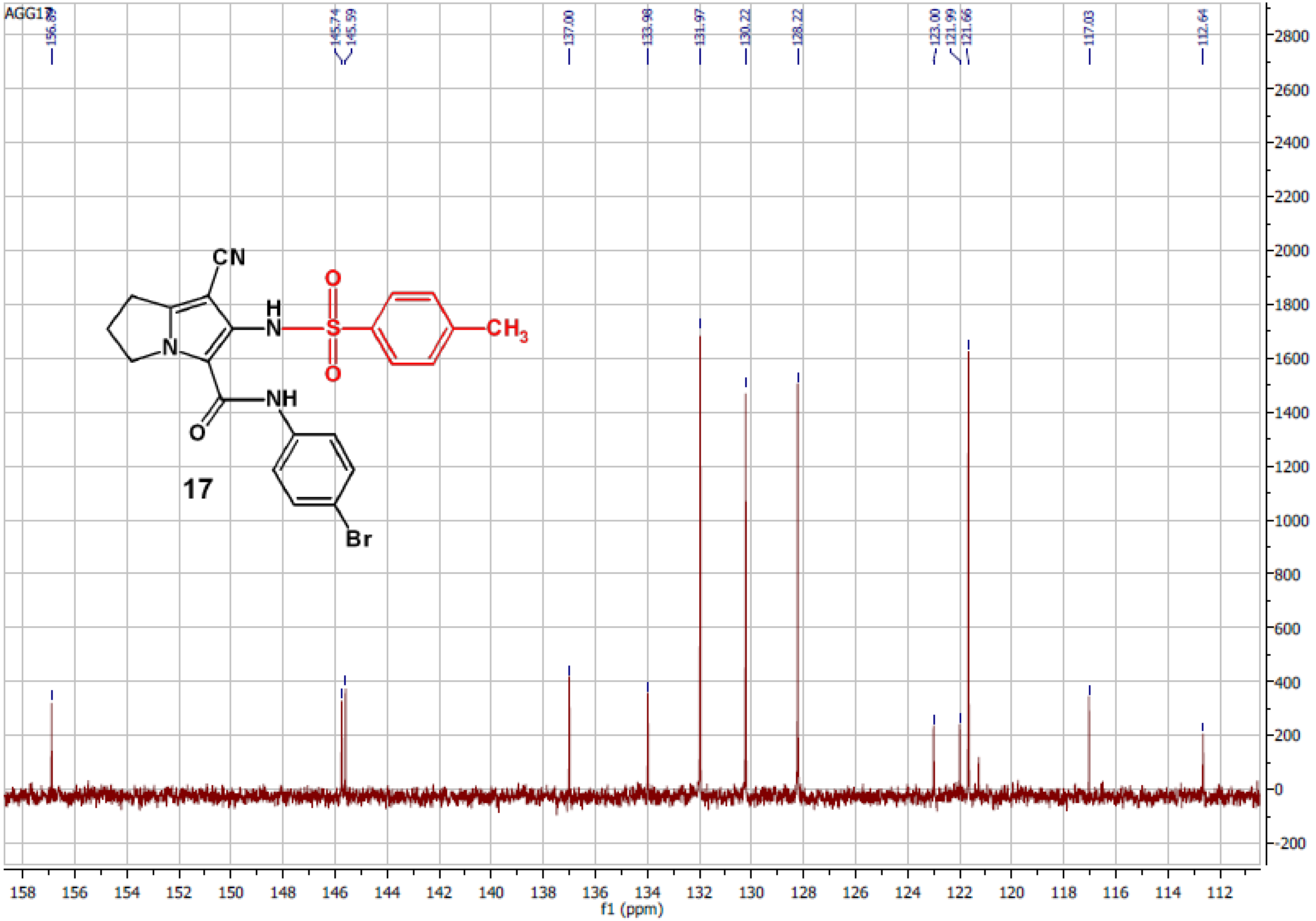

Figure S29c: 13C-NMR (CDCl3, 125 MHz, δ ppm) spectrum of compound 17 (ZOOM on Aromatic Carbons)

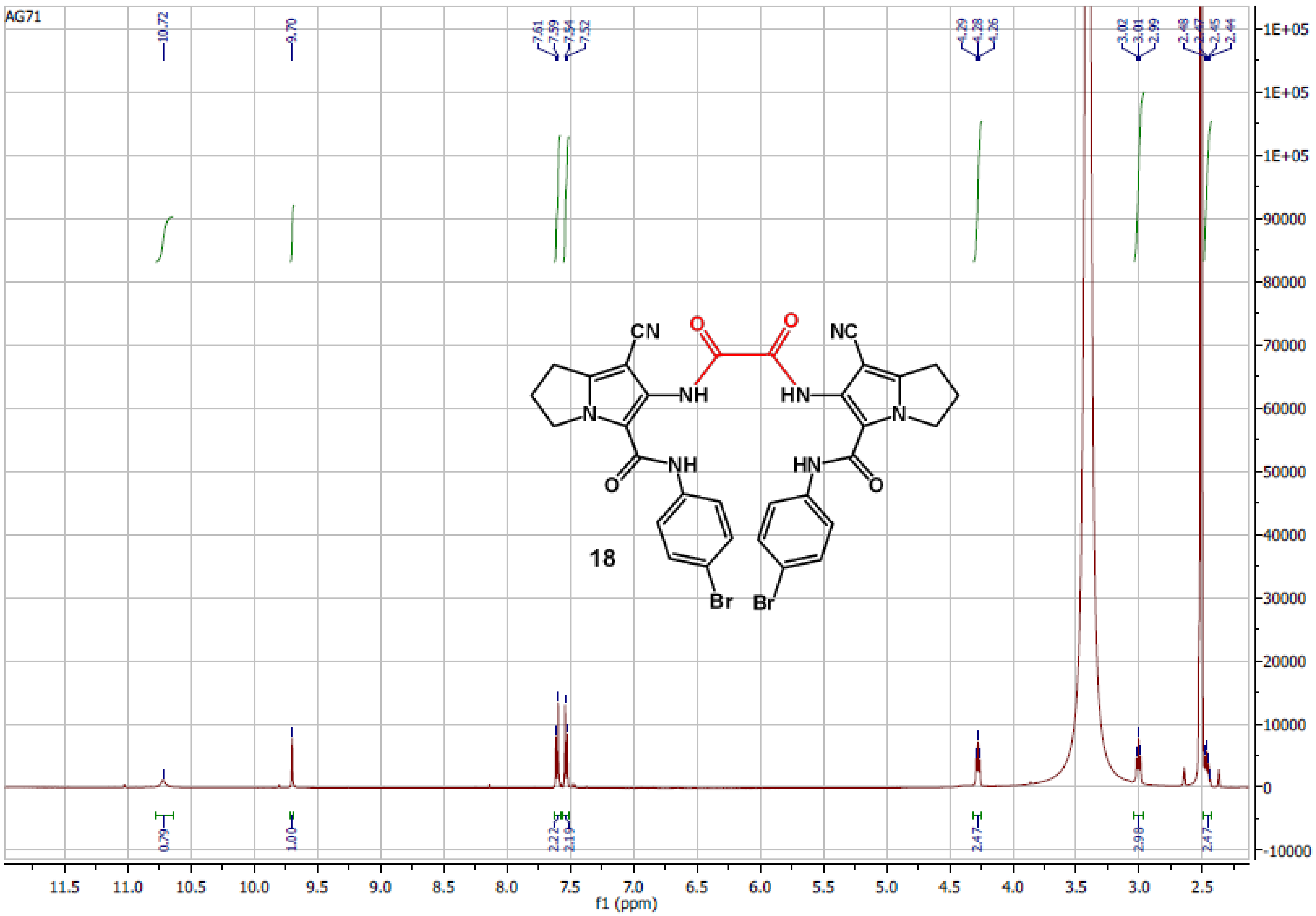

Figure S30a: 1H-NMR (DMSO, 500 MHz, δ ppm) spectrum of compound 18

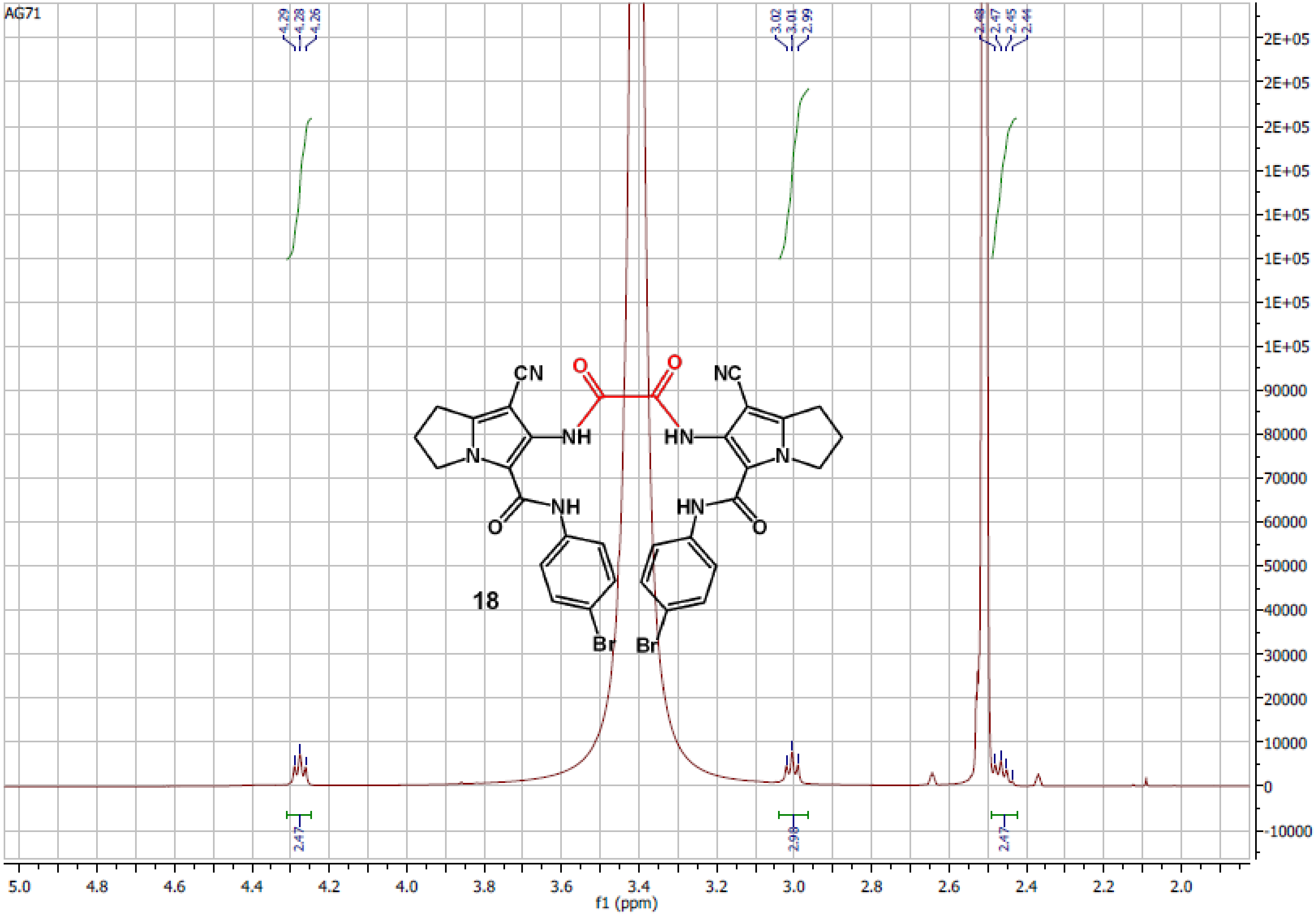

Figure S30b: 1H-NMR (DMSO, 500 MHz, δ ppm) spectrum of compound 18 (ZOOM on Aliphatic Protons)

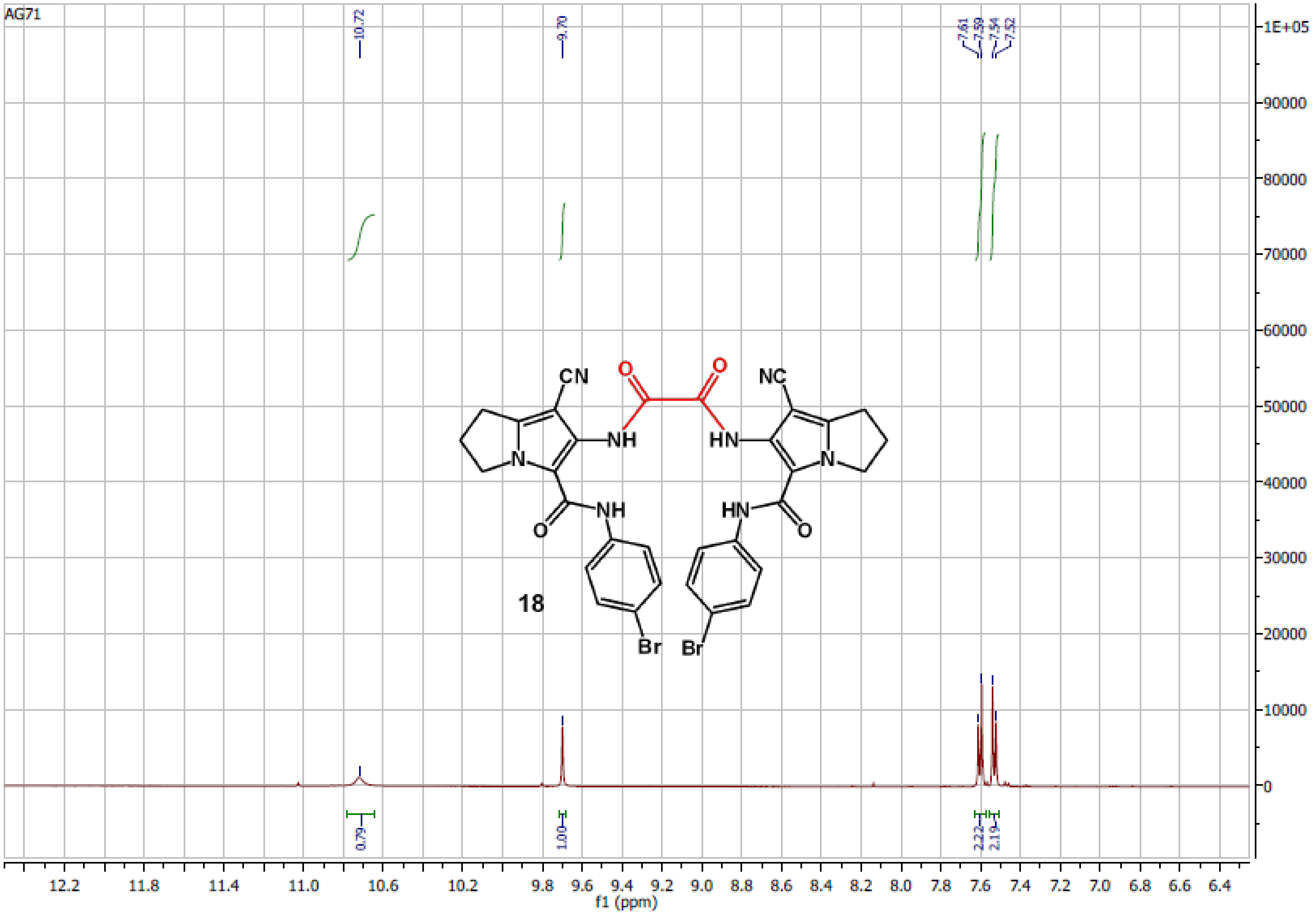

Figure S30c: 1H-NMR (DMSO, 500 MHz, δ ppm) spectrum of compound 18 (ZOOM on Aromatic Protons)

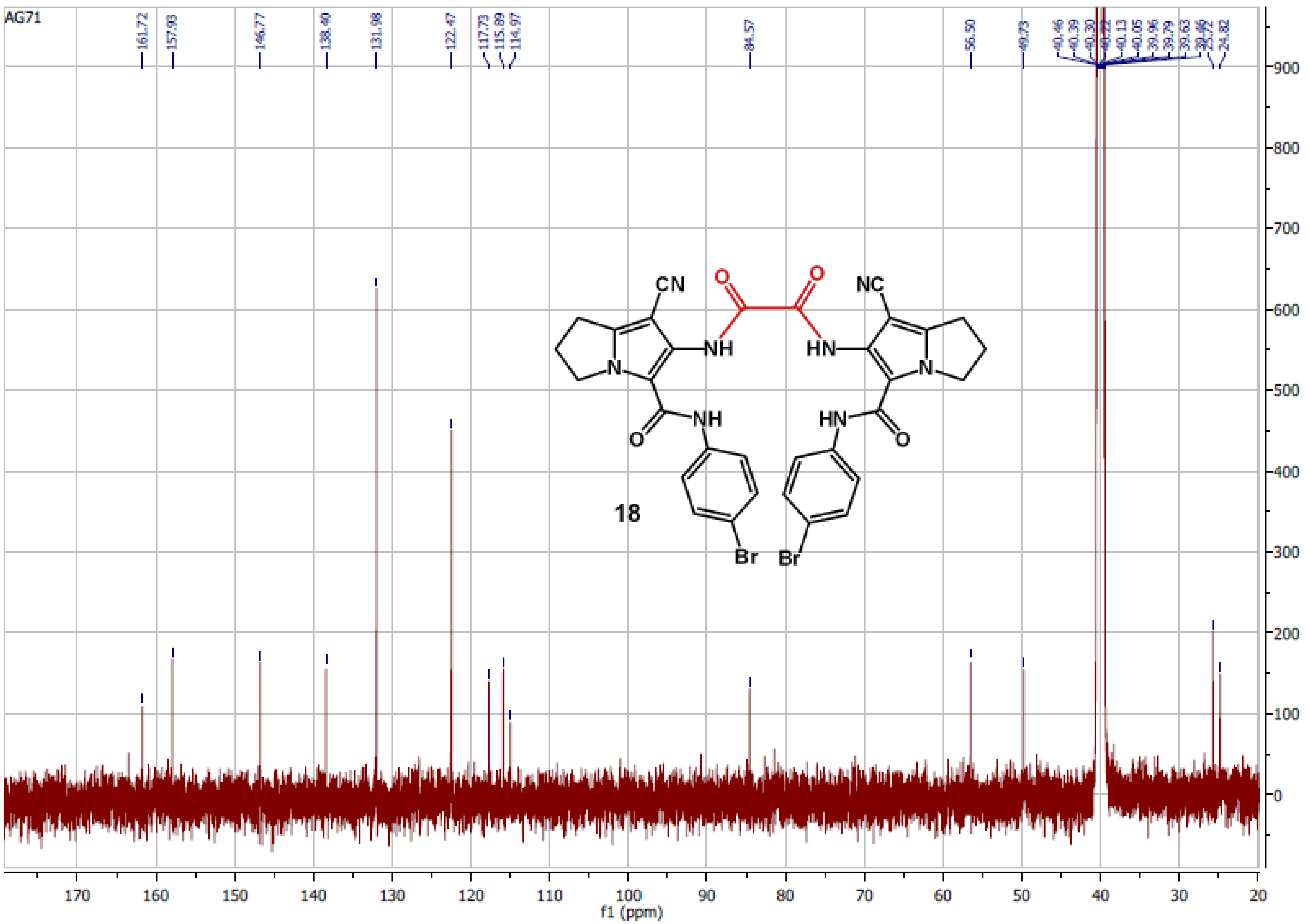

Figure S31: 13C-NMR (DMSO, 125 MHz, δ ppm) spectrum of compound 18

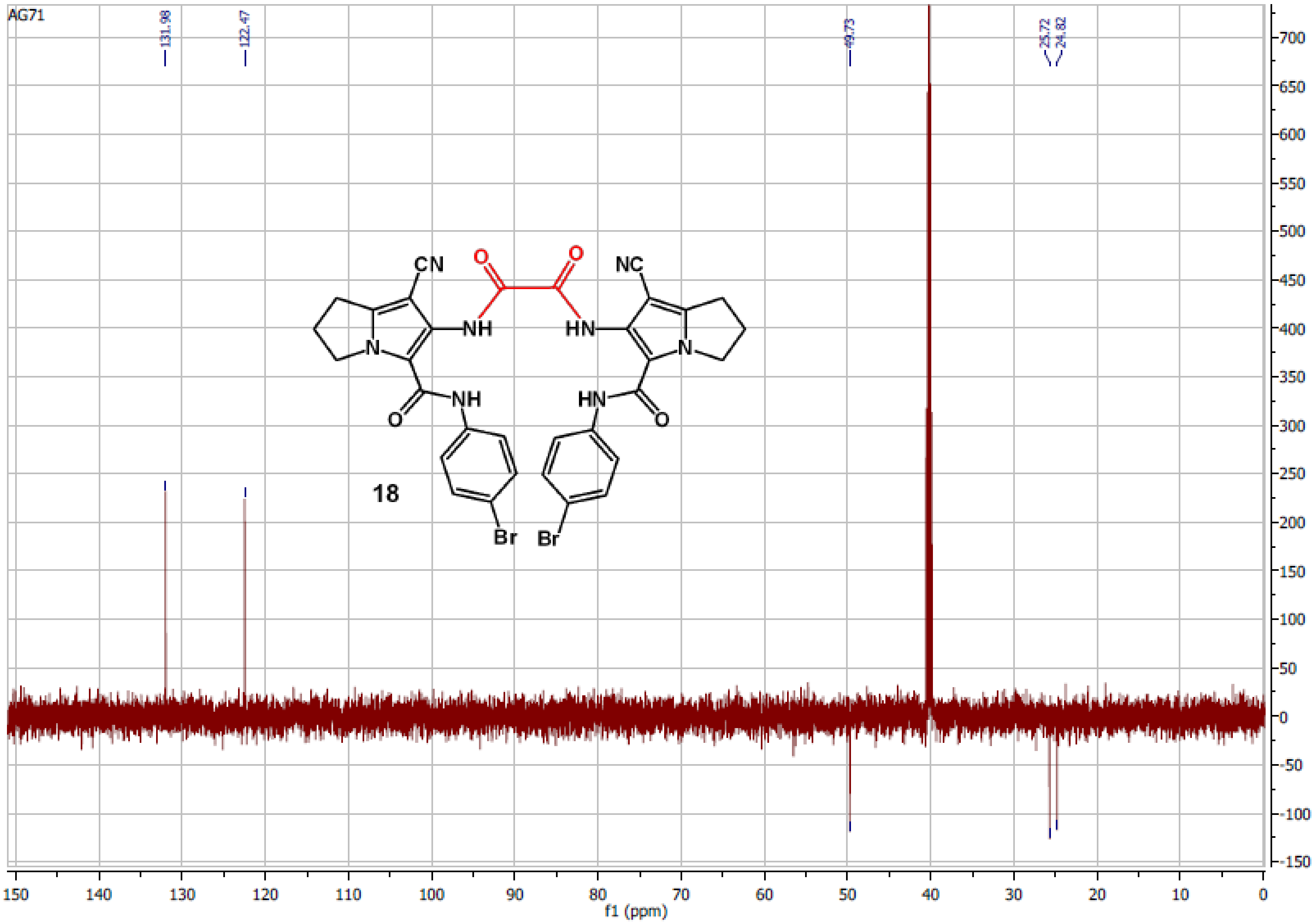

Figure S32: DEPT 135 spectrum of compound 18

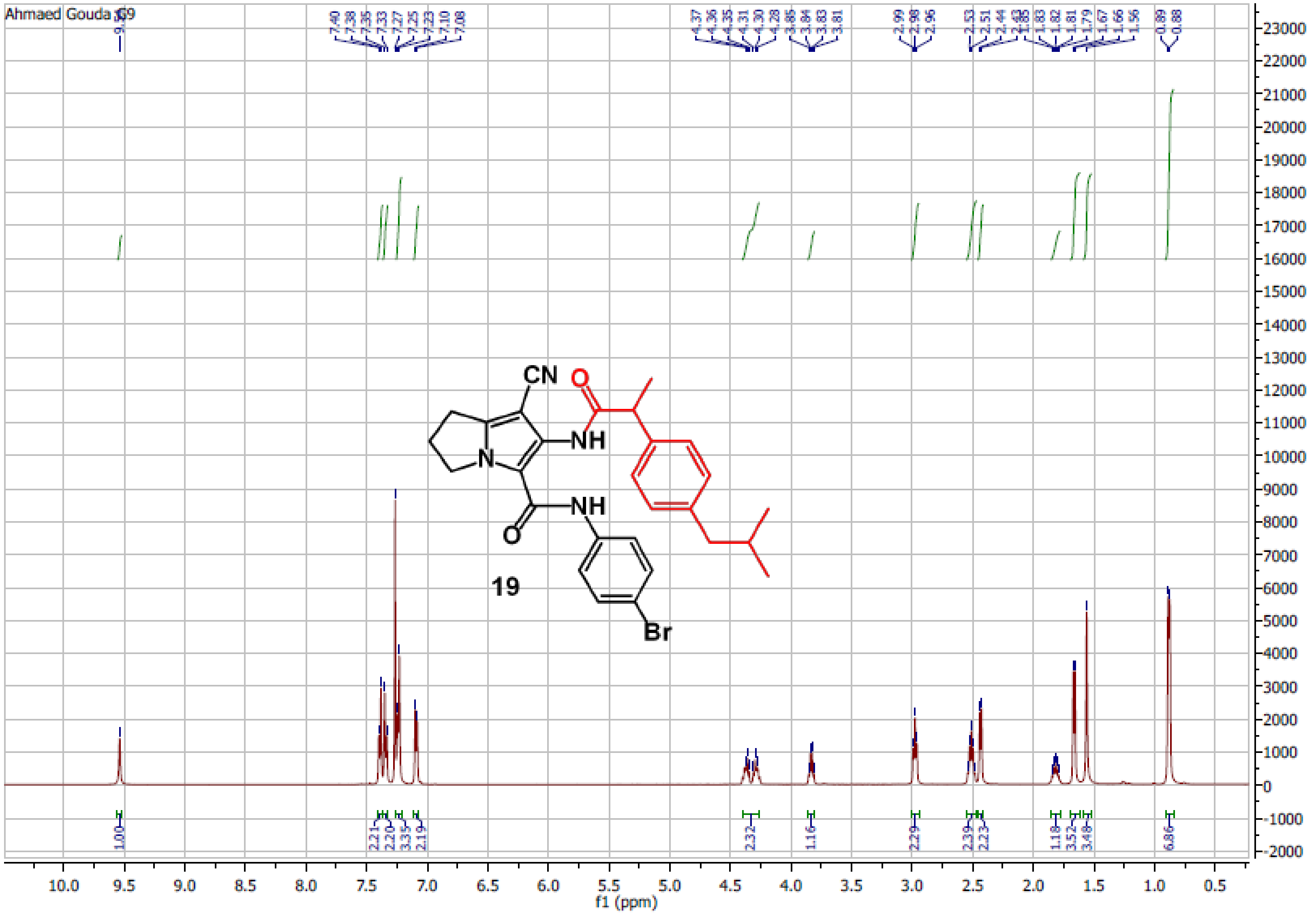

Figure S33a: 1H-NMR (CDCl3, 500 MHz, δ ppm) spectrum of compound 19

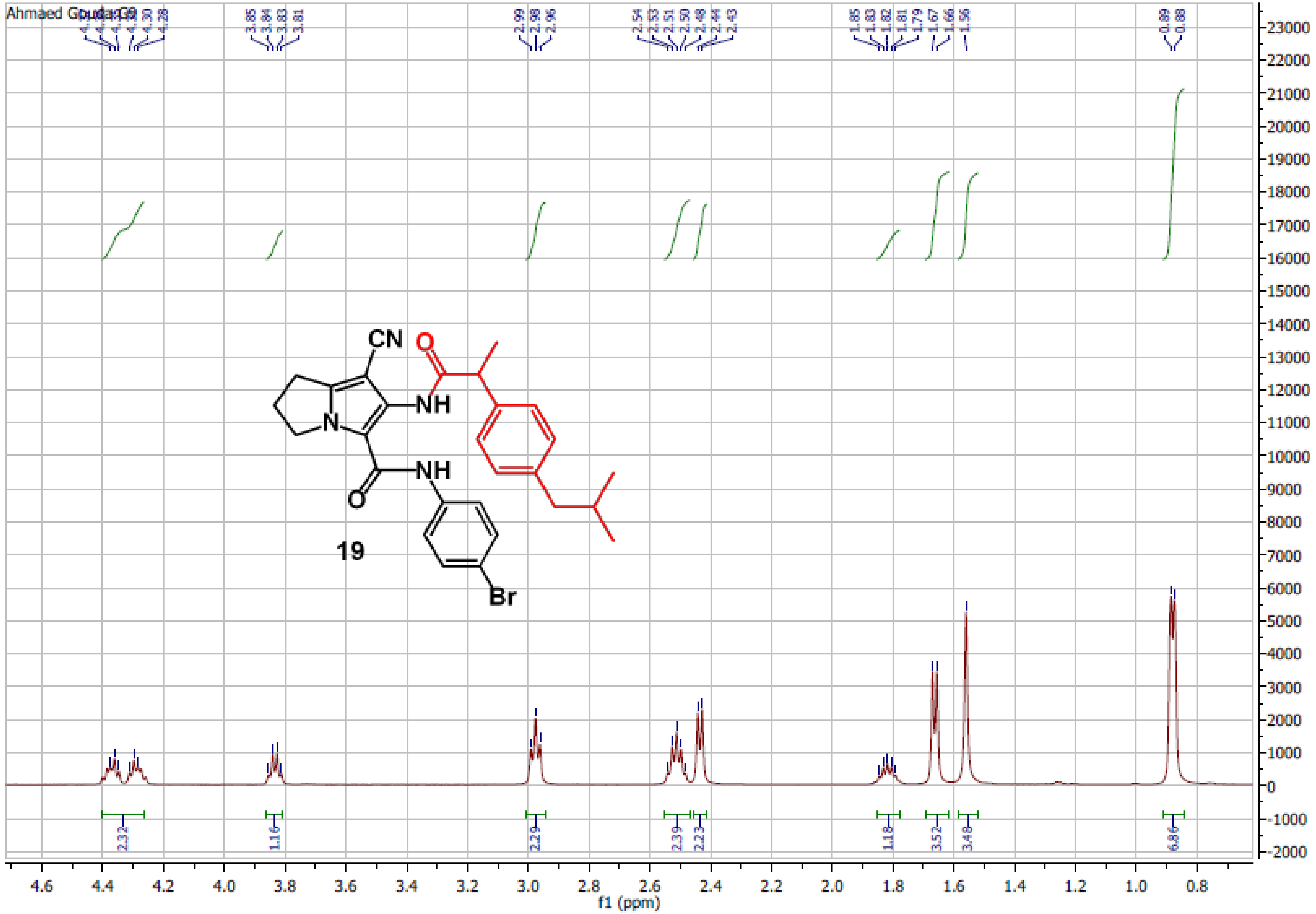

Figure S33b: 1H-NMR (CDCl3, 500 MHz, δ ppm) spectrum of compound 19 (ZOOM on Aliphatic Protons)

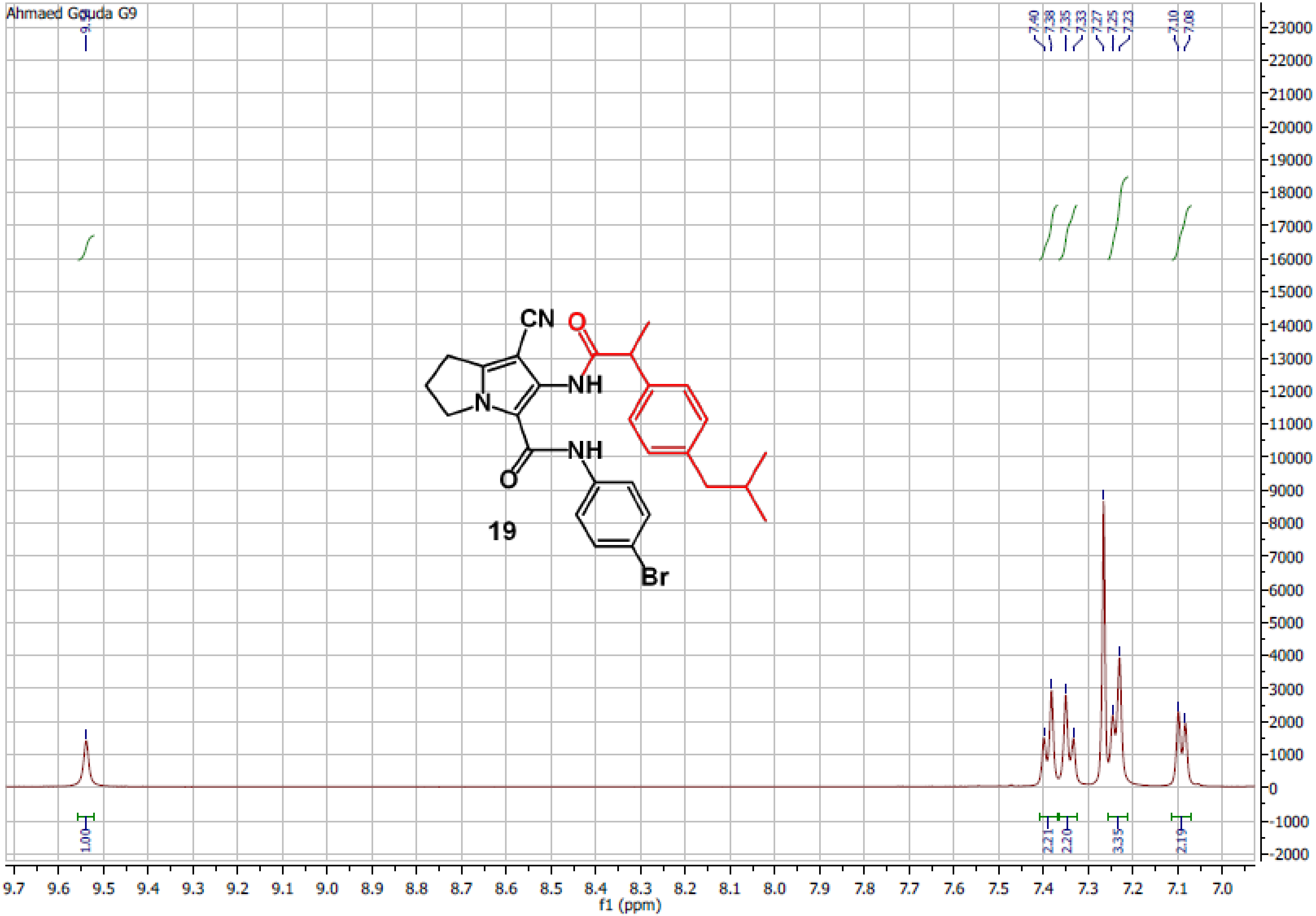

Figure S33c: 1H-NMR (CDCl3, 500 MHz, δ ppm) spectrum of compound 19 (ZOOM on Aromatic Protons)

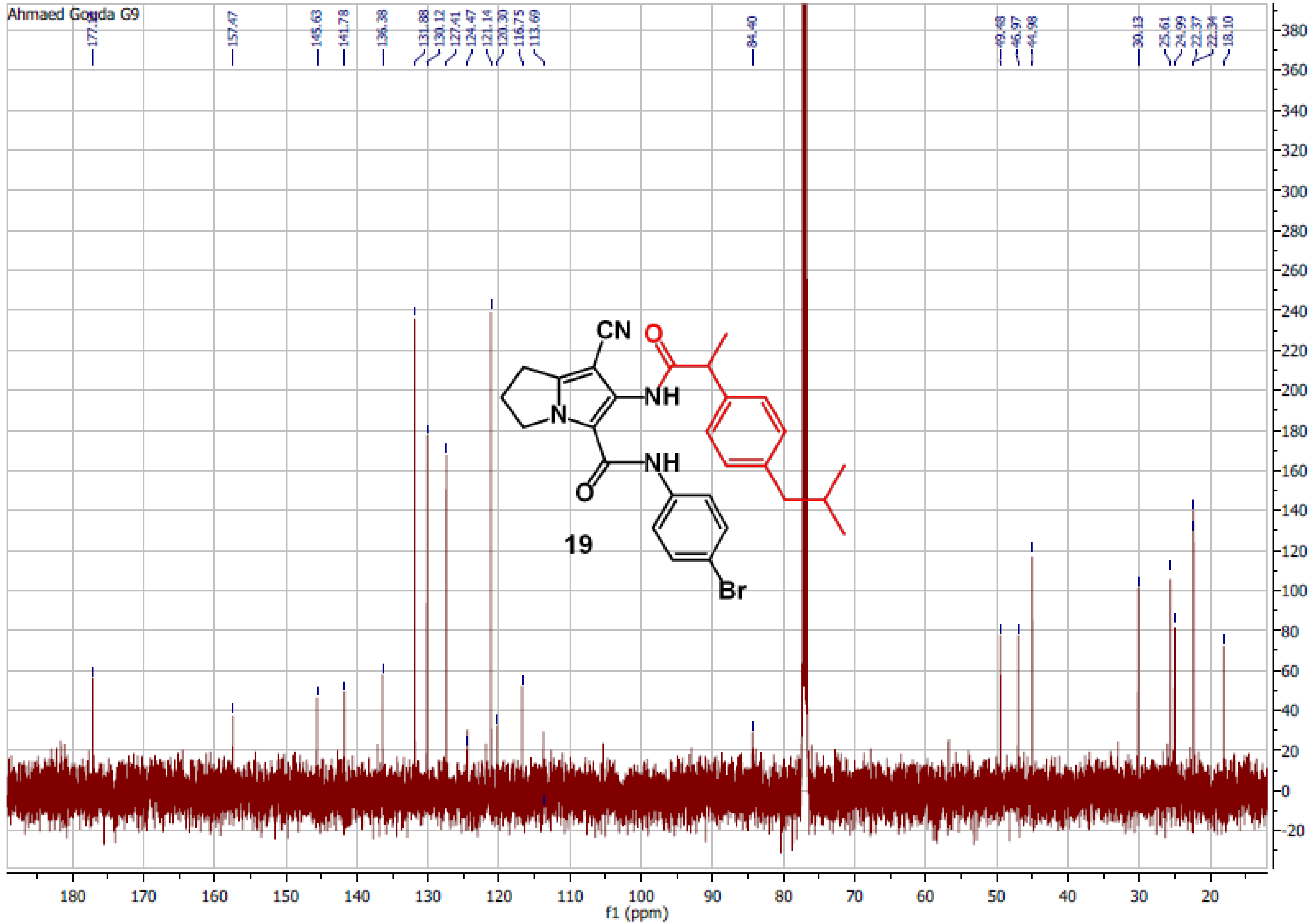

Figure S34a: 13C-NMR (CDCl3, 125 MHz, δ ppm) spectrum of compound 19

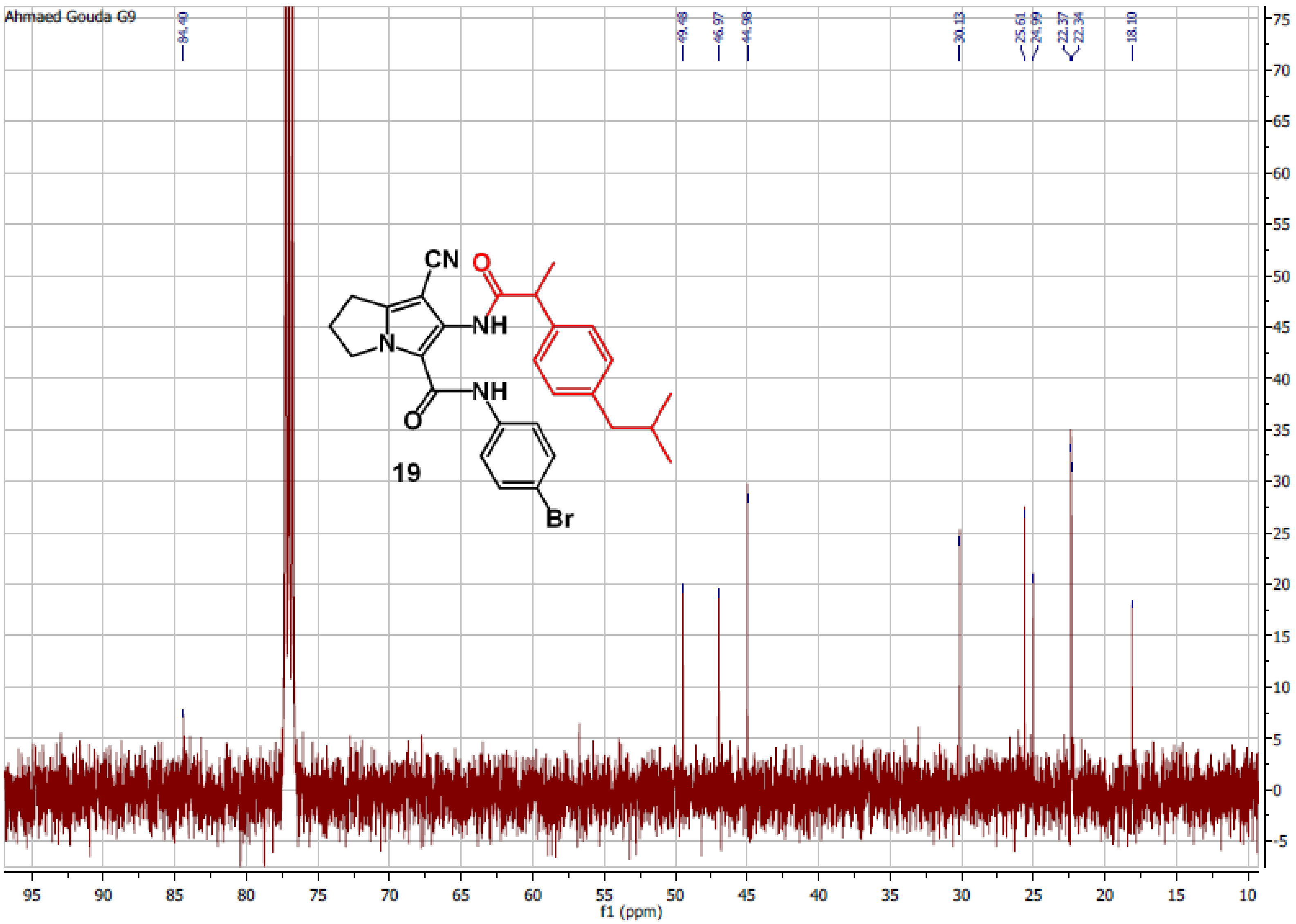

Figure S34b: 13C-NMR (CDCl3, 125 MHz, δ ppm) spectrum of compound 19 (ZOOM on Aliphatic Carbons)

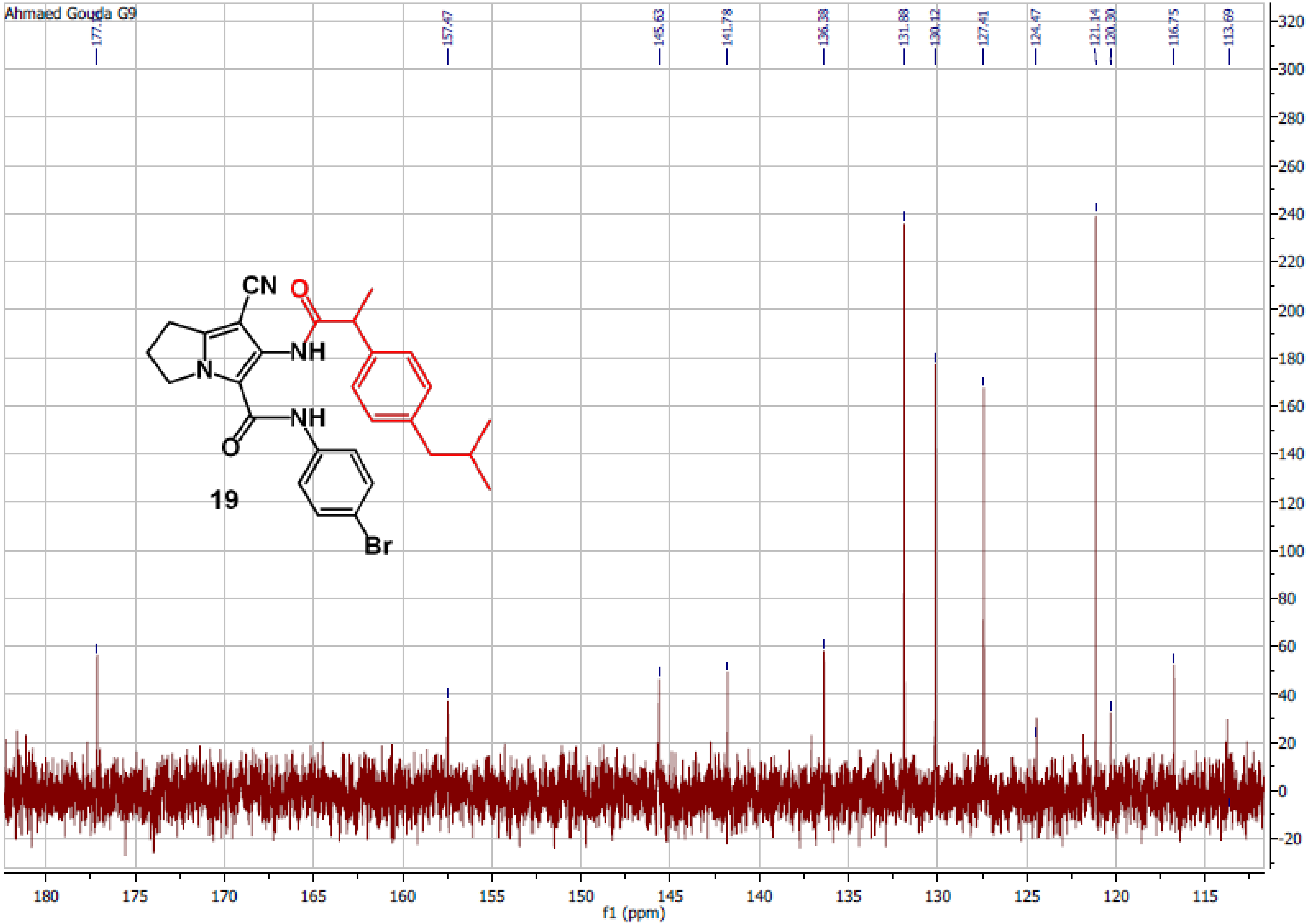

Figure S34c: 13C-NMR (CDCl3, 125 MHz, δ ppm) spectrum of compound 19 (ZOOM on Aromatic Carbons)

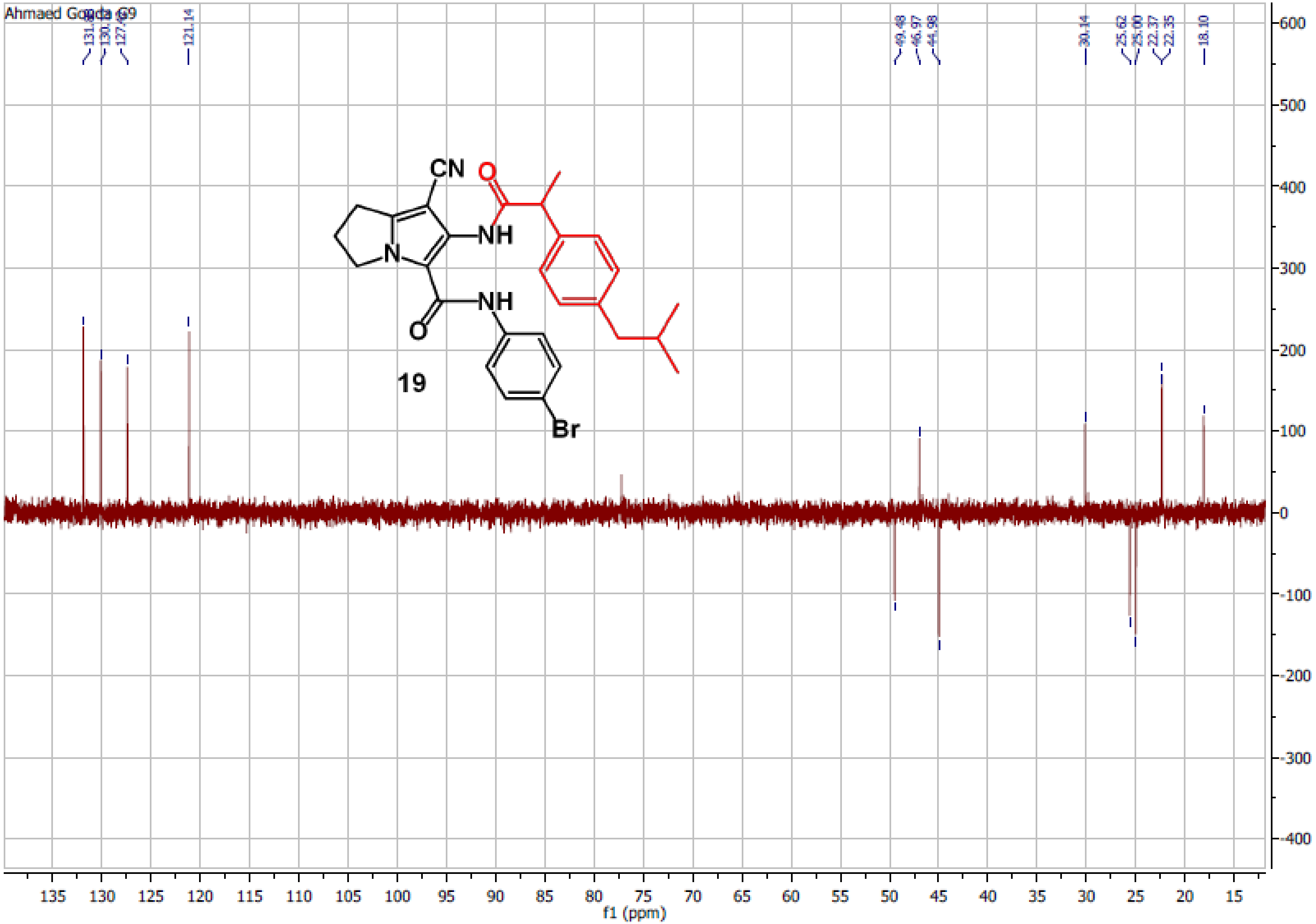

Figure S35: DEPT 135 spectrum of compound 19
